# Diamine Grafting of Pyrazole‐Based MOF‐303 for Diluted‐Source CO_2_ Capture

**DOI:** 10.1002/smll.202514197

**Published:** 2026-03-03

**Authors:** Giuseppe Mastronardi, Jacopo Perego, Charl Xavier Bezuidenhout, Wim Temmerman, Veronique Van Speybroeck, Valentina Crocellà, Silvia Bracco, Nello Li Pira, Angiolina Comotti, Silvia Bordiga

**Affiliations:** ^1^ Dipartimento Di Chimica Centro NIS Unità Di Ricerca INSTM Università Degli Studi di Torino Torino Italy; ^2^ Dipartimento Di Scienza dei Materiali Unità Di Ricerca INSTM Univesità Degli Studi di Milano‐Bicocca Milano Italy; ^3^ Center For Molecular Modelling Ghent University Zwijnaarde Belgium; ^4^ Centro Ricerche Fiat Orbassano Italy

**Keywords:** CO_2_ capture, metal organic framework, molecular modelling, spectroscopies

## Abstract

Amine‐functionalized materials have been extensively investigated for CO_2_ capture, with adsorption mechanisms based primarily on carbamate formation. Among the different strategies, grafting amine moieties onto porous solid supports represents a benchmark approach for developing highly effective adsorbents, even at very low CO_2_ concentrations. In this work, we propose a novel strategy for anchoring amine functionalities onto the surface of a Metal–Organic Framework (MOF), exploiting the unique chemistry/reactivity of the inexpensive and scalable MOF‐303. The pyrazole linkers in MOF‐303 serve as acidic anchoring sites for ethylenediamine molecules (EDAs), protonating one of the two amines to form an ammonium cation, which is then grafted to the pyrazolate moieties through charged‐assisted hydrogen bonds. The resulting MOF‐303#EDA exhibits a remarkable CO_2_ uptake of 0.71 mmol g^−^^1^ at 298 K and 450 ppm CO_2_. Notably, the high CO_2_ uptake of this material at 0.15 bar (2.5 mmol g^−1^) and its cyclability demonstrated in breakthrough experiments make it a promising candidate for point source CO_2_ capture industrial applications. The complex nature of amine grafting on MOF‐303 and its interaction with CO_2_ are investigated in depth by combining spectroscopic techniques (FT‐IR and SS‐NMR), synchrotron‐source X‐ray diffraction, and computational modeling.

## Graphical abstract

Vapor‐phase diffusion of ethylenediamine (EDA) into the pyrazole‐based MOF‐303 framework enables efficient amine grafting via pyrazole deprotonation and ion‐pair–stabilized hydrogen bonding with the pore walls. Spectroscopic analyses elucidate the interaction, while breakthrough experiments confirm the material's performance and cyclability.

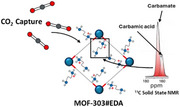

## 1 Introduction

Carbon dioxide (CO_2_) produced by the combustion of fossil fuels is widely recognized as the primary contributor to climate change and ocean acidification. Since the beginning of the industrial era in the 18th century, human activities have increased atmospheric CO_2_ concentrations to approximately 420 ppm, more than 150% above pre‐industrial levels (≈280 ppm in 1750) [1, 2]. Direct Air Capture (DAC) is increasingly regarded as a key strategy for reducing atmospheric CO_2_ levels. Currently, CO_2_ removal from ultra‐dilute sources is primarily achieved using aqueous amine solutions, which capture CO_2_ through a chemisorption mechanism involving the formation of carbamate and carbamic acid species [3, 4]. However, the regeneration of these liquid sorbents is highly energy‐intensive. As a result, solid sorbents, due to their relatively low heat capacities, are emerging as a more energy‐efficient and promising alternative for this application [5].

Metal–Organic Frameworks (MOFs) have attracted considerable attention due to their exceptionally high surface area, tunable pore sizes, and chemical versatility [6, 7]. These unique properties make them suitable for a wide range of applications, including carbon capture, gas storage, catalysis, and more [8, 9, 10, 11, 12, 13, 14]. Further functionalization of MOF pore surfaces with amine groups represents a benchmark strategy in the development of advanced solid sorbents for DAC technologies [15, 16]. Numerous studies in the literature report successful carbon capture enabled by amine‐functionalized MOFs. For instance, Fracaroli and co‐workers [17] proposed a series of isoreticular analogs of Mg‐MOF‐74, known as IRMOF‐74‐III‐(RNH_2_)_x_, in which pendant amine functionalities were introduced through linker modification. In their study, McDonald and colleagues [18] investigated several derivatives of M‐MOF‐74 (where M = Mg, Co, Fe, Zn, Ni, Mn), synthesized *via* functionalization of the open metal sites with N,N′‐dimethylethylenediamine (mmen). These functionalized frameworks exhibited notable CO_2_ uptake under ultra‐dilute conditions with a CO_2_ uptake of 2.0 mmol/g at 400 ppm. Another promising strategy involves the incorporation of polyethyleneimine (PEI) within MOF pores. For instance, Darunte and co‐workers [19] functionalized MIL‐101(Cr) with PEI, resulting in significantly enhanced CO_2_ adsorption (4.1 mmol/g at 15% of CO_2_) compared to pristine MIL‐101(Cr). Although the PEI‐functionalized material demonstrated lower performance at 400 ppm compared to the sample impregnated with tris(2‐aminoethyl)amine (TREN) (0.35 mmol/g at 400 ppm), MIL‐101(Cr)‐PEI‐300 exhibited superior cyclic stability.

**FIGURE 1:**
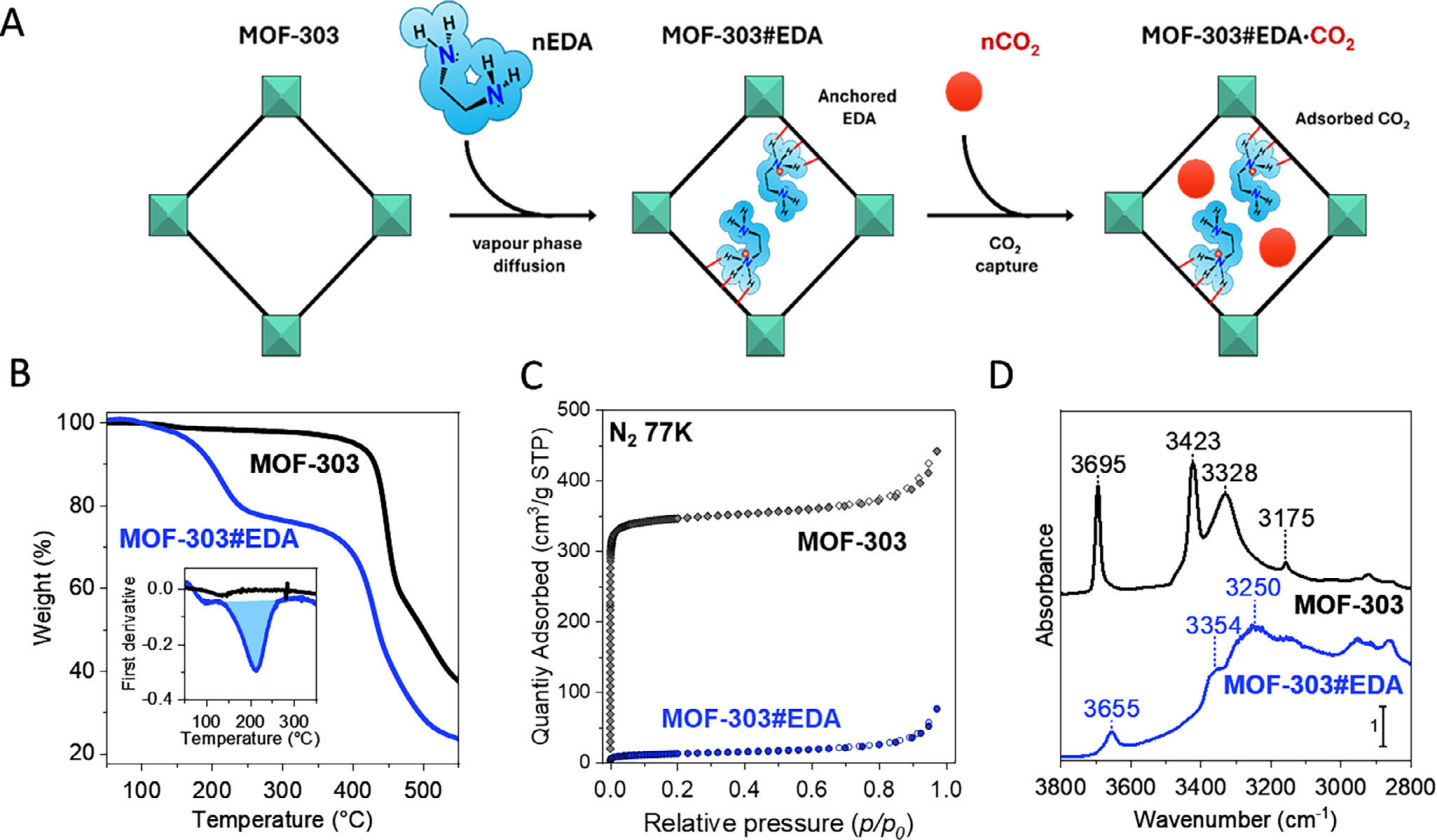
(A) Schematic illustration of the preparation of MOF‐303#EDA *via* diffusion of EDA molecules from the vapor phase and CO_2_ sorption in the MOF‐303#EDA sample. (B) Thermogravimetric analysis (TGA) of activated pristine MOF‐303 (black curve) and MOF‐303#EDA (blue curve) measured with a heating rate of 10°C min^−1^ under inert atmosphere (N_2_ flux, 50 mL min^−1^). The inset highlights the first derivative of the TGA profile between 50°C and 350°C. (C) N_2_ adsorption/desorption isotherms measured at 77 K of pristine MOF‐303 (black diamond) and MOF‐303#EDA (blue circle). Filled and empty symbols represent adsorption and desorption branches, respectively. (D) Comparison of IR spectra in the 3800–2800 cm^−1^ range of pristine MOF‐303 (black curve) and MOF‐303#EDA (blue curve) after outgassing at 150°C for 4 h and at room temperature for 1 h, respectively.

In recent years, many ionic liquids (ILs) incorporating amine functionalities have also been investigated for CO_2_ capture applications [20, 21, 22, 23]. Among these, ILs composed of pyrazole and diethylenetriamine (DETA) [24, 25] have shown promise due to their high CO_2_ uptake (11.39 mol CO_2_/kg IL) and reduced risk of leaching compared to pure amine. This enhanced performance is attributed to acid‐base interactions between pyrazole and DETA, which stabilize the system. Inspired by this concept, we propose a novel strategy to graft amine moieties within the pores of a MOF, by exploiting acid–base interactions with pyrazole groups. MOF‐303 has been chosen as a valuable host framework by virtue of the native presence of pyrazole inside the structure as part of the linkers. In principle, pyrazoles act as anchoring sites for amine moieties because of their intrinsic acidity, leading to an acid‐base reaction, mimicking the DETA/Pyrazole ionic liquid mentioned before. This interaction turned out to be particularly reinforced by the presence of a couple of pyrazoles facing each other, resulting in a strong binding site characterized by a charge‐assisted hydrogen bond. The use of diamine (Ethylenediamine, EDA) allowed us to tailor the CO_2_ capture performances of MOF‐303 toward direct air capture. The use of MOF‐303 turned out to be particularly profitable since this prototypical porous framework is prepared from cost‐effective building blocks under mild reflux conditions, it combines high thermal and water stability with a large surface area, and it is easily scalable [26]. The amino‐decorated MOF, denoted MOF‐303#EDA, was prepared *via* solvent‐free vapor‐phase diffusion of EDA. In contrast to the mentioned ionic liquid (which adopted diethylenetriamine), we propose the use of ethylenediamine by virtue of its lower boiling point, thereby facilitating vapor‐phase diffusion, and the low steric hindrance. This material exhibits excellent performance for selective CO_2_ capture under relevant industrial conditions and demonstrates good cyclability (Figure 1A). A comprehensive multi‐technique investigation, including synchrotron X‐ray diffraction, advanced spectroscopies, and computational modeling, provided detailed insight into the amine grafting process and unveils the CO_2_ adsorption mechanism of this newly functionalized MOF.

## 2 Results and Discussion

### 2.1 Pore Functionalization with Ethylenediamine

MOF‐303 was prepared according to a literature procedure using a hydrothermal strategy [26]. The sample was activated under high vacuum (*p* ≤ 3 µbar) at 150°C overnight to remove pre‐adsorbed molecules. The sample was then placed in a home‐made diffusion cell and exposed to EDA vapor for 3 days (see Experimental Section). An optimized activation protocol, in which the sample was treated at 60°C for 24 h under vacuum, enabled the removal of physisorbed amine, yielding the final sample, MOF‐303#EDA (Figure 1A). The presence of a strongly bonded EDA in MOF‐303#EDA was corroborated by thermogravimetric analysis (TGA). Figure 1B shows the TGA profile of both pristine MOF‐303 and MOF‐303#EDA measured under N_2_ atmosphere: in both samples, a minor weight loss (2–3%) below 130°C was observed and attributed to the desorption of a small amount of physisorbed water. The pristine MOF‐303 exhibits a stable plateau, followed by significant weight loss above 400°C, which corresponds to the thermal decomposition of the organic linker. In contrast, MOF‐303#EDA exhibits a substantial weight loss, which begins at about 130°C and reaches a peak at 214°C, a temperature much higher than the boiling point of EDA (T_b_ = 116°C; inset in Figure 1B; Figures S1 and S2). This event is attributed to the release of ethylenediamine molecules, which strongly interact with the pyrazolate moieties within the framework. From the experimental weight loss of 22.1 ± 1.1 wt.%, a molar ratio of 0.94 ± 0.06 EDA molecules per pyrazolate moiety was estimated, indicating a virtual stoichiometric 1: 1 ratio between these two moieties, as confirmed by quantitative ^13^C solid‐state NMR (see below). At temperatures above 350°C, the decomposition of the framework occurs.

The textural properties of the MOF were initially investigated by N_2_ adsorption/desorption isotherms at 77 K (Figure 1C). Pristine MOF‐303 exhibited a Type Ia isotherm, typical of microporous materials. The specific surface area (SSA) was calculated from the N_2_ adsorption isotherm by applying the Brunauer–Emmett–Teller (BET) [27] equation in the 0.01–0.025 *p/p*_0_ range, in accordance with the Rouquerol consistency criteria [28], and the Langmuir equation. For pristine MOF‐303, the resulting BET and Langmuir SSAs were 1469 and 1417 m^2^ g^−1^, respectively, in good agreement with values reported in the literature [26]. The micropore volume was 0.52 cm^3^ g^−1^, as obtained using the carbon slit‐pore model and NL‐DFT theory, and 0.50 cm^3^ g^−1^ according to the α_S_ method (Figure S3). These data align with the value of 0.54 cm^3^ g^−1^ estimated from the crystal structure, using a probe radius of 1.82 Å, which corresponds to N_2_ kinetic radius. The SSA of MOF‐303#EDA was drastically reduced (around 50 m^2^ g^−1^), in agreement with the presence of the grafted EDA, whose steric hindrance reduces the accessible pore volume for the N_2_ probe (Table S1).

The interaction between EDA and MOF‐303 was elucidated by comparing the IR spectra of MOF‐303 and MOF‐303#EDA (Figure 1D black and blue curves, respectively). The pristine MOF‐303 exhibits a sharp band at 3695 cm^−^^1^, assigned to the ν(O–H) stretching mode of the framework hydroxyl groups. A weaker band at 3175 cm^−^^1^ corresponds to the ν(C–H) stretching of the pyrazole ring, while two intense signals at 3423 and 3328 cm^−^^1^ are assigned to the ν(N–H) stretching modes of pyrazoles. The presence of two distinct N–H bands reflects the existence of two chemically distinct pyrazole environments in the framework. Upon EDA incorporation, the ν(O–H) stretching band of the hydroxyl groups is substantially eroded, with only a weak residual signal shifted by 40 cm^−^^1^. The characteristic ν(N–H) bands of the pyrazoles disappear, and a broad band centered at ∼3250 cm^−^^1^ emerges, testifying to an extensive H‐bonding, suggesting interaction between EDA and the MOF‐303 framework. The erosion of the ν(O–H) band implies that the hydroxyl moieties are directly involved in medium to strong H‐bonding interactions with EDA, with only a small fraction of OH remaining weakly perturbed (band at 3655 cm^−1^). Moreover, the disappearance of the pyrazole ν(N–H) signals confirms the deprotonation induced by EDA. The new band at 3354 cm^−^^1^ can be associated with ν(N‐H) stretching of ^+^NH_3_ moieties arising from protonated EDA. Additional intense bands below 2900 cm^−1^ correspond to ν(C–H) stretching vibrations and are consistent with the presence of C(sp^3^)–H groups from EDA.

Time‐resolved in situ IR experiment (Figure S4) further reveals that, as EDA vapors diffuse into MOF‐303 channel, the 3695 and 3423 cm^−^^1^ bands simultaneously decrease, while the bands at 3250 and 3354 cm^−^^1^ grow in intensity. This spectral evolution indicates interaction between EDA molecules and the hydroxyl moieties of the framework. Moreover, the gradual disappearance of the band at 3423 cm^−^^1^ suggests the possible deprotonation of pyrazole units. Figure S5 provides a mechanistic scheme regarding the EDA grafting onto the MOF‐303 pyrazole unit, based on IR spectra insight.

Despite the profound chemical changes at the surface of MOF‐303, SEM images (Figures S6 and S7) reveal that the morphology of MOF‐303 remains unaffected, with both MOF‐303 and MOF‐303#EDA consisting of nanocrystalline particles.

### 2.2 MOF‐303 Structure and Amine‐Framework Interactions

**FIGURE 2:**
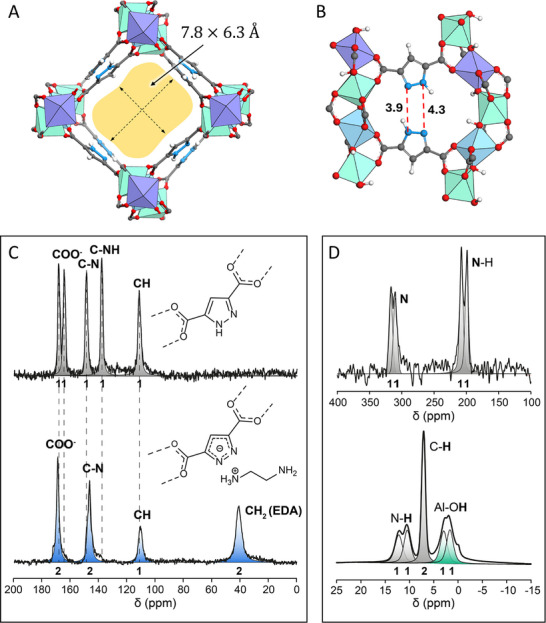
(A) MOF‐303 as viewed along the *a*‐axis. (B) A perspective view which shows the channel wall for MOF‐303. The hydrogen bonds are shown as red dashed lines, and the D⋅⋅⋅A distances (Å) are indicated. (C) Quantitative ^13^C SPE MAS NMR spectra of MOF‐303 (top) and MOF‐303#EDA (bottom) collected with a recycle delay of 60 s (D) ^1^H‐^15^N CP MAS spectrum collected with a contact time of 8 ms (top), and ^1^H SPE MAS spectrum of MOF‐303 collected with a recycle delay of 20 s (bottom). All solid‐state NMR spectra have been performed at 7.04 T and 295 K with a spinning speed of 12.5 kHz.

The crystal structure of MOF‐303 was solved by Rietveld refinement of X‐ray diffraction patterns collected using a synchrotron source (Figure S8). The framework of MOF‐303 is self‐assembled by trivalent aluminum (Al^3^^+^) centers, 1H‐pyrazole‐3,5‐dicarboxylate (PZDC) linkers, and hydroxide anions (Figure 2). The inorganic building units consist of chains of alternating *cis* and *trans* corner‐sharing AlO_4_(OH)_2_ octahedra, which propagate along the crystallographic *a* direction (Figure 2B). The shared corners of these chains are occupied by the OH groups, while the carboxylate moieties of the organic linkers bridge between adjacent aluminum centers, adding an additional connection between the octahedra and stabilizing the chain structure. These 1D Al‐oxide chains are further linked to the PZDC ligands to generate the 3D porous framework. Neighboring pairs of PZDC linkers form a weak N⋅⋅⋅H‐N hydrogen bond with a D⋅⋅⋅A distance of 3.9 Å (Figure 2B). The resulting pore structure comprises straight, 1D channels approximately 9 Å in diameter. These channels run parallel to the chains of corner‐sharing octahedra and are lined with both the pyrazolate rings and hydroxyl groups from the inorganic units.

^13^C SPE MAS NMR spectrum of the pristine MOF‐303 reveals five signals of comparable intensity, each corresponding to the five distinct carbon atoms of the pyrazole dicarboxylate ligands. In contrast, MOF‐303#EDA spectrum displays a markedly different profile (Figure 2C; Figure S9). Upon incorporation of EDA and the formation of MOF‐303#EDA, deprotonation of the pyrazole rings makes the two nitrogen atoms chemically equivalent, increasing the symmetry of the ring and reducing the number of signals to three with 2:2:1 intensity. Owing to their equivalence from the constitutional point of view, single signals are generated by the carbons covalently bonded to the nitrogen atoms and the carboxylate carbons at δ = 146.9 and 169.2 ppm, respectively. The overall data suggest that the ^13^C solid‐state NMR multiplicity observed in pristine MOF‐303 is largely governed by the protonation state of the pyrazoles, whilst upon deprotonation, local symmetry dramatically increases, resulting in a substantial reduction of NMR signals.

Evidence of two crystallographically independent pyrazole molecules in MOF‐303 is provided by observing the ^15^N nucleus of unlabeled MOF‐303, whose spectrum shows split signals for both N and N‐H groups, centered at about δ = 313.6 and 203.4 ppm, respectively (Figure 2D, top). ^1^H MAS NMR spectrum shows two N‐H resonances with a large difference in chemical shift of δ = 1.7 ppm, confirming the existence of two distinct interactions for the N‐H group (Figure 2D, bottom). Additionally, the ^1^H MAS NMR spectrum exhibits two signals with a 1:1 ratio at δ = 1.7 and 2.9 ppm, which correspond to the two symmetry‐independent OH groups, each in a unique environment in agreement with the crystal structure, and a sharp resonance at δ = 7.1 ppm, which arises from the CH hydrogens of pyrazole. In MOF‐303#EDA, the N‐H signal of pyrazole completely disappears (Figure S10), demonstrating its full deprotonation upon EDA incorporation. Importantly, the ^1^ ^3^C SPE MAS NMR spectrum of MOF‐303#EDA (Figure 2C, bottom) enables the quantification of the EDA molecules grafted into the pores. Analysis of the peak areas unveils a 1:1 ratio between EDA and pyrazolate units, in excellent agreement with the TGA analysis, leading to the formation of one ammonium moiety per EDA molecule. In comparison, the ^13^C SPE MAS NMR of the MOF‐303#EDA before the 60°C thermal treatment described above (Figure S11) highlights a slight excess of EDA, confirming the presence of labile physisorbed EDA (1.6: 1 ratio between EDA and pyrazolate unit), corroborating the previous finding from thermogravimetric analysis. Detailed peak assignments are provided in Tables S2 and S3.

Starting with the structural information provided by solid‐state NMR and IR spectroscopy, a DFT model of MOF‐303#EDA was constructed, inserting 8 EDA molecules into the MOF‐303 crystal cell, and thus respecting the pyrazolate/EDA ratio determined by ^1^ ^3^C SPE MAS NMR. Following DFT geometry optimization, the identified minima yielded the **S_0_** structure. The **S_0_** structure involves two symmetrical‐unique EDA molecules, EDA‐1 and EDA‐2, located near the nitrogen atoms of the PZDC linkers with *gauche* conformations (Figure S12). These EDA molecules act as bases, deprotonating the pyrazole NH groups of the framework to generate pyrazolate anions and one ammonium (NH_3_^+^) cation for each EDA molecule. Thus, the EDA molecules interact with the neighboring PZDC linkers to form strong N⋅⋅⋅H‐N(NH_3_^+^) charge‐assisted interactions. Notably, these interactions create a strong binding affinity of the EDA molecules to the walls of the MOF framework. Additionally, EDA‐1 forms an O‐H⋅⋅⋅N hydrogen bond with its NH_2_ group and the OH of the 1D node. All these interactions contribute to the overall stabilization of the MOF‐303#EDA assembly. The energies of adsorption for EDA‐1 and EDA‐2 molecules from the gas‐phase onto MOF‐303 are attested to be −221.5 and −178.1 kJ mol^−1^, respectively, giving insight into the strength of this type of anchoring site and its major stability compared to the reactants. Interestingly, the deprotonation of the pyrazole occurs spontaneously during geometry optimization, suggesting a barrier‐free process. The N‐H distance of the pyrazole significantly increases, indicating the breaking of the N‐H bond in favor of amine protonation (Figure S13).

^13^C MAS NMR spectra of MOF‐303 and MOF‐303#EDA were simulated following Plane Wave (PW)‐DFT chemical shielding tensor calculations (Figure S14). The simulated spectrum of MOF‐303 (based on the DFT optimized structure of MOF‐303) shows excellent agreement with the experimental data. In particular, in MOF‐303, the asymmetric environment of the pyrazole moieties, manifested experimentally by the presence of two pyrazole signals for carbons covalently bonded to nitrogen, *i.e*., C‐N and C‐NH, is clearly reproduced in the simulations. Upon introduction of EDA (structure **S_0_**), the pyrazolate carbon signals merge, thereby reinforcing our earlier assumption that the multiplicity of the ^13^C solid‐state NMR signals in MOF‐303 is governed by the protonation state of the pyrazole groups.

**FIGURE 3:**
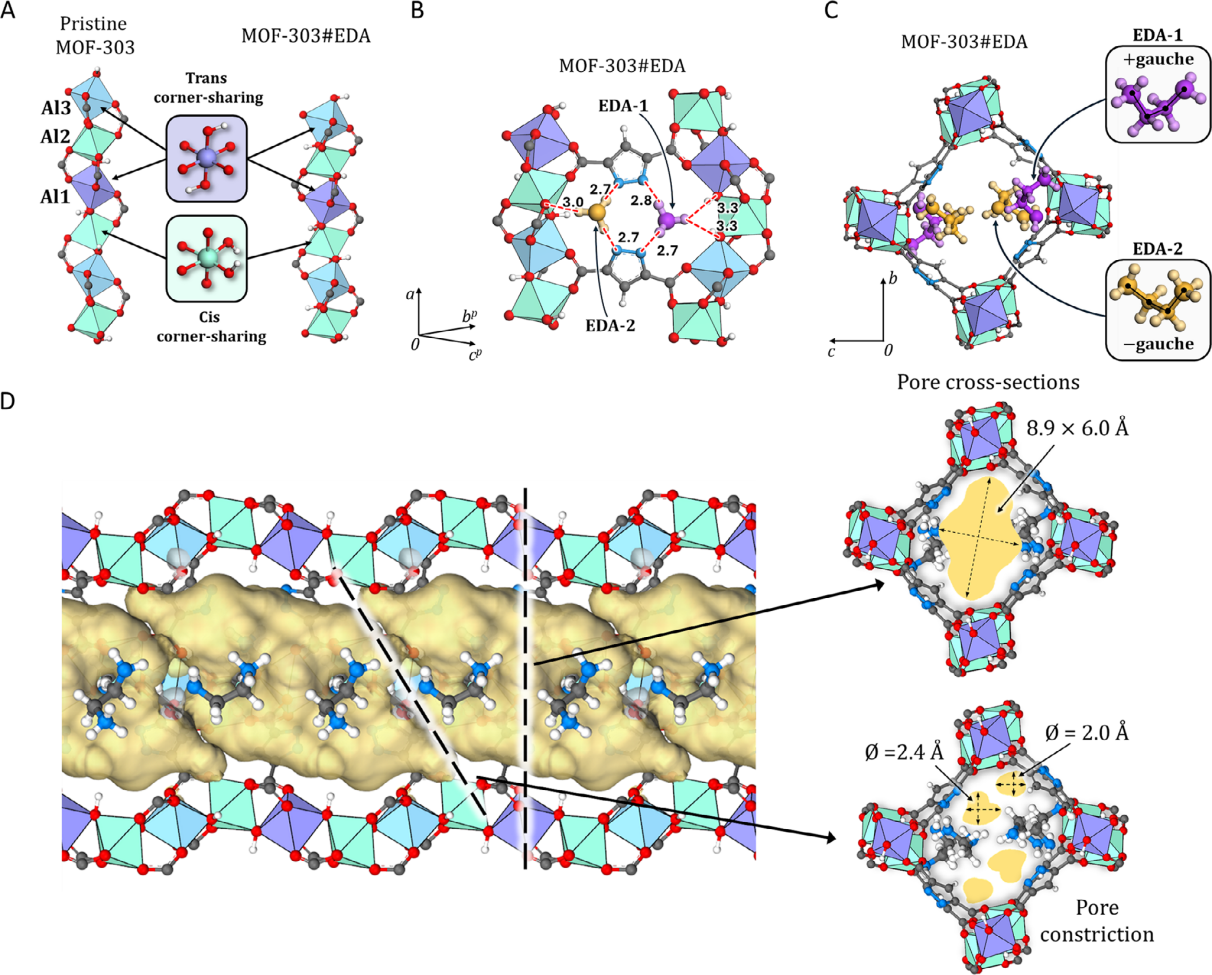
(A) A comparison of the 1D AlO_4_(OH) nodes of pristine MOF‐303 (Al_2_(OH)_2_PZDC_2_) and MOF‐303#EDA (Al_2_(OH)_2_PZDC_2_EDA_2_) with AlO_4_(OH)_2_ octahedra shown in different colors and the *trans* and *cis* corner‐sharing octahedra highlighted as derived from Rietveld refinement. (B) A perspective view which shows the channel wall for the refined MOF‐303#EDA structure. For clarity, only the ‐NH_3_^+^ groups of the two EDA molecules are shown to indicate charge‐assisted hydrogen bonds formed with the MOF framework. (C) Refined structure of MOF‐303#EDA as viewed along the *a*‐axis, the two symmetry‐independent EDA molecules are shown in purple (EDA‐1) and orange (EDA‐2) with an enlargement to highlight their conformation. (D) (left) The pores of the MOF‐303#EDA shown in yellow were mapped using a probe radius of 1.2 Å. The dashed line indicates the position of the pore cross‐section as shown on the right. The cross‐section dimensions and constriction diameters are indicated. Al *trans* and *cis* corner‐sharing: dark violet and green, respectively; PZDC: carbon gray, nitrogen blue, hydrogen white. EDA‐1: violet, EDA‐2: yellow.

The synchrotron‐source powder XRD pattern of MOF‐303#EDA reveals the emergence of the (020) and (002) reflections, which are absent in the XRD pattern of the pristine MOF‐303 (Figure S15). These diffraction peaks can be attributed to the inclusion of EDA molecules in the channels of the MOF, *i.e*., an increase in electron density within the MOF channels. Starting from structure **S_0_**, the crystal structure of loaded MOF‐303 with ethylenediamine (EDA) molecules was solved by Rietveld Refinement combined with DFT optimization (Figure S16 and Table S4), resulting in a shrinking of the *a*‐axis of 1.25 Å (Figure 3A; Figures S17 and S18). This shortening can also be observed in the 1D metal node, especially the *cis* corner‐sharing AlO_4_(OH)_2_ octahedra, wherein the corner‐to‐corner distance shortens by 0.4 Å, which accounts for 60% of the shrinkage along the *a*‐axis. Furthermore, both the *cis* and *trans* corner‐sharing octahedra show an increased tilt compared to the *a*‐axis of 11.6° and 4.1°, respectively, resulting in a further shortening of the *a*‐axis. A comparison of MOF‐303 and MOF‐303#EDA structure is provided in Figures S19–S21.

The shrinkage observed for the 1D node and *a*‐axis could be related to hydrogen bonds involving the ammonium cations with carboxylate oxygen and hydroxide oxygen atoms in the 1D Al‐based inorganic chains. Specifically, EDA‐1 forms (NH_3_^+^)N‐H⋅⋅⋅O interactions with both OH groups of the *cis* corner‐sharing octahedra, with DA distances of 3.3 Å each, which could be responsible for shortening the OH⋅⋅⋅OH distance (corner‐to‐corner) of the *cis* corner‐sharing octahedra (Figure 3B). The total pore volume (V_pore_) for MOF‐303#EDA was estimated to be 653.5 Å^3^ (using a probe of 1.2 Å), comprising large pores with a cross‐section of 8.9 × 6.0 Å^2^ that are connected by small constrictions, along the *a*‐axis, with diameters of 2.0–2.4 Å^2^. These constrictions are formed by two EDA‐2 molecules, which interact with one another across the channel (Figure 3C,D).

### 2.3 CO_2_ Adsorption and Spectroscopic Insights Under Controlled Atmosphere

**FIGURE 4:**
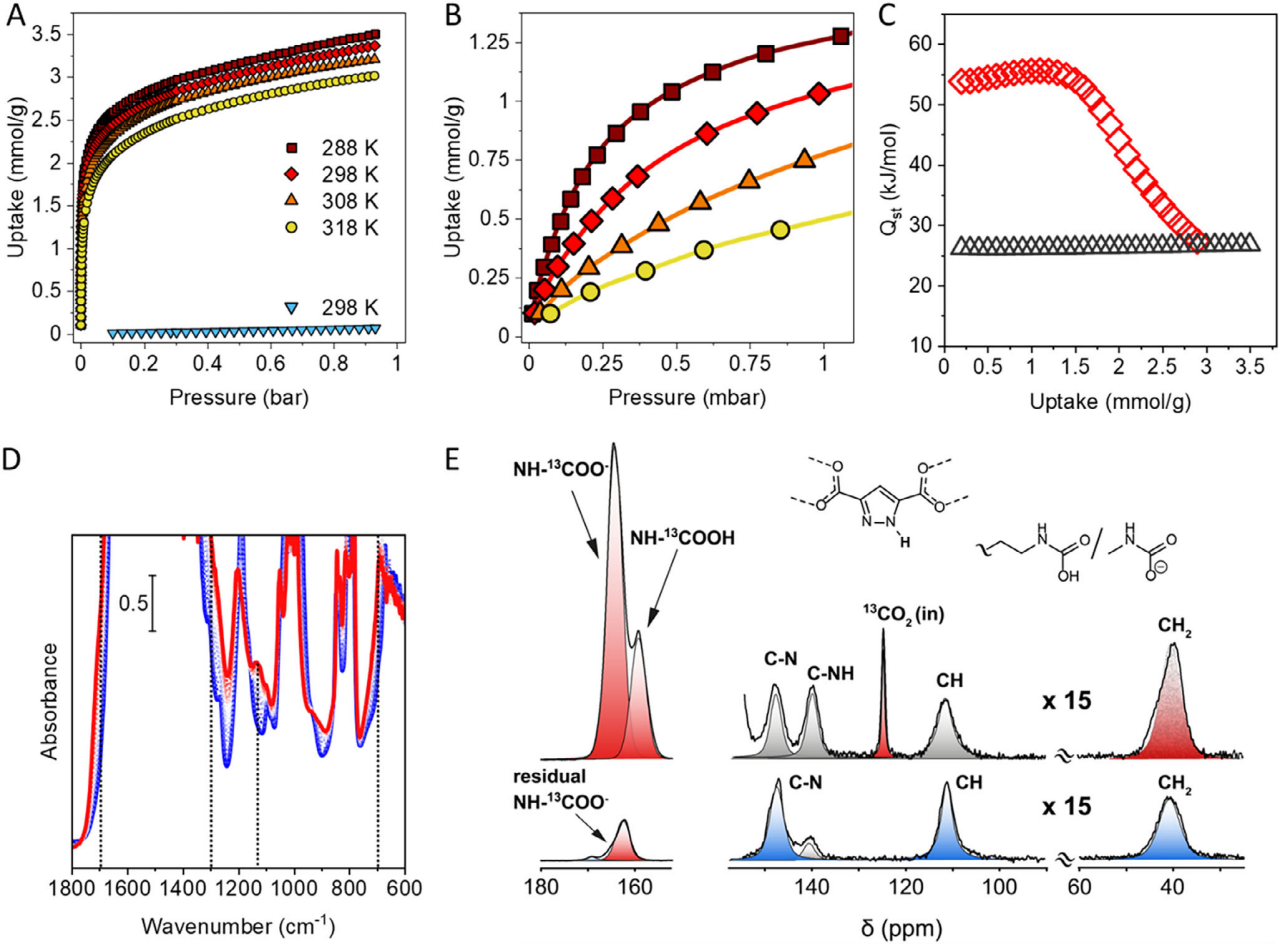
(A) CO_2_ adsorption isotherms of MOF‐303#EDA collected at 288 K (dark red square), 298 K (red diamonds), 308 K (orange triangle), and 318 K (yellow circle). N_2_ adsorption isotherm collected at 298 K (down‐pointing light blue triangle). Only the adsorption branches are shown for clarity. (B) Enlargement of CO_2_ adsorption isotherms between 0 and 1.125 mbar. (C) CO_2_ isosteric heat of adsorption of MOF‐303#EDA (red diamonds) and MOF‐303 (gray triangle) calculated from the adsorption isotherms collected at different temperatures according to the Van 't Hoff equation. (D) IR spectra of MOF‐303#EDA in the 1800–600 cm^−^^1^ region after CO_2_ adsorption at increasing pressures (0.2–120 mbar, from blue to red curves). Dotted lines indicate bands associated with carbamate formation. (E) (top) ^13^C CP MAS NMR spectra of MOF‐303#EDA^.^ loaded with enriched‐^13^C CO_2_ (99%) at 295 K and 760 Torr and (bottom) after evacuation at 10^−6^ bar for 24 h and thermal treatment at 60°C for 6 h. The intensity of the region from 150 to 10 ppm was multiplied by 15. ^13^C CP MAS NMR spectra have been performed at 7.04 T and 290 K with a spinning speed of 11.0 kHz.

The CO_2_ adsorption performances of pristine MOF‐303 and MOF‐303#EDA were evaluated by collecting CO_2_ and N_2_ adsorption isotherms at different temperatures. These results confirm that incorporation of the amine moiety significantly alters the adsorption profile of MOF‐303#EDA compared to the parent compound, greatly enhancing the CO_2_ uptake at pressures below 0.5 bar. (Figures S22–S26). The CO_2_ adsorption isotherms of MOF‐303#EDA collected at 288, 298, 308, and 318 K display a steep uptake at very low pressures (Figure 4, panels A and B), indicating strong interactions between the CO_2_ molecules and the pore walls. Notably, at 298 K, MOF‐303#EDA shows a significant uptake of 0.71 mmol g^−1^at 450 ppm (0.45 mbar) CO_2_ and 1.03 mmol g^−1^ at 1000 ppm (1.0 mbar) (Figure 4B), highlighting its potential for CO_2_ capture from ultra‐dilute sources. Above 100 mbar, the slope of the isotherms of MOF‐303#EDA rapidly decreases, approaching a plateau, consistent with saturation of high‐affinity binding sites. Indeed, from the crystal structure of MOF‐303#EDA, the CO_2_‐accessible volume calculated using the kinetic radius of CO_2_, yields a value of 486.8 Å^3^, which can accommodate 7.4 MPU (molecules per unit cell) (0.925 CO_2_ per EDA, 3.58 mmol g^−1^) of CO_2_, in excellent agreement with the maximum sorption capacity at 1 bar and 288 K (3.51 mmol g^−1^). These results confirm that EDA incorporation introduces high‐affinity adsorption sites that enhance CO_2_ uptake at low pressure, thereby improving the performance for carbon capture from point sources and DAC; the performance metrics have been summarized in Table S5. The reversibility of carbamates and the restoration of performance of MOF‐303#EDA in these conditions were confirmed by CO_2_ isotherm after degassing at 60°C in vacuum (Figure S27). Moreover, MOF‐303#EDA shows negligible N_2_ adsorption at 298 K, with a maximum uptake of 0.06 mmol/g at 0.93 bar and 298 K (Figure 4A).

The enthalpic isosteric heat of adsorption for CO_2_ (Q_st_) was determined using the Van't Hoff equation (Figure 4C; Figure S28) from the adsorption isotherms measured at different temperatures. The analysis yielded a maximum Q_st_ value of 55 kJ·mol^−^^1^ for MOF‐303#EDA, consistent with chemisorption and comparable to other amine‐functionalized porous frameworks. The CO_2_ uptake value of MOF‐303#EDA, measured at 150 mbar and 298 K, surpasses that of several amino‐loaded MIL‐101 compounds and is comparable to nmen‐Mg_2_(dobpdc) and CuBTTri‐mmen (see Figure S29 and Table S6). The Q_st_ value of MOF‐303#EDA (55 kJ·mol^−^^1^) appears lower than most of the previously reported materials, and it is demonstrated to be extremely advantageous because it balances the strong low‐pressure binding with regeneration requirements of mild conditions, consistent with the 68°C desorption temperature. Pristine MOF‐303, in contrast, shows a maximum isosteric heat of adsorption of 26 kJ·mol^−^^1^ (Figure S30). This value lies within the range typically associated with mild physisorption [29, 30, 31].

CO_2_ adsorption was further investigated through in situ IR spectroscopy using a custom‐built setup designed for controlled gas‐dosing experiments (Figure 4D). The IR spectra of MOF‐303#EDA were recorded under vacuum and subsequently upon progressive exposure to increasing CO_2_ pressures, from 0.2 to 120 mbar. This approach aimed to monitor the potential formation of carbamic acid or carbamate species during CO_2_ adsorption. Upon CO_2_ dosing, new bands indicative of carbamate formation within the MOF micropores appeared. Specifically, the shoulder at 1680 cm^−^^1^ and the weak signal at 1310 cm^−^^1^ are consistent with the asymmetric ν_asym_(OCO) and symmetric ν_sym_(OCO) stretching vibrations of the carbamate group. A further shoulder at 690 cm^−^^1^ can also be associated with carbamate formation, in line with previous observations [32, 33, 34, 35]. The IR spectra in the 3800–1700 cm^−1^ range during CO_2_ loading are shown in Figure S31.

The desorption of CO_2_ from MOF‐303#EDA was also investigated under the same experimental conditions (Figure S32). Interestingly, a degassing at room temperature for 2 h completely restores the original spectrum, confirming the reversibility of CO_2_ binding (Figure S33).

^13^C MAS NMR spectra of MOF‐303#EDA loaded with enriched ^13^CO_2_ (denoted MOF‐303#EDA·CO_2_) at room temperature and 1 bar enabled us to investigate in detail the newly formed chemical species (Figure 4E). Indeed, two distinct resonances at δ = 163.9 and 159.0 ppm were detected, corresponding to carbamate (‐NH^13^COO^−^) and carbamic acid (‐NH^13^COOH) with a ratio of 3: 1, respectively [36, 37, 38, 39, 40, 41]. The ratio of approximately 3:1 is typically observed for rather high CO_2_ loading in porous materials containing alkylamines at room temperature and 1 bar in an anhydrous environment, as reported in ref. [28]. A negligible amount of enriched ^13^CO_2_ physisorbed inside the pores (1.4%) is observed at δ = 124.4 ppm [42, 43]. From the ^13^C MAS NMR spectrum collected with a recycle delay of 60 s, the quantification of reacted ‐NH_2_ in MOF‐303#EDA to form carbamate and carbamic was estimated from the signal of CH_2_ moieties and resulted to be 76%, demonstrating a massive conversion (Figure S34). Simultaneously, the pyrazolate group was protonated, as unveiled by the two distinct chemical shifts at δ = 139.5 and 147.3 ppm for the C‐NH and C‐N carbons of the ligand, respectively. The formation of carbamic acid was confirmed by a ^1^H MAS NMR spectrum collected at 600 MHz and 30 kHz spinning speed, which showed a ‐^13^COOH resonance at δ = 14.7 ppm (Figure S35). After exposure of MOF‐303#EDA**·**CO_2_ to 0.001 mbar vacuum, the signal of carbamic acid was drastically reduced to a negligible amount (<6%), whilst the carbamate resonance decreased to 13% after subsequent thermal treatment at 60°C under vacuum, demonstrating the reversible release of CO_2_. Notably, CO_2_ desorption occurs simultaneously with deprotonation, leading to the formation of the pyrazolate moiety, as evidenced by the disappearance of the C‐NH signal of pyrazole moiety (Figure S36). Further information about peak assignments is shown in Table S7. To demonstrate the efficient capture of CO_2_ under realistic conditions, the formation of carbamate was followed in situ by IR spectroscopy by exposing MOF‐303#EDA to the open air in the presence of humidity (RH = 25%). The carbamate formation was followed in situ by IR (ATR) spectroscopy as shown by the appearance of the two typical bands at 1645 and 1350 cm^−1^ (Figure S37).

**FIGURE 5:**
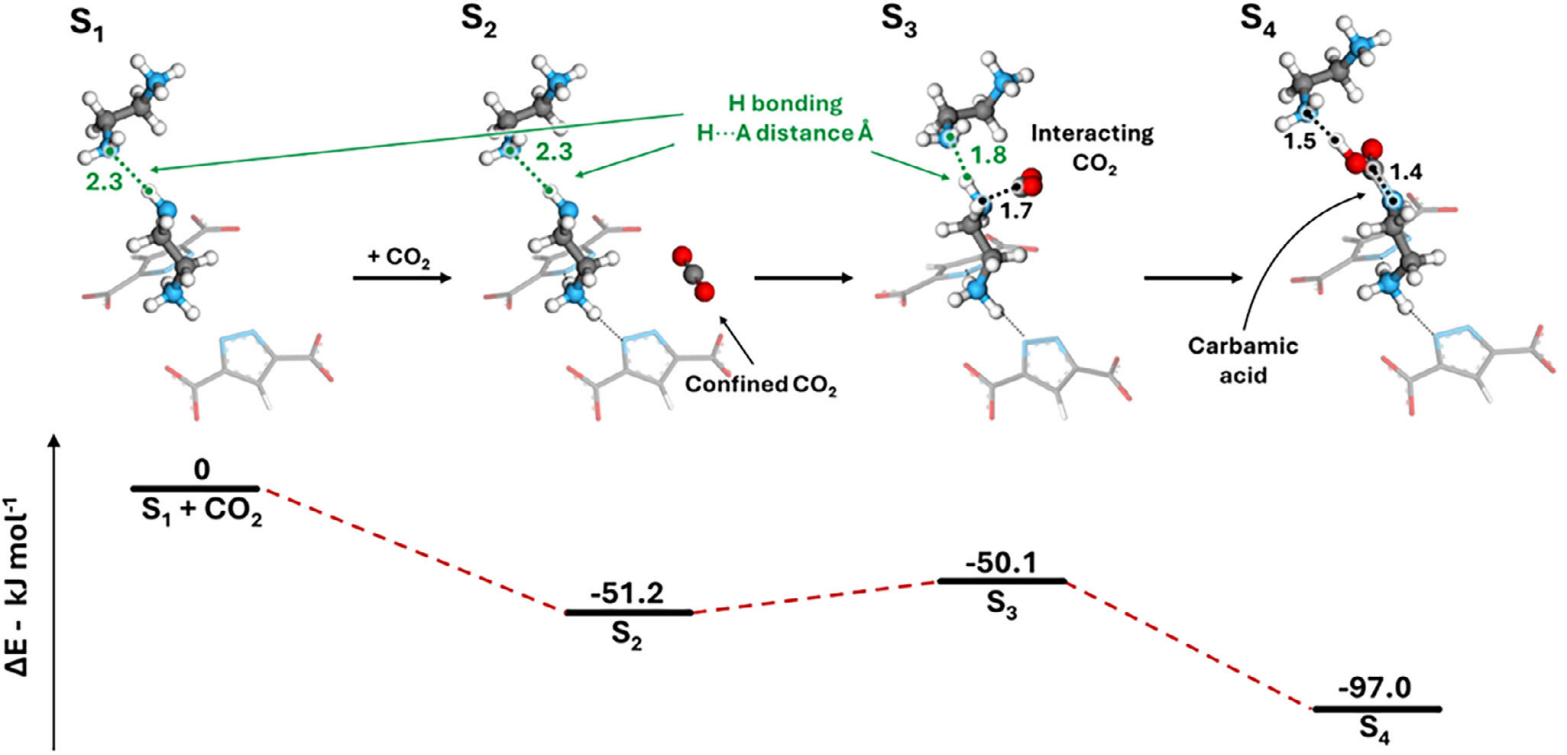
Periodic‐DFT optimized structures and relative electronic energies with respect to the reactants (structure **S_1_** and two CO_2_ molecules) for the formation of carbamates inside the MOF‐303#EDA system. Energy values are reported in kJ mol^−1^.

To further investigate the interaction between EDA and CO_2_ in the MOF pores and a plausible mechanism for carbamate formation, a second set of periodic‐DFT calculations was performed. As reported in the literature, the presence of a Brønsted base is essential to catalytically assist carbamate formation; in the case of EDA, this role can be fulfilled either by the second intramolecular amine or by the amine group from a neighboring EDA molecule [44, 45, 46]. In the global minimum MOF‐303#EDA system (**S_0_**), the free amine moieties (not grafted to the framework) are not in sufficiently close proximity to enable carbamate formation. This observation prompted an exploration of the EDA configurational space within the MOF‐303 channels. Due to the conformational flexibility of EDA, which can adopt *+gauche*, *‐gauche*, and *trans* conformations, a local minimum was identified (**S_1_**), which exhibits a favorable proximity between free amines. The **S_1_** structure was characterized by a change in the EDA‐2 conformation, from *‐gauche* to *+gauche*, thus allowing for hydrogen bonding between pairs of EDA‐2 molecules within the same channel (Figure S38). Figure 5 shows the different steps of CO_2_ adsorption into the periodic MOF‐303#EDA structure. EDA‐1 appears to form a strong hydrogen bond with the framework hydroxyl groups, which likely hinders its interaction with CO_2_ or the other amines (Figure S11). For the sake of clarity, the presence of EDA‐1 in Figure 5 was omitted. Starting from the **S_1_** structure, two CO_2_ molecules were inserted into the unit cell, and the optimized structure (named **S_2_**) showed the main sorption site for physisorbed CO_2_ inside the framework. For the carbamate formation, we identified a higher energy intermediate structure, denoted as **S_3_**, which exhibited the distorted CO_2_ molecule near the pair of hydrogen‐bonded amines of EDA‐2. One of the amines forms an N‐C interaction with the CO_2_ carbon, facilitating nucleophilic attack, while the second amine acts as a Brønsted base, serving as a hydrogen‐bond acceptor to promote proton transfer and thereby catalyze the overall reaction. The final step involves the formation of carbamate/ammonium followed by the spontaneous rearrangement to carbamic acid, as shown in structure **S_4_**, which is exothermic by 97 kJ mol^−^^1^, as shown in Figure 5. This step is energetically favorable, further supporting the formation of these species, and in agreement with both the IR and solid‐state NMR experiments. A summary of the energies and geometric parameters for the CO_2_ adsorption is presented in Table S8.

### 2.4 Dynamic Breakthrough Experiments

**FIGURE 6:**
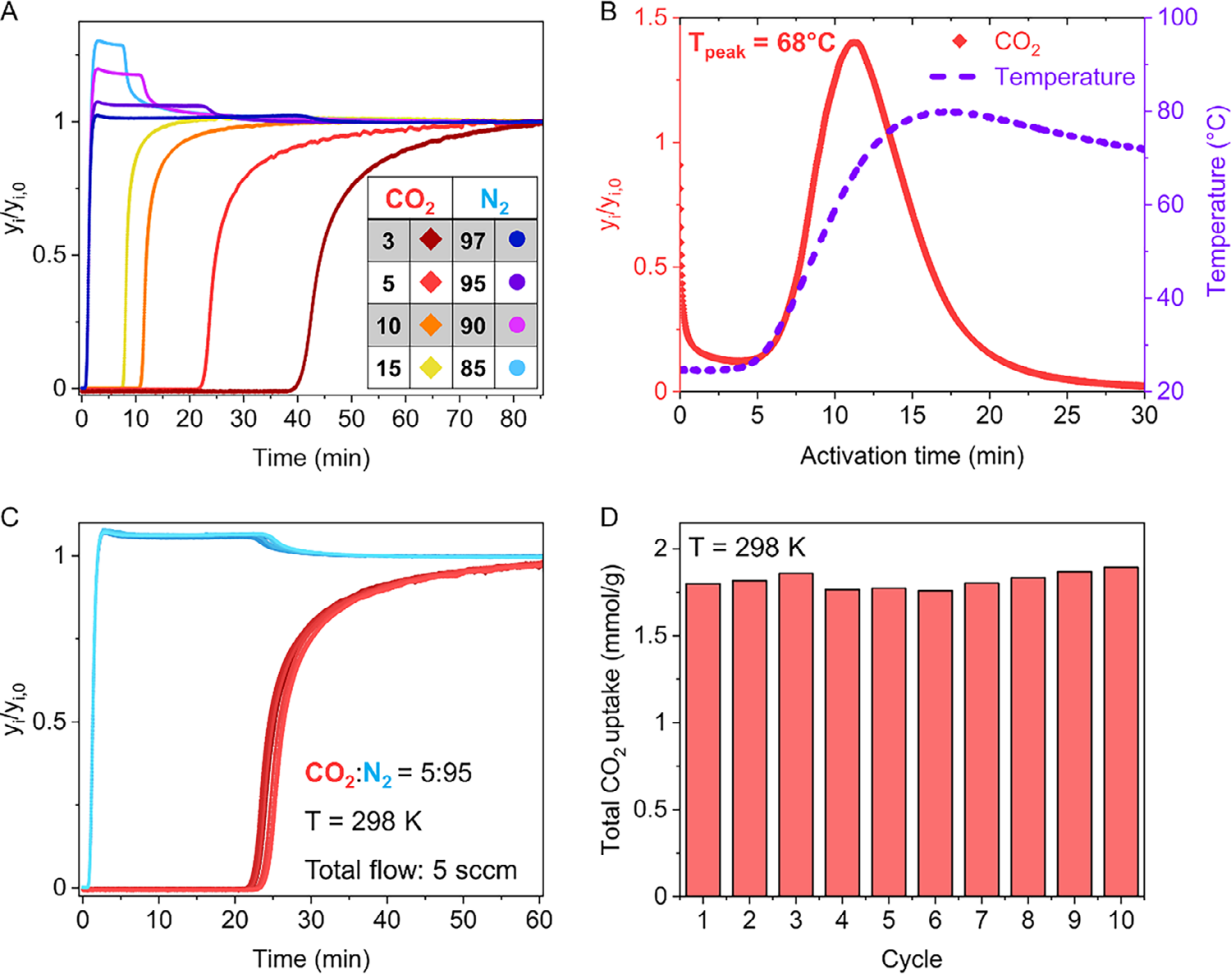
(A) Multicomponent breakthrough curves of CO_2_/N_2_ mixtures measured at 298 K with different compositions at a total pressure of 1 bar and a total flow rate of 5 sccm. The breakthrough curves were collected under the following CO_2_/N_2_ molar ratios: 3:97 (dark red diamonds/blue circles), 5:95 (orange diamonds/violet circles), 10:90 (light orange diamonds/purple circles), and 15:85 (yellow diamonds/light‐blue circles). The CO_2_/N_2_ concentrations are highlighted in the inset table. (B) CO_2_ desorption signal of MOF‐303#EDA∙CO_2_‐pellet (red curve) collected by mass spectrometry set on 44 g mol^−1^ as a function of temperature (heating rate ∼5°C min^−1^). The desorption was performed using a helium stream with a total flow rate of 10 sccm. The temperature of the packed bed was measured using a thermocouple in contact with the sorbent material (violet dashed line). (C) Ten consecutive cycling breakthrough measurements for a 5:95 CO_2_/N_2_ mixture. (D) The histogram shows the CO_2_ uptake calculated from the cycling breakthrough measurements, giving an average uptake of 1.82 ± 0.05 mmol g^−1^.

The performance of MOF‐303#EDA for carbon capture applications under industrially relevant conditions was assessed by means of multicomponent breakthrough measurements (Figure 6). The sample (176 mg) was shaped into self‐supporting pellets by compressing the amine‐loaded materials (0.5 tons) and passing the resulting material through a mesh to generate beads of controlled dimensions (diameter ∼0.5 mm). Remarkably, the compound retains its structural, textural, and sorption properties, as demonstrated by PXRD, SEM, and sorption experiments (Figures S39–S45 and Table S9).

Post‐combustion carbon capture and separation from point sources, such as CO_2_ separation from flue gases, operates within a range of 0.03 to 0.15 CO_2_ partial pressure (p_CO2_), depending on the chemical nature of the fuel employed for energy production [47, 48]. The CO_2_ capture performance was evaluated using binary mixtures of CO_2_ and N_2_ (p_CO2_ = 0.03, 0.05, 0.1, and 0.15, total pressure = 1 bar, total flow rate = 5 sccm), which simulate the composition of industrial flue gases (Figure 6A). At a 0.05 CO_2_ partial pressure and 298 K, MOF‐303#EDA shows an extremely high selectivity toward CO_2_ compared to N_2_ and an overall CO_2_ uptake of 1.80 mmol g^−1^ with a breakthrough time of 22.2 min (N_2_ purity > 99%, corresponding to 126.1 min per gram of material). Effective CO_2_ separation was also achieved for mixtures with CO_2_ partial pressures of 0.03, 0.1, and 0.15, enabling the recovery of high‐purity nitrogen (N_2_ purity > 99%) for 40.0, 10.9, and 7.2 min, respectively (Figure 6A; Figures S46 and S47). The regeneration of the compound was obtained using mild heating under continuous He flux (10 sccm). Indeed, the maximum CO_2_ desorption occurs at 68°C, as shown in Figure 6B, with the CO_2_ signal decreasing rapidly and becoming negligible after 30 min. The recyclability of MOF‐303#EDA was demonstrated by 10 consecutive breakthrough measurements (p_CO2_ = 0.05, T = 298 K, flow rate = 5 sccm), each followed by desorption experiments at 70°C for 2 h to ensure complete regeneration of the sorbent (Figure 6C; Figure S42 and S43). The mean CO_2_ uptake over 10 cycles was calculated to be 1.82 ± 0.05 mmol g^−1^, with no performance loss (Figure 6D), demonstrating that anchoring diamine moieties into MOF‐303 is a successful strategy for CO_2_ capture under operational conditions. The structural stability of MOF‐303#EDA after breakthrough experiments was evaluated with PXRD, which confirmed the preserved crystallinity after 10 breakthrough cycles (Figure S48).

## 3 Conclusions

We presented a practical and broadly applicable strategy to functionalize the pore walls of MOF‐303 with diamines, tailoring its sorption properties for efficient CO_2_ capture from air and diluted point sources. Vapor‐phase diffusion of ethylenediamine (EDA) enables a solvent‐free and selective grafting process, driven by the spontaneous deprotonation of pyrazole NH groups and stabilized by strong charge‐assisted hydrogen bonds.

The resulting MOF‐303#EDA exhibits outstanding carbon capture performance under relevant conditions, with CO_2_ uptake of 0.71 mmol g^−1^ at 450 ppm and 2.58 mmol g^−1^ at 0.15 bar (298 K). Solid‐state NMR experiments under controlled atmosphere directly reveal the formation of carbamate and carbamic acid species at the anchored amine sites, confirming the mechanistic basis of CO_2_ binding. An isosteric heat of 55 kJ mol^−1^ ensures an optimal compromise between binding strength and regenerability. Indeed, breakthrough measurements performed on shaped pellets highlight the robustness and cyclability of MOF‐303#EDA, underscoring its strong potential for practical carbon capture applications.

## Conflicts of Interest

The authors declare no conflicts of interest.

## Supporting information




**Supporting File**: smll72958‐sup‐0001‐SuppMat.pdf.

## Data Availability

All data for this article is available at Zenodo at https://doi.org/10.5281/zenodo.17543557. Some data supporting this article has been included as part of the SI (all isotherms in AIF format).
