# Supplementary material for: Diamine Grafting of Pyrazole‐Based MOF‐303 for Diluted‐Source CO2 Capture
Source: Small. 2026 Mar 3;22(23):e14197. doi: 10.1002/smll.202514197 (PMC13100560; doi:10.1002/smll.202514197)
Supplement: Supplementary file 1 — Supporting File: smll72958‐sup‐0001‐SuppMat.pdf. [file SMLL-22-e14197-s001.pdf]

Diamine grafting of pyrazole-based MOF-303 for diluted-source CO_2 capture

Giuseppe Mastronardi[a], Jacopo Perego[b], Charl Xavier Bezuidenhout[b], Wim Temmerman[c], Veronique Van Speybroeck[c], Valentina Crocellà[a], Silvia Bracco[b], Nello Li Pira[d], Angiolina Comotti*[b], Silvia Bordiga*[a].

[a] G. Mastronardi, Prof. V. Crocellà, Prof. S. Bordiga
Dipartimento di Chimica, Centro di Riferimento NIS,
Unità di Ricerca INSTM, Università degli Studi di Torino,
Via G. Quarello 15/A and Via P. Giuria 7, I-10125 Torino, Italy.
E-mail: silvia.bordiga@unito.it

[b] Dr. J. Perego, Dr. C. X. Bezuidenhout, Prof. S. Bracco, Prof. A. Comotti
Dipartimento di Scienza dei Materiali
Via R. Cozzi 55, 20125 Milano, Italy
E-mail: angiolina.comotti@unimib.it

[C] W. Temmerman, Prof. V. Van Speybroeck
Center For Molecular Modelling
Technologiepark 46 9052 Zwijnaarde, Belgium

[d] Dr. N. Li Pira
Centro Ricerche Fiat
Strada Torino, 50, 10043 Orbassano (TO), Italy

Table of contents

S1. Experimental Section 3

S2. Characterization and Structure Resolution 8

S3. CO_2 capture and *in situ* spectroscopy 23

S4. Pellet preparation and breakthrough measurements 37

Appendix 44

References 58

S1. Experimental Section

Materials

Synthesis of MOF-303

Pristine MOF-303 has been synthesized under reflux conditions using water as the solvent, following the detailed procedure described by Zheng et al.[1] Briefly, 1.74 g (5 mmol) of 3,5-Pyrazoledicarboxylic acid monohydrate ($H_2PZDC{\cdot}H_2O$) and 1.2 g (30 mmol) of NaOH were dissolved in 50 mL of deionized water in a 250 mL round-bottom flask. A solution of 2.41 g (30 mmol) of $AlCl_3{\cdot}6H_2O$ in 50 mL of water was prepared and added dropwise to the reaction vessel. The reaction mixture was heated at 120°C for 2 hours. After cooling, the reaction mixture has been centrifuged, and the precipitate has been washed 3 times with deionized water and 3 times with anhydrous ethanol. Lastly, the solid phase was dried in a vacuum oven at 80°C for 2 h to obtain MOF-303.

Synthesis of the MOF-303#EDA series

MOF-303#EDA was prepared using the following procedure. Around 0.110 g of the as-synthetized MOF-303 was degassed for 16 h at 150°C under vacuum at 3 µbar, producing approximately 0.080 g of activated MOF-303. An excess of alkyldiamine (EDA) corresponding to a 3:2 stoichiometry between EDA and the pyrazole units was used. Two separated glass vials loaded with activated MOF-303 and excess EDA were put into a container equipped with a vacuum/gas inlet. The container was frozen at 77K and then a 10^{-3} bar vacuum was applied. The freeze-and-thaw procedure was repeated three times to degas the system. The sealed container was heated to room temperature and left to equilibrate for 72 hours to allow EDA diffusion into the MOF from the vapor phase, producing the MOF-303#FullEDA. MOF-303#FullEDA was kept under an inert atmosphere and heated at 60 °C for 24 h under vacuum ($p < 5$ µbar) to remove excess amine, yielding the MOF-303#EDA.

Methods

Thermogravimetric analysis (TGA)

Thermogravimetric analyses were performed using a Mettler Toledo Star System 1, equipped with a gas controller GC10. TGA analysis of MOF-303, MOF-303#FullEDA, and MOF-303#EDA were performed under an inert atmosphere (N_2 flow, 50 mL/min). MOF-303 was outgassed overnight at 150°C under high vacuum (p =3 µbar) before TGA measurements, whereas MOF-303#FullEDA and MOF-303#EDA were inserted directly into a 70 µL alumina pan in air without any degassing procedure. The experiments were conducted by applying a thermal ramp from 30°C to 600°C and a scan rate of 15°C/min in an inert atmosphere (N_2 flow, 50 mL/min).

TGA analysis of sample MOF-303, performed under an oxidative atmosphere (dry air, flow rate = 50 mL/min) from 30°C to 1000°C, highlighted the thermal stability of the samples and allowed the evaluation of the residue after degradation of the organic component.

N_2 and CO_2 adsorption measurements

N_2 and CO_2 adsorption isotherms were collected using a Micromeritics 3Flex sorption analyzer. Nitrogen adsorption isotherms were measured at 77 K to evaluate the specific surface area (SSA). CO_2 adsorption isotherms were measured at 288 K, 298 K, 308 K, and 318 K. Prior to analysis, approximately 175 mg of MOF-303 were weighed and activated under high vacuum at 150°C (heating rate: 3°C/min) overnight. The MOF-303#EDA (~60 mg) and MOF-303#EDA-pellet (~85 mg), which had not been exposed to air before the analysis, were pre-treated under high vacuum at room temperature overnight. Specific surface areas (SSAs) were

calculated using the Brunauer–Emmett–Teller (BET) method, in accordance with the Rouquerol consistency criteria. The Langmuir equation was also employed to derive SSA.[2–4] Pore size distribution was determined using the NLDFT theory and the carbon slit pore model. The N_2 adsorption isotherm was analyzed according to the α_S method. The isotherm is plotted in a reduced form $(n/n_x)_s$ versus the relative pressure p/p_0, where the normalising factor n_x is taken as the amount adsorbed at a preselected relative pressure (p/p_0 = 0.4). The α_S plot shows the amount adsorbed as a function of the reduced standard isotherm, $\alpha_S = (n/n_x)_s$. The micropore capacity is obtained by back-extrapolation of the linear section of the α_S plot. The isosteric heat of adsorption (Q_{st}) for CO_2 was evaluated using the Van't Hoff equation, after fitting the experimental adsorption isotherms with the dual-site Langmuir-Freundlich equation, and the virial method.[5] The two methods provide consistent results. Adsorption data elaborations are reported in the Appendix.

FT-IR Spectroscopy

IR spectra were recorded in transmission mode using a Bruker Vertex 70 spectrophotometer equipped with an MCT cryodetector. Spectra were acquired within the 4000–600 cm^{-1} range, with a resolution of 2 cm^{-1}. Depending on the type of experiment, either 32 or 8 scans were collected per spectrum (see below). Samples were prepared as self-supporting pellets and mounted inside a custom-made quartz cell fitted with KBr windows, allowing for in *situ* measurements.

For the in-*situ* EDA diffusion experiment into MOF-303, 8 scans per spectrum were used to achieve higher temporal resolution. Liquid EDA was introduced using a small balloon connected to a vacuum glass line, with a *freeze-pump-thaw* cycle performed prior to vapor diffusion into the cell. Spectra were recorded every 10 seconds to monitor the diffusion process. Before the functionalization with ethylenediamine, the pristine MOF-303 was degassed at 150°C under vacuum for 4 hours.

In the in-*situ* CO_2 probe experiments, 32 scans per spectrum were acquired. CO_2 was dosed in the 0.2–120 mbar pressure range via the glass line and expanded onto the MOF surface. Before the experiment, MOF-303#EDA was degassed at room temperature under vacuum for 24 hours without air exposure, while pristine MOF-303 was degassed at 150°C for 4 hours.

Synchrotron radiation PXRD experimental conditions

The crystalline powders were poured into 0.5 mm Lindeman capillaries attached to steel gas fittings. The capillaries were then attached to a manifold that can control the pressure from a vacuum up to 20 bar. Activated samples were collected in situ under vacuum and 1 bar of CO_2 gas at 293 K for XRD experiments at the ESRF ID22 beam line using a 0.35431683 Å wavelength. PXRD measurements were collected over a range of 0.0 – 32.0° with a data collection interval of 0.002°, and data collection was done over 12 detectors simultaneously. Data was collected at various spots along the capillary and multiple times at the same spot. Any scans that showed signs of degradation were excluded from the final merged and binned dataset.

Structural resolution and Rietveld Refinement.

Rietveld structural refinements of the X-ray data were performed using the TOPAS-Academic64 V6 software package.[6] For the Rietveld refinement, the geometry optimized structures were refined with the rigid-body implementation of TOPAS as well as restraint of the coordination bond lengths with a space group *P*21/*c*. The background was fitted and refined using a Chebyshev polynomial with 15 coefficients in the PXRD trace range from 1.2° to 12° 2theta with baseline shift refinement. The "Simple_Axial_Model" was used to account for the asymmetry in the peaks. The LP factor was set to 90, and the zero error was set to -0.000087142. These

parameters are a result of the XRD setup of the ID22 beamline at the ESRF. The peaks were fitted using a Pearson VII " PVII " function.

Solid State Nuclear Magnetic Resonance

^{13}C, ^{1}H and ^{15}N solid-state NMR experiments were carried out at 75.5, 300.1 and 30.4 MHz, respectively, with a Bruker Avance Neo instrument operating at a static field of 7.04 T equipped with a 4 mm double resonance MAS probe. $^{13}C\{^{1}H\}$ and ^{15}N $\{^{1}H\}$ ramped-amplitude Cross Polarization (CP)[7] experiments were performed at 295 K at a spinning speed of 12.5 kHz using a recycle delay of 5 s and contact times of 2 ms and 50 µs for ^{13}C and 8 ms for ^{15}N nuclei. The 90° pulse for the proton was 2.5 µs. Quantitative ^{13}C $\{^{1}H\}$ Single-Pulse Excitation (SPE) experiments were run 295 K and 290 K at a spinning speed of 12.5 and 11.0 kHz using a 90° pulse of 3.84 µs and a recycle delay of 60 s. The carbon signal was acquired during t_2 under proton decoupling by applying SPINAL-64 phase modulated sequence.[8] Crystalline polyethylene was taken as an external reference at 32.8 ppm from TMS. Quantitative ^{1}H SPE MAS NMR spectra were performed at 295 K at a spinning speed of 12.5 kHz using a recycle delay of 20 s. The 90° pulse for the proton was 2.4 µs. ^{1}H SPE MAS NMR spectra were also performed with a Bruker Avance III 600 MHz instrument operating at 14.1 T, using a recycle delay of 20 s. A MAS Bruker probe head was used with 2.5 mm ZrO_2 rotors spinning at 30 kHz and the 90° pulse for the proton was 2.9 µs. The ^{1}H chemical shift was referenced to adamantane. ^{13}C and ^{1}H spectra simulations were performed by the DMFIT program[9] and they were exploited to describe MOF-303, MOF-303#EDA and MOF-303#EDA-CO_2 samples. The reduction of carbamate and carbamic acid during the vacuum/thermal treatment was followed by ^{13}C CP MAS spectra using the pyrazole signal CH as an internal reference.
The preparation of the MOF-303#EDA loaded with CO_2 for the collection of CP MAS NMR spectra were carried out under anhydrous conditions, according to the following procedure. MOF-303#EDA was activated at 60°C for 24 h under vacuum (p < 5 µbar) and then packed in the zirconia 4 mm NMR rotor and transferred in an home-made apparatus for closing rotors under a controlled atmosphere. After a second vacuum treatment at RT, $^{13}CO_2$ (99 atom % ^{13}C) was introduced into the cell at the partial pressure of 800 Torr at 298 K and allowed to equilibrate for 3.5-4 h until no change was observed (up to 760 Torr) for 1 h. The rotor was then closed with a Vespel cap and transferred to the magnet where we used an ultra-dry air flow (dew point of -80°C) for the magic angle spinning.

Helium picnometry

The density of the pore walls (ρ_w) was measured using a He picnometer (Micromeritics AccuPyc) equipped with a 1 mL cell. The apparent density (ρ_a) of the materials was evaluated considering the pore-wall density and the pore volume (V_p) as determined by N_2 adsorption isotherm at 77 K using the following equation $\rho_a = 1 / (V_p + 1/\rho_w)$.

Scanning Electron Microscopy (SEM)

MOF-303, MOF-303#EDA, and MOF-303#EDA-pellet were deposited on a conductive tape. The samples were covered with a thin layer of gold (10 nm, nominal thickness) to improve their electrical conductivity. Scanning electron microscopy (SEM) images were collected using a Zeiss Gemini 500 microscope, operating at 5 kV.

Computational details

The binding energies of the amines in MOF-303 was calculated with the code CRYSTAL23[10], using the PBE-D3(BJ) functional[11] and triple-ζ basis set[12]. Dispersion interactions were accounted for with Grimme’s D3 method[13].

Structure $\mathbf{S_0}$ (introduced in the main text) was firstly optimized in the highest symmetry group ($P2_1/c$) to reduce computational cost and then re-optimized in P1 space-group for the energetic evaluations below. This approach enabled the evaluation of the two distinct types of amine adsorption sites; each associated with the pair of pyrazole groups. The adsorption energy of both EDA-1 and EDA-2 were calculated for the following chemical reaction:

$$(\mathbf{S_0}\text{-1EDA-X}) + \text{EDA(g)} \rightarrow \mathbf{S_0}$$

By the removal of 1 EDA molecule (and the restoration of the protonated pyrazole moiety) followed by a geometry optimization of the whole structure in P1 space group and named ($\mathbf{S_0}$-1EDA-X) where X identify the type of amine removed. The adsorption energy has been calculated using the following formula:

$$\Delta E^{ads} = E[\mathbf{S_0}] - E[(\mathbf{S_0}\text{-1EDA-X})] - E[\text{EDA}]$$

This procedure was applied for both EDA-1 and EDA-2 type of amine.
To model the interactions between adjacent EDA-2 moieties within the MOF-303 framework while maintaining computational efficiency (structure $\mathbf{S_1}$, $\mathbf{S_2}$, $\mathbf{S_3}$, $\mathbf{S_4}$), the structures were optimized in the CS-2 space group.
The binding energies for the CO_2 adsorption have been calculated for the following reaction:

$$\mathbf{S_1} + 2\cdot CO_2\ (g) \rightarrow \mathbf{S_X}$$

The relative energies were calculated in reference to the reactants ($\mathbf{S_1}$ + CO_2) adopting the following equation:

$$\Delta E^{tot} = E[\mathbf{S_X}] - E[\mathbf{S_1}] - 2\cdot E[CO_2]$$

Where $\mathbf{S_X}$ is represented by one of the structures that contain CO_2 inside ($\mathbf{S_2}$, $\mathbf{S_3}$ or $\mathbf{S_4}$).
The structures $\mathbf{S_2}$, $\mathbf{S_3}$ and $\mathbf{S_4}$ contain CO_2 either in molecular or carbamic acid form, it is important to remember that because of the symmetry adopted, we need to consider the insertion of 2 molecules of CO_2 inside the crystal cell; for the sake of clarity the relative energies shown herein and in the main text were considered per single molecule of CO_2 adsorbed, dividing the previous calculated ΔE^{tot} by two:

$$\Delta E = (E[\mathbf{S_X}] - E[\mathbf{S_0}] - 2\cdot E[CO_2])/2$$

The basis set superposition error (BSSE) associated with CO_2 interaction with structure S_2 and S_3 was evaluated using the Boys–Bernardi counterpoise correction (CPC) on the optimized geometries.[14] the corrected binding energies were estimated using the following equation:

$$BSSE=[E^{AB}(A)-E^{A}(A)] + [E^{AB}(B)-E^{B}(B)]$$

And then added to the uncorrected ΔE.

$$\Delta E(CPC) = \Delta E + BSSE$$

NMR chemical shielding of the periodic structural models was calculated using the DFT-GIPAW method[15,16] implemented in VASP6[17]. For the NMR calculations, the PBE-D3(BJ) functional was used with an energy cutoff for plane waves of 1200 eV.

Before chemical shielding calculation, the initial structures (**S_0** and MOF-303) were re-optimized with VASP6 *via* iterative alternation between full and fixed cell-parameter optimizations until complete convergence, effectively removing Pulay stress contributions. [18,19]

Sampling of the Brillouin zone in both sets of calculations (both CRYSTAL23 and VASP6) was limited to the Γ point.

Breakthrough experiments

Breakthrough experimental setup and sample preparation

Breakthrough experiments were performed using gases with a purity of 5.0 (N_2 and He) and 4.5 (CO_2). The breakthrough curves were collected using a Micromeritics SAA (Selective Adsorption Analyzer) coupled with a mass spectrometer (Pfeiffer Vacuum ThermoStar GSD 301 T3 Benchtop Mass Spectrometer) placed at the exit of the breakthrough column. The mass spectra signals have been recorded against time. The opening times of the valve, the temperature of the sample and the environmental chamber and the flow rates were recorded and stored.[20–22] The sample, MOF-303#EDA-pellet (176 mg), was packed in a ¼ inch stainless-steel column and activated *in situ* under continuous He flow (10 sccm) at 353 K for 2 hours before breakthrough measurements.

Dead volume and MS response correction

The measured breakthrough curves were analyzed and corrected for the system dead volume and the detector (MS) response. The blank curves were collected by filling the breakthrough column with glass beads of ~ 1 mm. The volume of glass beads was the same as the volume occupied by the sample. It was evaluated by measuring the density of the glass using He pycnometry and weighing the correct amount of inert material inside the column (see Table A12 in the Appendix). Thus, the dead volume of the blank correction is equal to the empty volume in the presence of the sample. Breakthrough experiments with the dense glass beads were conducted using the same parameters, such as the total flow rate, temperature, CO_2/N_2 mixture compositions, as the breakthrough measurements performed with the porous sample, and the measured dead time was subtracted from the raw data. Typical values for the dead time correction were ~ 1 minute for a total flow rate of 5 sccm, while the CO_2 breakthrough times in the presence of the adsorbent phase were about 22 minutes under the same conditions (CO_2:N_2 = 5:95).

S2. Characterization and Structure Resolution

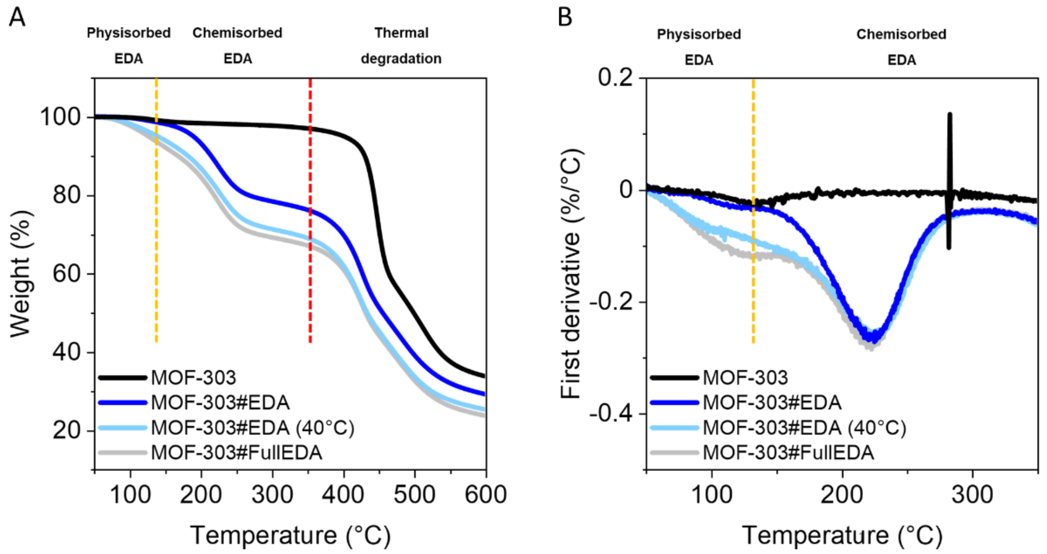

Figure S1. A) Thermogravimetric analysis of activated MOF-303 (black line), MOF-303#FullEDA (light-grey line), MOF-303#FullEDA after thermal treatment at 40°C under dynamic vacuum (light blue line), and MOF-303#EDA (blue) under N_2 flux (50 mL/min) from 50°C to 600°C. The temperature ranges corresponding to the release of physi- and chemi-sorbed EDA and the thermal degradation of the framework are delimited using vertical yellow and red dotted lines, respectively. B) First derivative of the thermogram between 50°C and 350°C. The release of the physi- and chemisorbed EDA molecules is associated with two distinct phenomena.

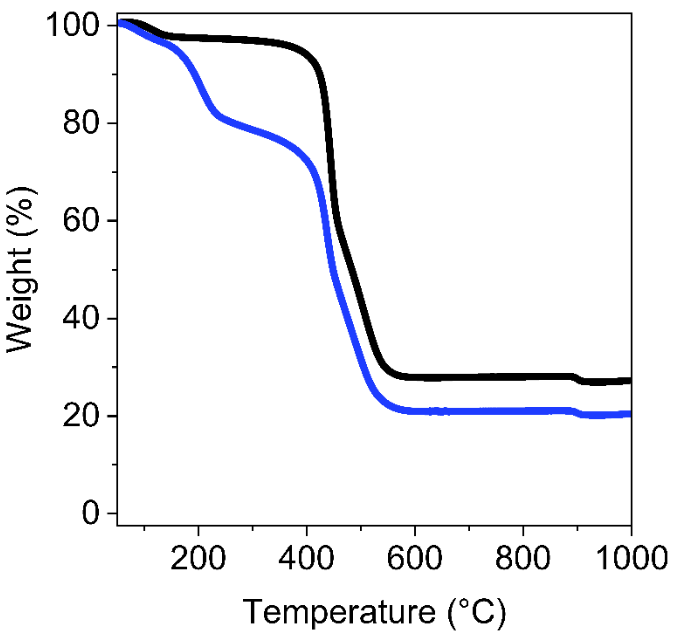

Figure S2 Thermogravimetric analysis of sample MOF-303 (black line) and MOF-303#EDA (blue line) measured under oxidative atmosphere (dry air, 50 mL/min). The experimental residue of MOF-303 at 950°C is 26.8 % in good agreement with the theoretical residue calculated from the formula Al(OH)(PZDC) (25.7 %).

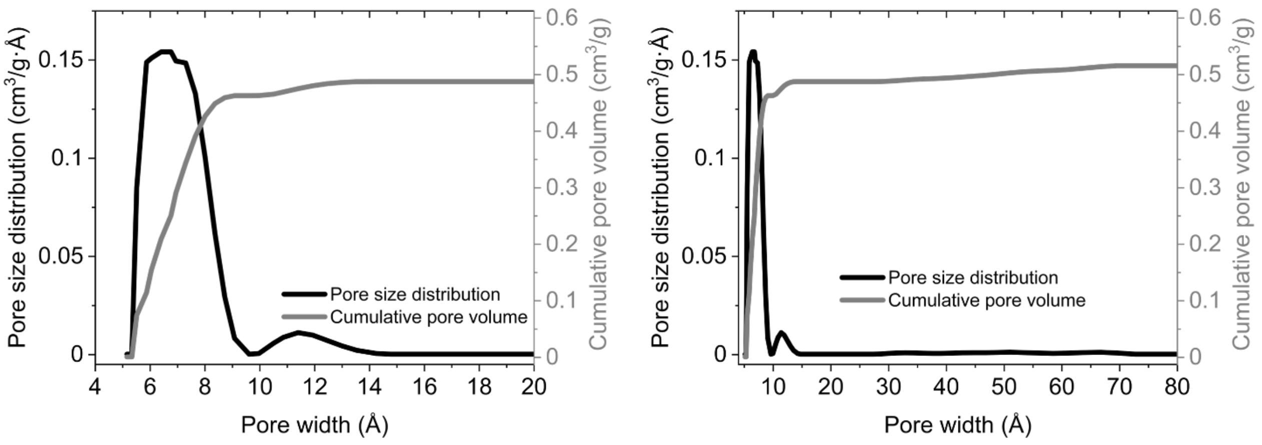

Figure S3 Pore size distribution (PSD, black line) and cumulative pore volume (CPV, grey line) for MOF-303 calculated between 4 and 20 Å (left) and between 4 and 80 Å (right). The pore size distribution is centered at ~7 Å. The pore size distribution was calculated up to 0.8 p/p_0 using the NLDFT theory and the carbon slit pore model.

Table S1 Textural properties calculated for MOF-303 and MOF-303#EDA from N_2 sorption isotherm measured at 77 K.

**Sample**	**BET SSA(m^2/g)[a]**	**Langmuir SSA (m^2/g)**	**Micropores volume (NLDFT, cm^3/g)[b]**	**Micropores volume (α_S plot, cm^3/g)[c]**
MOF-303	1417 ± 1	1469 ± 2	0.52	0.50
MOF-303#EDA	45.3 ± 0.1	52 ± 0.6	0.02	-

[a] The BET specific surface area were calculated in accordance with the Rouquerol consistency criteria, as detailed in the Appendix. [b] The pore size distribution and the total pore volume were determined from the experimental N_2 adsorption isotherm measured at 77 K using the NLDFT theory and the carbon slit pore model. [c] The total pore volume was determined from the extrapolation of the linear section of the α_S plot, as detailed in the Appendix.

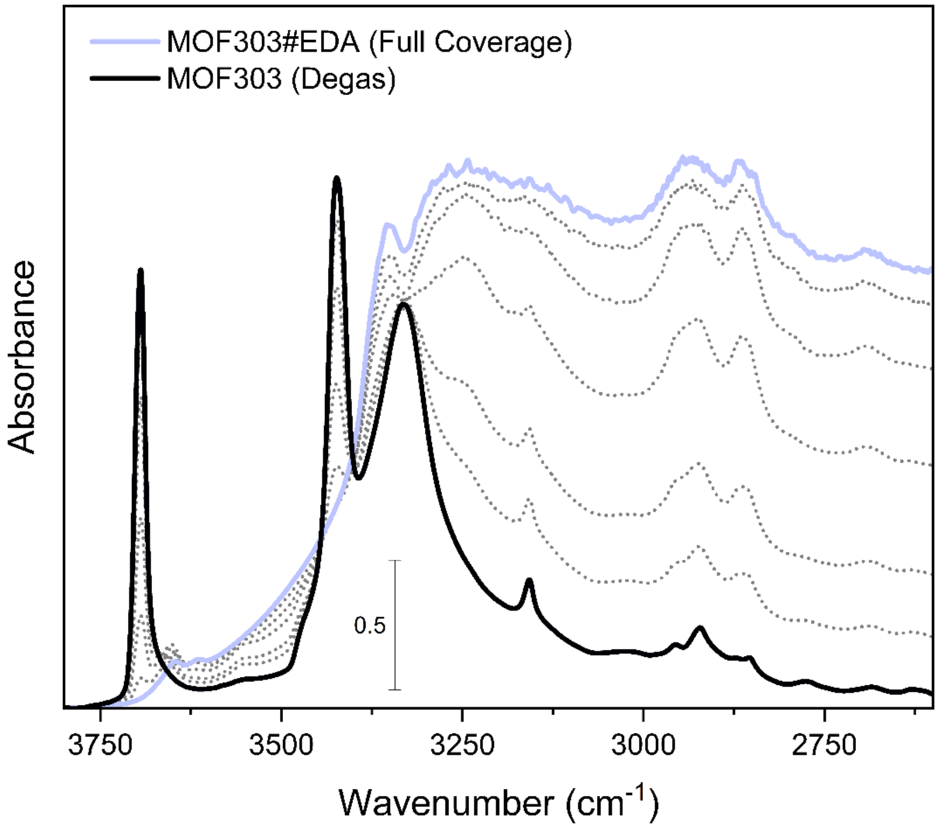

Figure S4 IR spectra collected during *in situ* diffusion of EDA vapors in MOF-303 channels. The pristine MOF-303 (black curve) has been degassed in vacuum as reported in the experimental section before EDA diffusion. EDA was carefully diffused inside the IR cell, and sequential spectra were acquired until the full coverage of the MOF-303 system was reached, when no further changes in the IR spectra were visible (light blue curve).

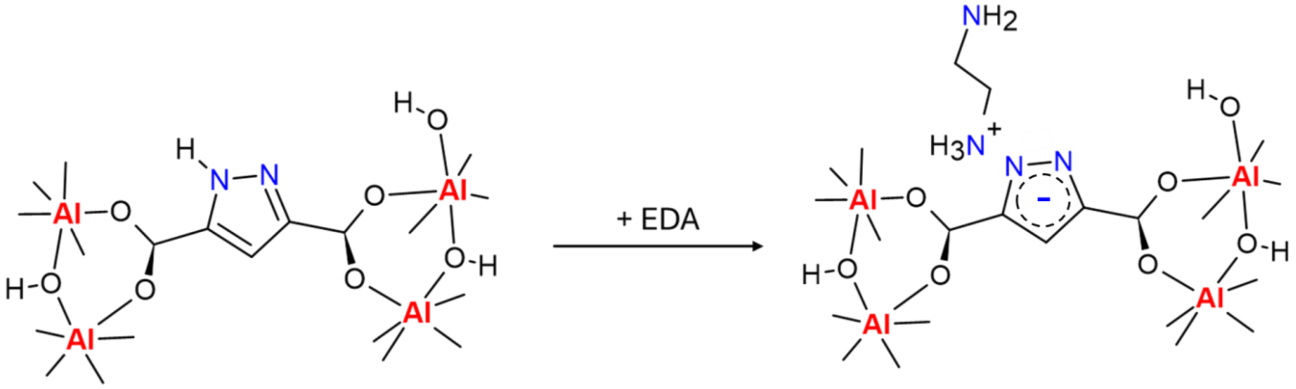

Figure S5 Scheme of proposed EDA functionalization mechanism on MOF-303 following its gas-phase diffusion.

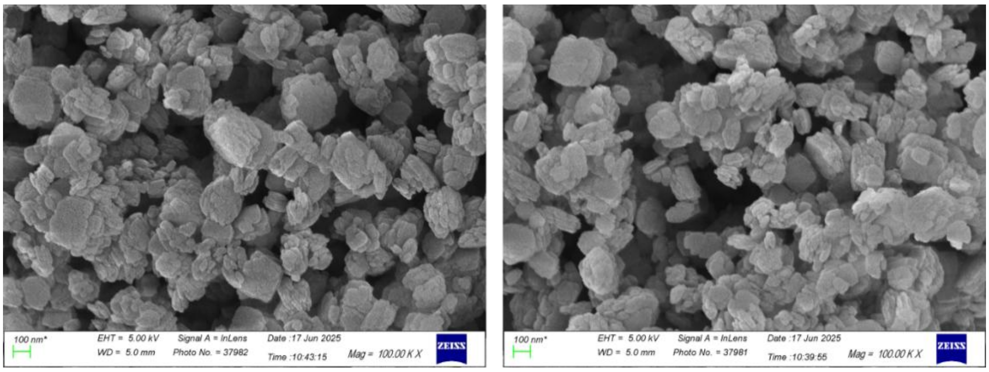

Figure S6 SEM images of MOF-303.

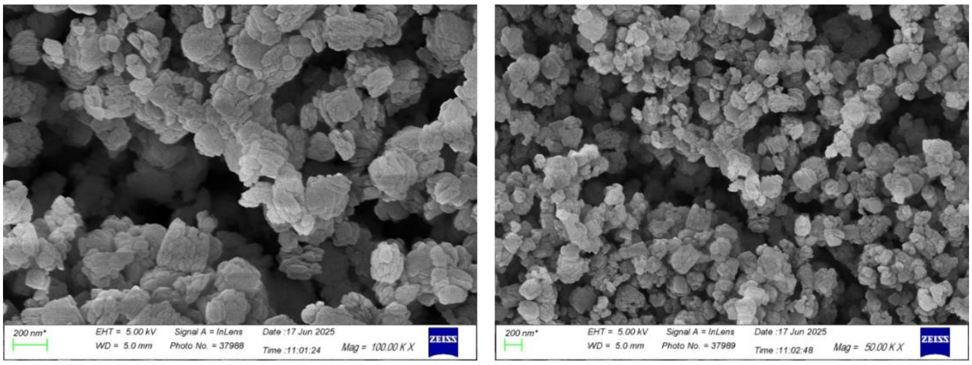

Figure S7 SEM images of MOF-303#EDA.

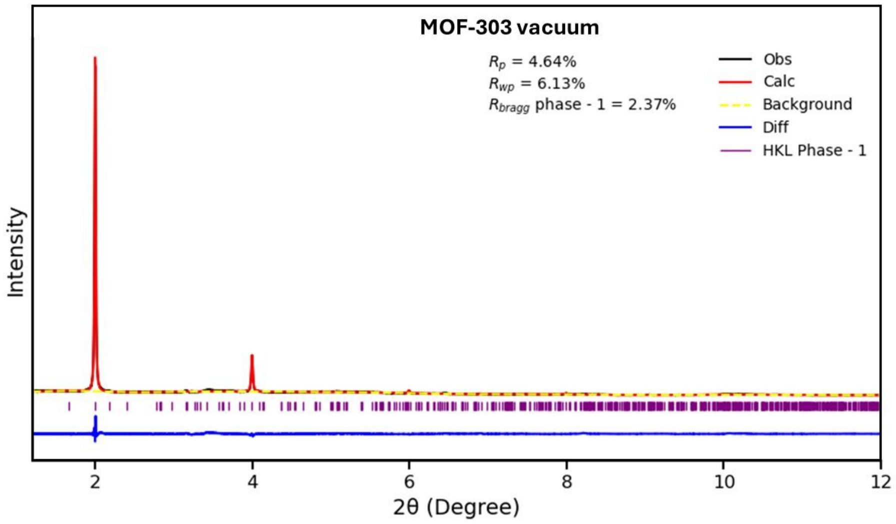

Figure S8 The Rietveld plot for the *in-situ* PXRD pattern of MOF-303 collected under dynamic vacuum at 293 K at the ESRF ID22 beamline by synchrotron radiation at 0.35432 Å.

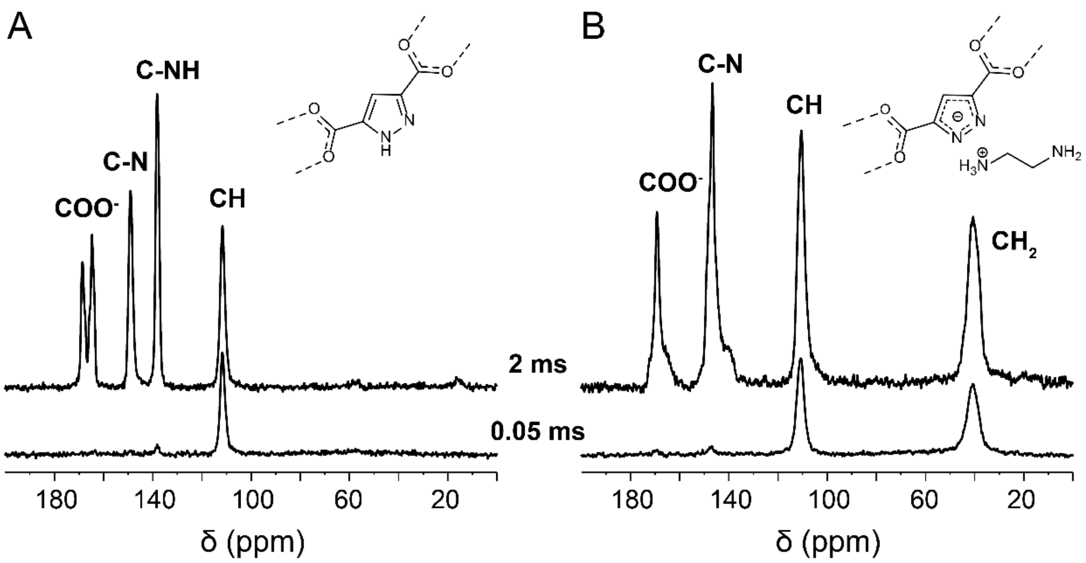

Figure S9 ^{1}H-^{13}C CP MAS NMR spectra of MOF-303 (A) and MOF-303#EDA (B) collected at 7.04 T and 295 K with a spinning speed of 12.5 kHz and a contact time of 2 ms (above) and 0.05 ms (below).

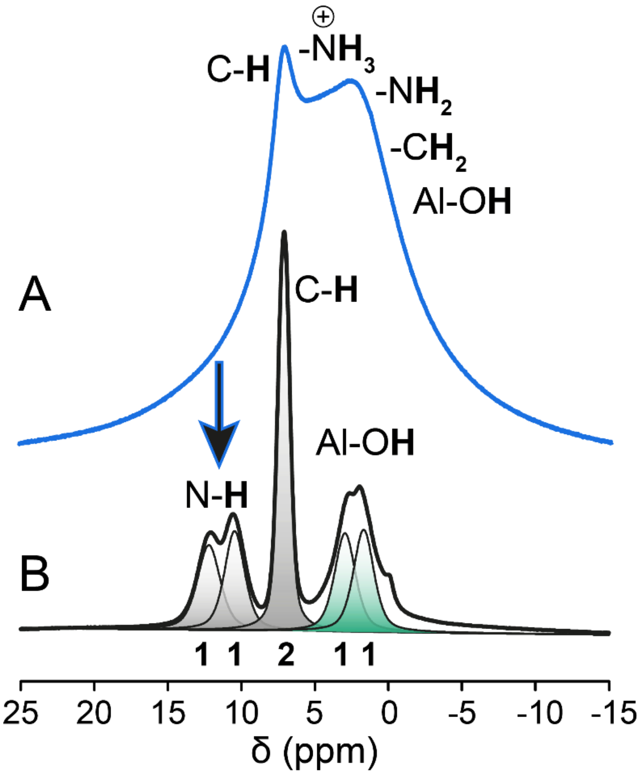

Figure S10 Quantitative ^{1}H SPE MAS NMR spectra collected at 7.04 T and 295 K with a spinning speed of 12.5 kHz and a recycle delay of 20 s of MOF-303#EDA (A) and MOF-303 (B).

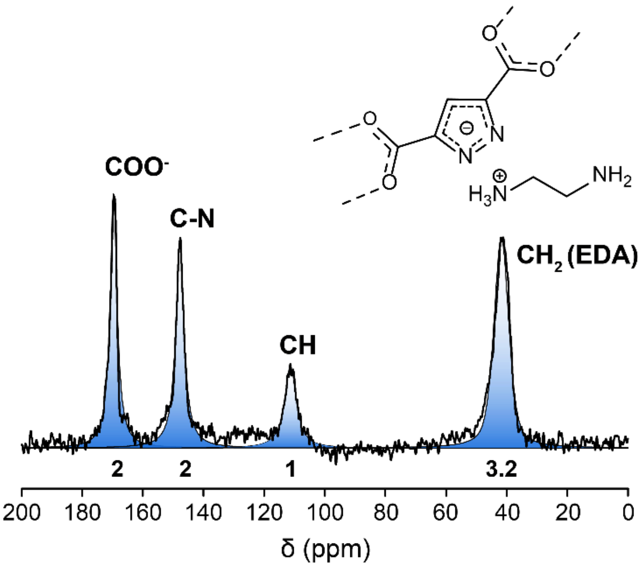

Figure S11 Quantitative ^{13}C SPE MAS NMR spectrum of MOF-303#FullEDA collected at 7.04 T and 290 K with a spinning speed of 12.5 kHz and a recycle delay of 60 s. Analysis of the peak areas unveils a 1.6:1 ratio between EDA and pyrazolate units, in agreement with the TGA analysis.

Table S2. ^{13}C chemical shifts of MOF-303 from the simulation of quantitative ^{13}C SPE MAS NMR spectrum collected with a recycle delay of 60 s. ^{1}H and ^{15}N chemical shifts from the simulation of quantitative ^{1}H SPE MAS NMR spectrum collected with a recycle delay of 20 s and ^{1}H-^{15}N CP-MAS NMR spectrum collected with a contact time of 8 ms. All experiments were performed at 7.04 T and 295 K with a spinning speed of 12.5 kHz.

**MOF-303**	**Assignment**	**^{13}C SPE MAS δ (ppm)**
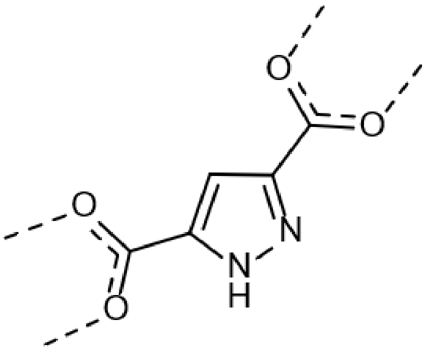	**C**OO^{-}	168.4<br>164.5
	**C**N	148.8
	**C**NH	138.0
	**C**H	111.5
	**Assignment**	**^{1}H SPE MAS δ (ppm)**

	N**H**	12.2<br>10.5
	C**H**	7.1
	Al-O**H**	2.9<br>1.7
	**Assignment**	**^{1}H-^{15}N CP MAS δ (ppm)**
	**N**	317.2<br>310.0
	**N**H	207.5<br>199.2

Table S3. ^{13}C chemical shifts of MOF-303#EDA from the simulation of ^{13}C SPE MAS NMR spectrum collected at 7.04 T and 295 K with a spinning speed of 12.5 kHz and a recycle delay of 60 s.

**MOF-303#EDA**	**Assignment**	**^{13}C SPE MAS δ (ppm)**
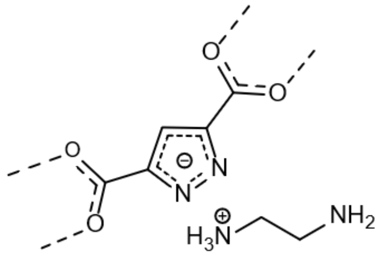	**C**OO^-	169.2
	**C**N	146.9
	**C**H	110.7
	**C**H_2	41.0

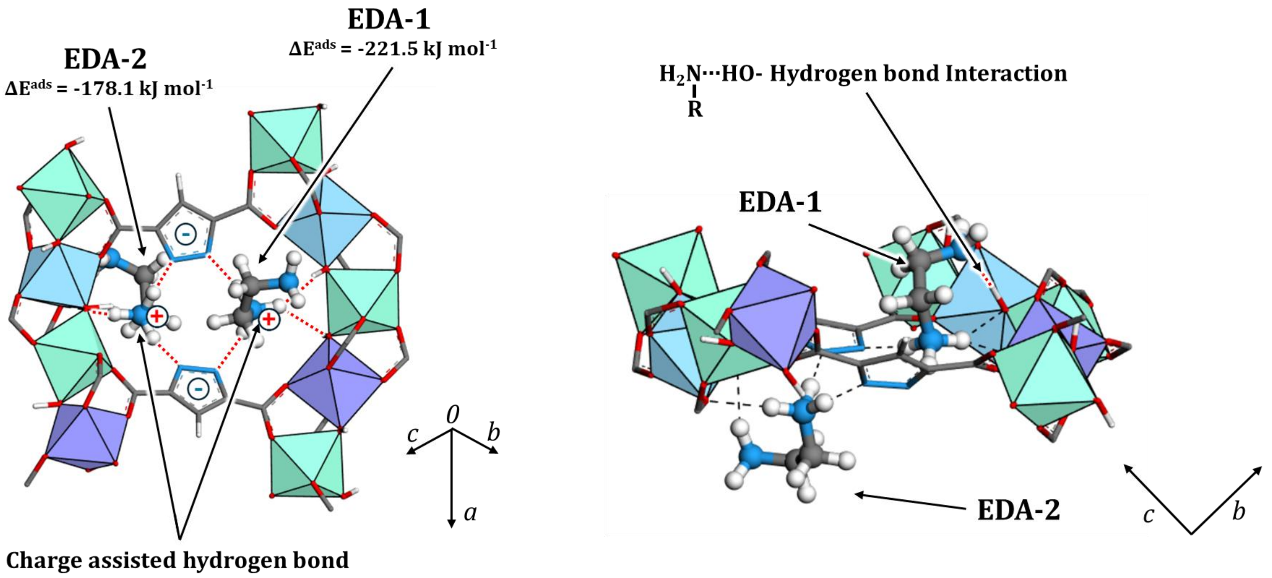

Figure S12 Fragments of the DFT-optimized S_0 structure with a particular focus on EDA-1 and EDA-2 binding site and their adsorption energies.

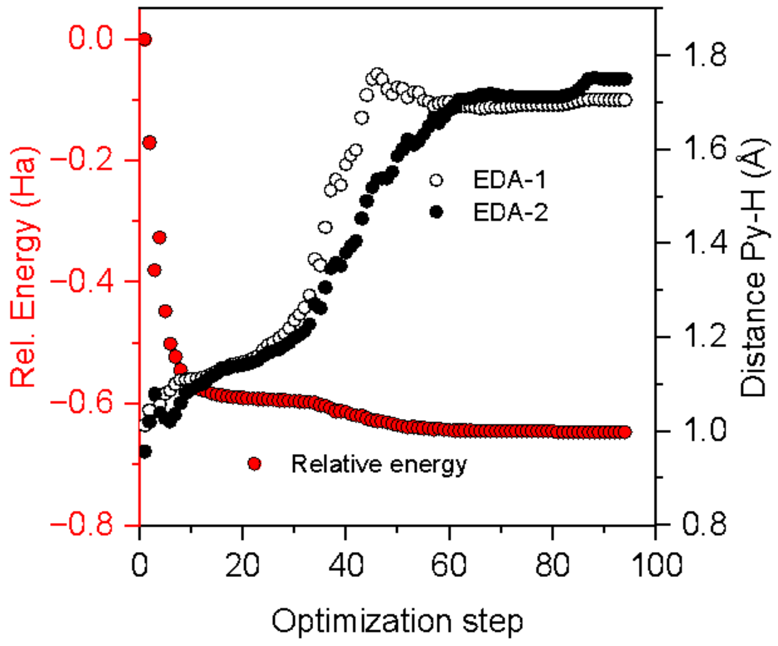

Figure S13 Relative energy and N-H distance of pyrazole moiety curve during the optimization steps of S_0 structure. Interestingly, the N-H bond breaks during the optimization step as a result of proton transfer to the amine moiety.

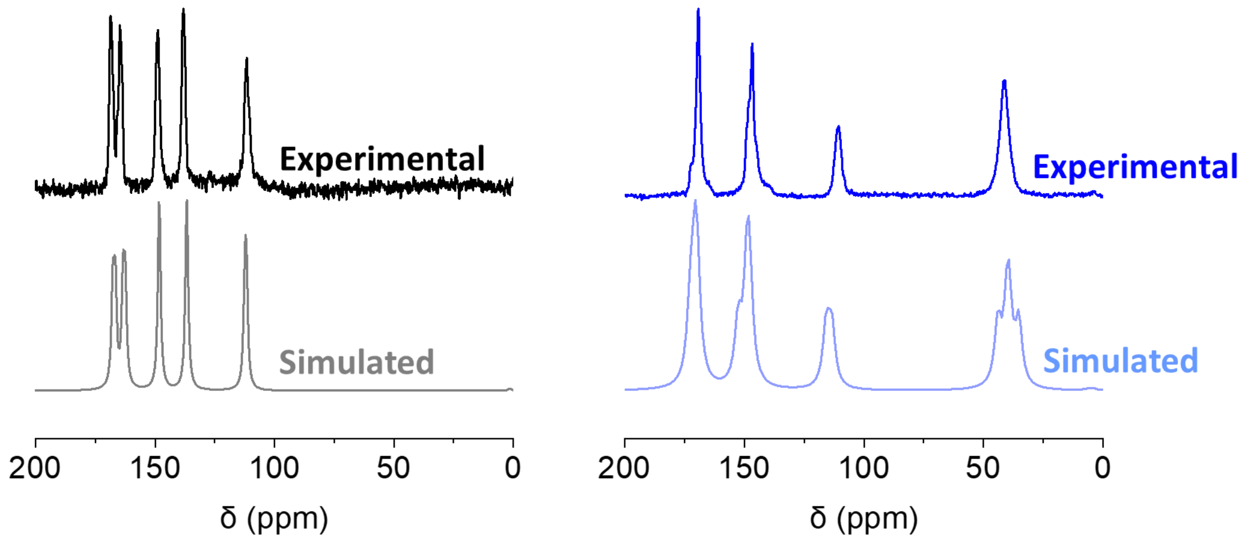

Figure S14 Experimental and simulated ^{13}C NMR spectra: comparison of MOF-303 (black and grey, respectively) and MOF-303#EDA (blue and light-blue, respectively; based on the **S₀** and DFT-optimized MOF-303 structure).

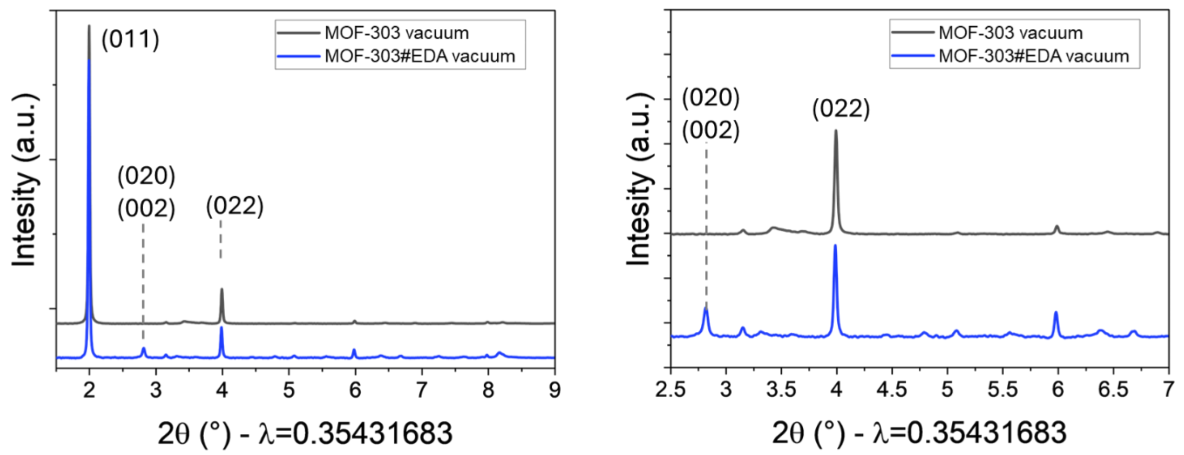

Figure S15 In-situ PXRD patterns of MOF-303 (black), and MOF-303#EDA (blue) collected under dynamic vacuum at 293 K at the ESRF ID22 beamline by synchrotron radiation at 0.35432 Å. Selected Miller indices are indicated.

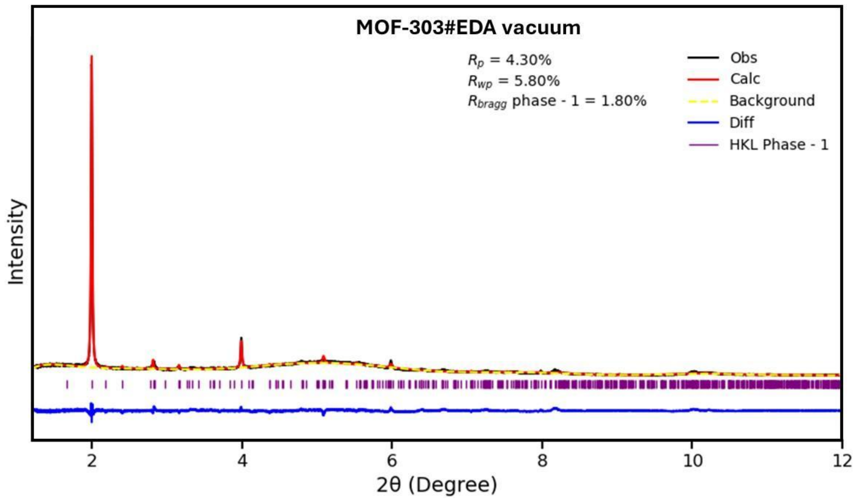

Figure S16 The Rietveld plot for the in-situ PXRD patterns of MOF-303#EDA collected at 293 K at the ESRF ID22 beamline by synchrotron radiation at 0.35432 Å.

Table S4. The refinement details and cell parameters for the Rietveld refinement of **MOF-303 vacuum** and **MOF-303#EDA vacuum** against data collected at the ESRF performed at 293 K under vacuum using synchrotron radiation.

	**MOF-303 vacuum**	**MOF-303#EDA vacuum**
Chemical Formula	C10 H6 N4 O10 Al2	C10 H6 N4 O10 Al2, 2(N2H9C2)
Formula Weight	396.1375	516.335
Z	4	4
Temperature	293 K	293 K
Space Group	*P21/C*	*P21/C*
a (Å)	13.229	11.977
b (Å)	14.292	14.274
c (Å)	14.842	14.719
β (°)	103.03	99.72
V (Å^3)	2733.9	2480.51
Rbragg (%)	2.37	1.80
Rp (%)	4.64	4.30
Rwp (%)	6.13	5.80
Refinement Method	Rietveld	Rietveld
Measurement Device Type	ESRF Synchrotron	ESRF Synchrotron
Radiation Wavelength (Å)	0.35432	0.35432
Measurement Method	Continuous Scan	Continuous Scan

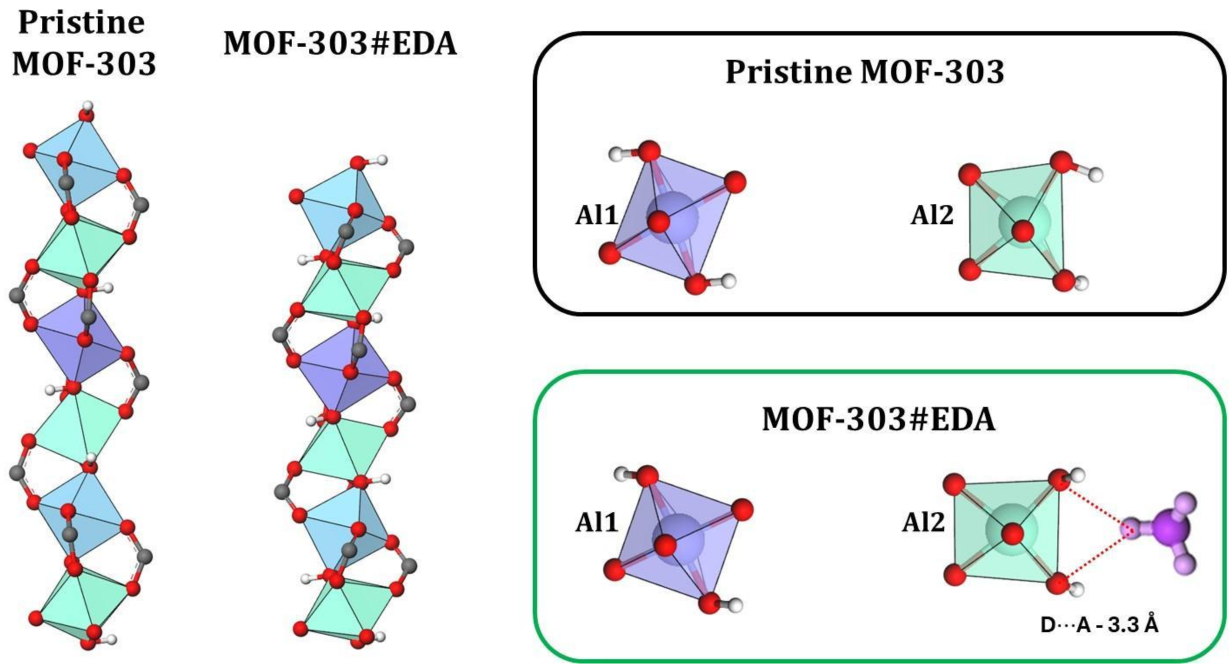

Figure S17 A comparison of the 1D $AlO_4(OH)_2$ nodes of pristine MOF-303 ($Al_2(OH)_2PZDC_2$) and MOF-303#EDA ($Al_2(OH)_2PZDC_2EDA_2$) with $AlO_4(OH)_2$ octahedra shown in different colours and the *trans* and *cis* corner-sharing octahedra highlighted. The interactions between the Al2 octahedra and the ammonium group of EDA-2 is shown with the D···A distance of 3.3 Å for both OH groups.

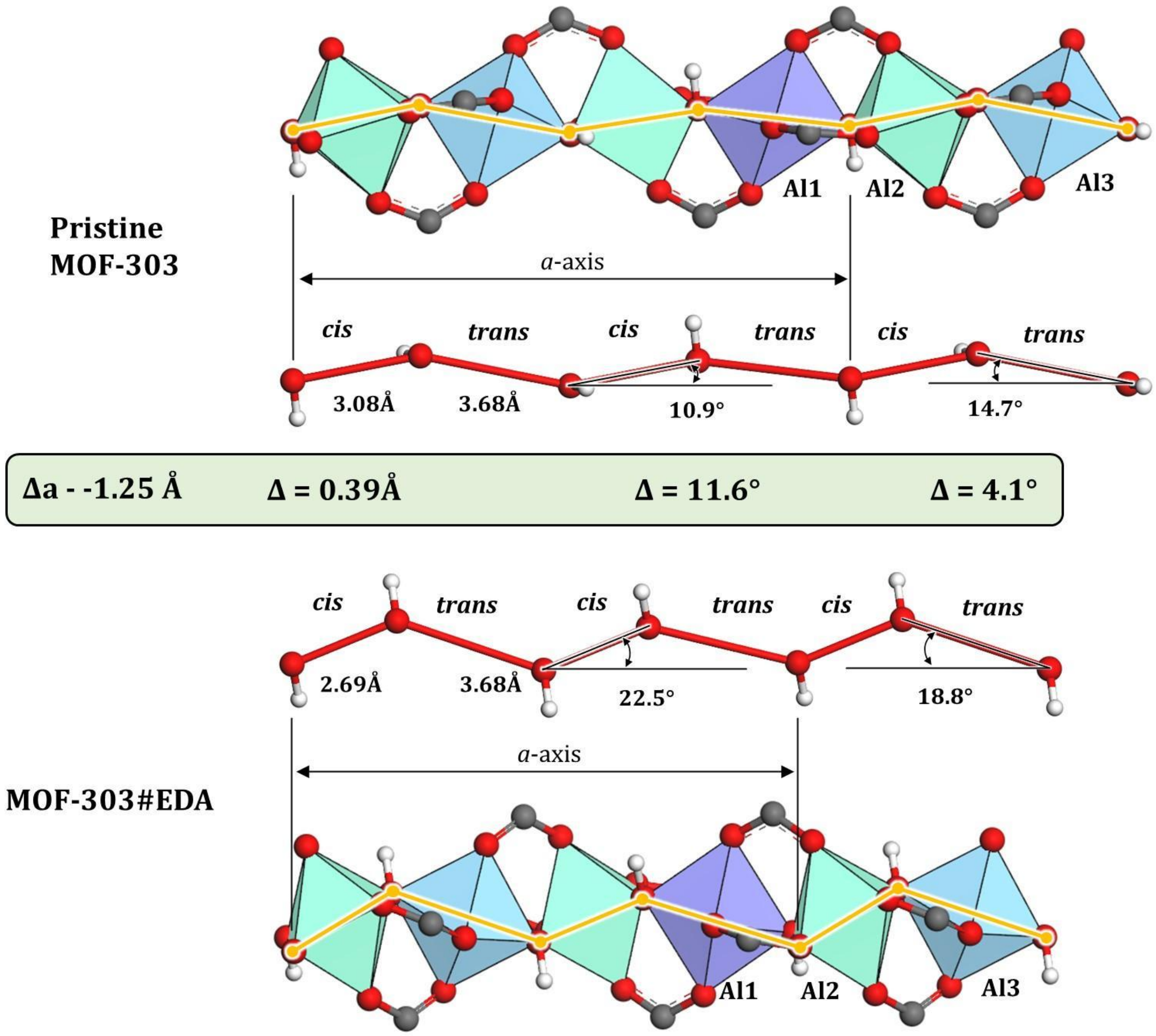

Figure S18 A comparison of the 1D $AlO_4(OH)_2$ nodes of pristine MOF-303 ($Al_2(OH)_2PZDC_2$) and MOF-303#EDA ($Al_2(OH)_2PZDC_2EDA_2$) with $AlO_4(OH)_2$ octahedra showing the corner-to-corner distances which propagate along the a-axis. The *trans* and *cis* corner-sharing octahedra are highlighted. The angles shown are the tilting angle of the octahedra with respect to the a-axis, which can result in a shrinking/stretching of the axis. The change in the a-axis and the other measurements shown are highlighted in the green rectangle.

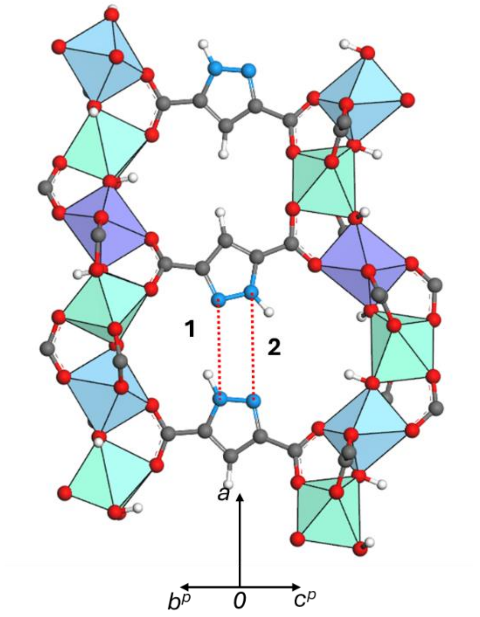

	D···A distances (Å)	D—H···A angles (°)
1	3.9	145.8
2	4.3	128.0

Figure S19 The interactions between neighbouring HPZDC ligands are shown with a red dashed line. The D···A distances and D—H···A angles are tabulated on the right.

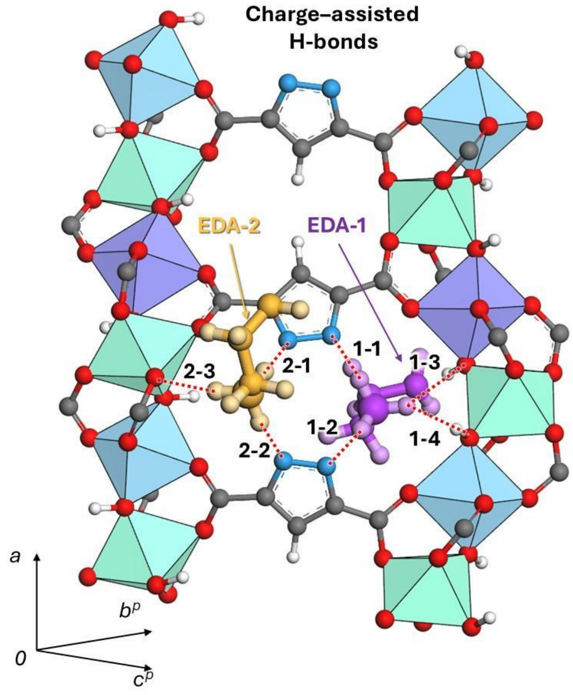

	D···A distances (Å)	D—H···A angles (°)
1-1	2.77	171.3
1-2	2.73	158.6
1-3	3.25	144.9
1-4	3.31	145.9
2-1	2.66	175.4
2-2	2.69	166.3
2-3	2.96	149.4

Figure S20 The interactions between ammonium cations of the EDA molecules and the PZDC ligands, as well as the node OH and O (COO) groups, are shown with a red dashed line. The D···A distances and D—H···A angles are tabulated on the right.

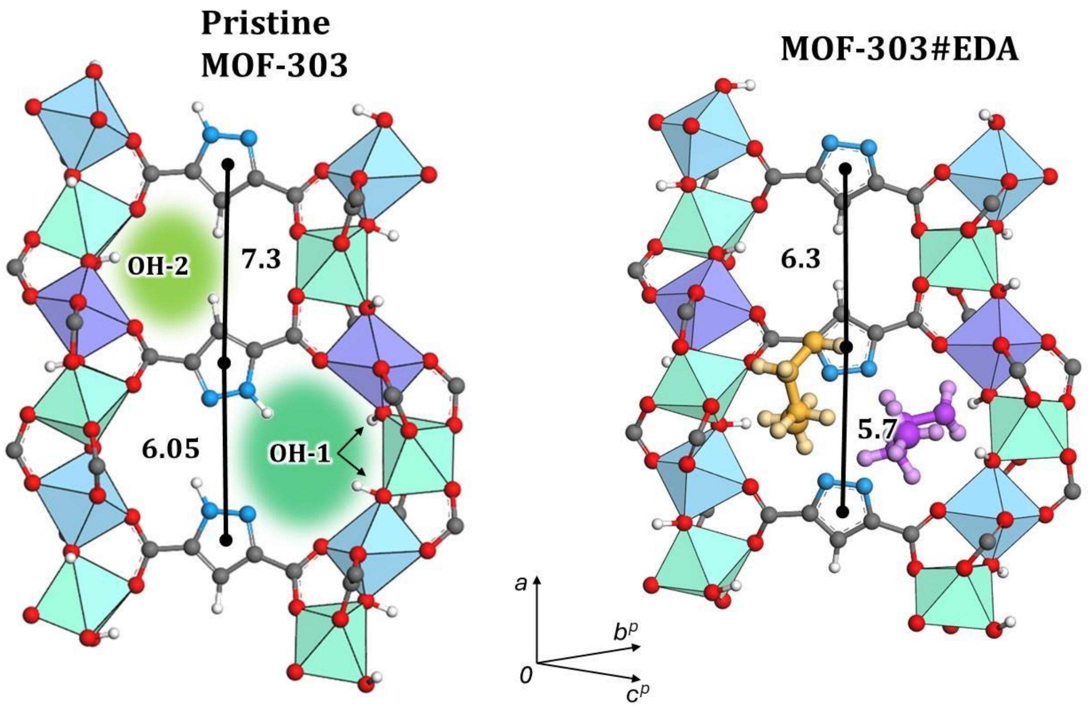

Figure S21 Centre-to-centre distance between HPZDC (MOF-303) and PZDC (MOF-303#EDA) ligands in Å. The grey and blue zones indicate the different structural environments of the two symmetry-independent OH groups of the 1D node within the crystal structure.

S3. CO_2 capture and *in situ* spectroscopy

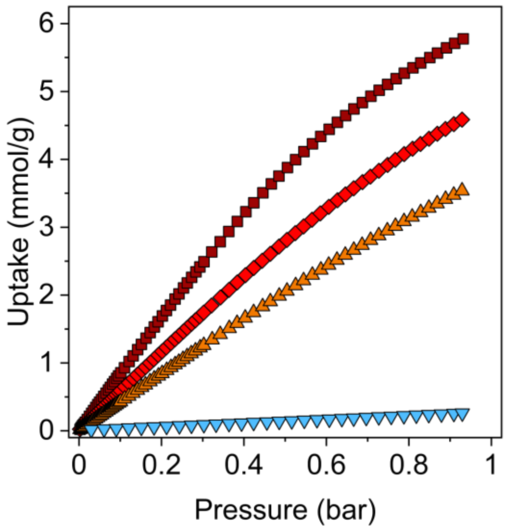

Figure S22 CO_2 adsorption isotherms of MOF-303 collected at 288 K (dark red squares), 298 K (red diamonds), and 308 K (orange triangles). N_2 adsorption isotherm collected at 298 K (down-pointing light blue triangles). Only the adsorption branches are shown for clarity.

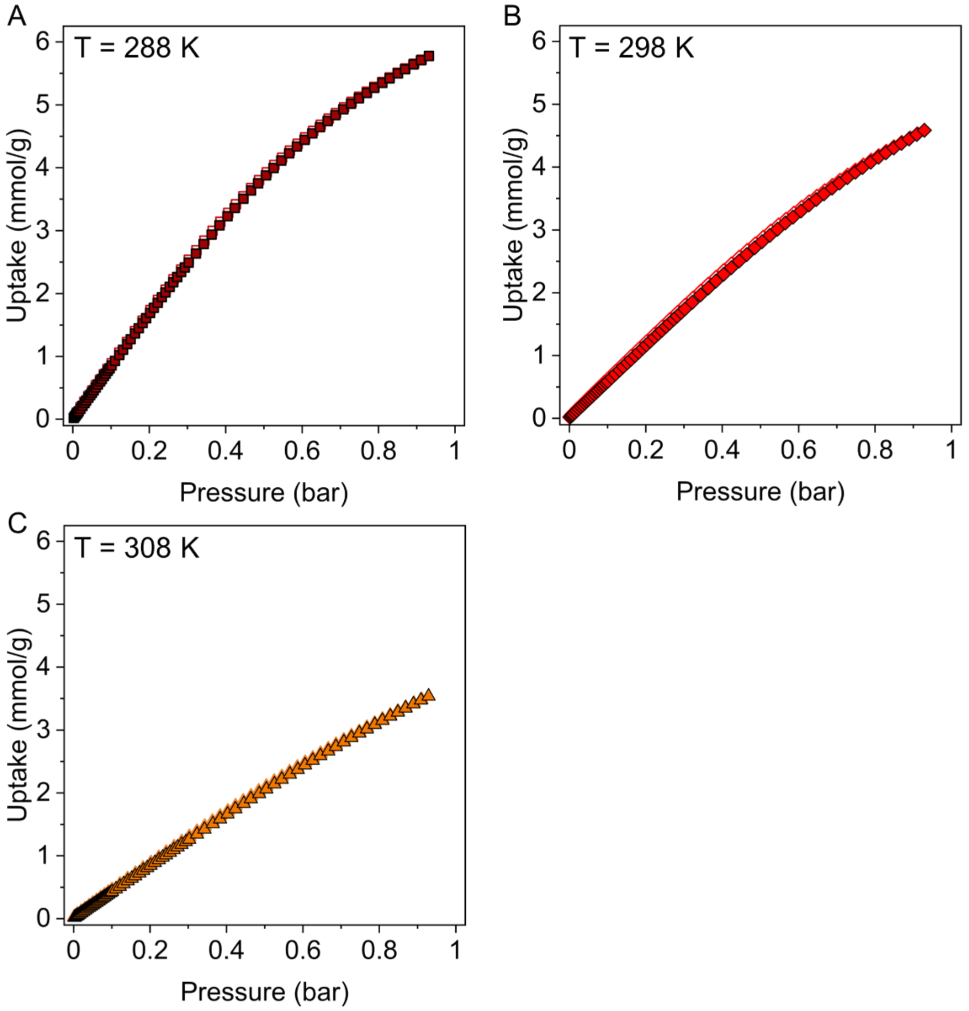

Figure S23 A) CO_2 adsorption isotherm of MOF-303 collected at 288 K (dark red squares). B) CO_2 adsorption isotherm of MOF-303 collected at 298 K (red diamonds). C) CO_2 adsorption isotherm of MOF-303 collected at 308 K (orange triangles). Filled and empty symbols represent sorption and desorption branches, respectively.

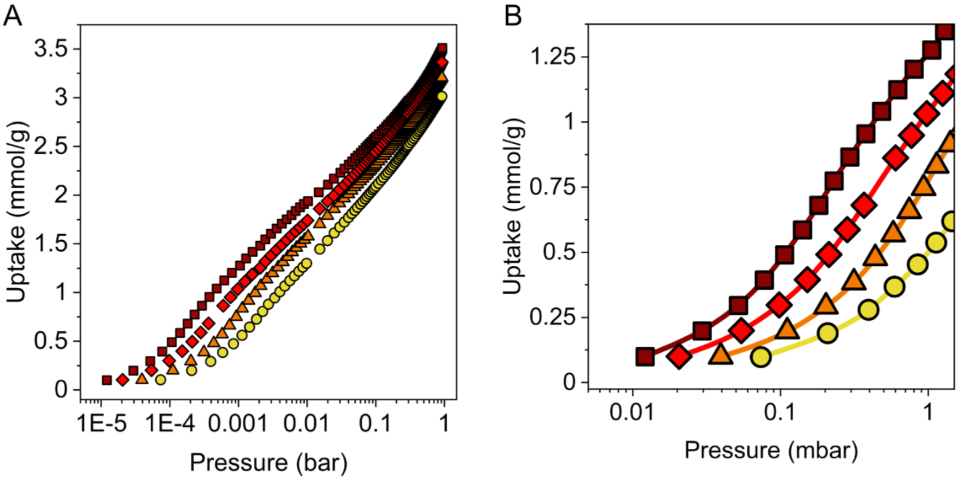

Figure S24 A) CO_2 adsorption isotherms of MOF-303#EDA measured at 288 K (dark red squares), 298 K (red diamonds), 308 K (orange triangles), and 318 K (yellow circles) in semilogarithmic scale. B) Zoomed in region between 0.005 mbar and 1.5 mbar. Only the adsorption branches are shown for clarity.

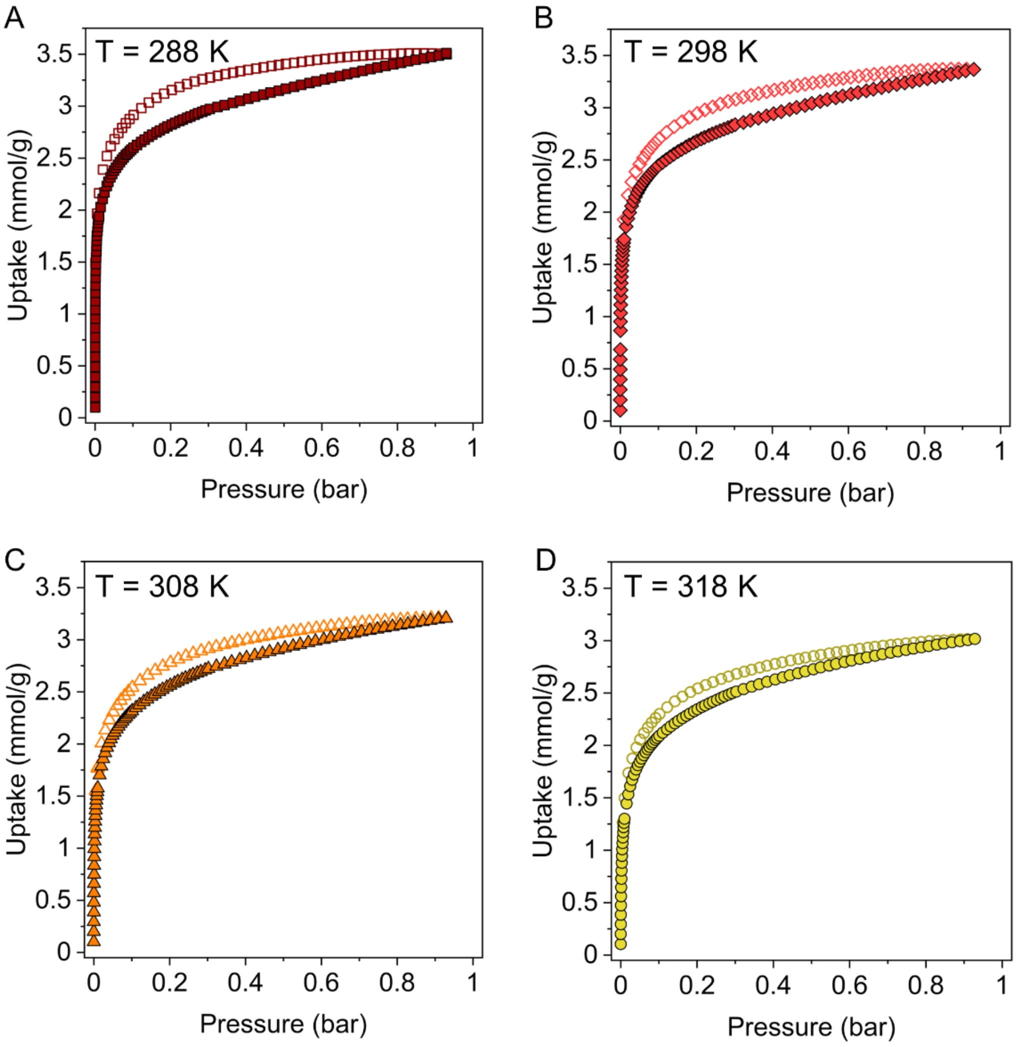

Figure S25 A) CO_2 adsorption isotherm of MOF-303#EDA measured at 288 K (dark red squares). B) CO_2 adsorption isotherm of MOF-303#EDA measured at 298 K (red diamonds). C) CO_2 adsorption isotherm of MOF-303#EDA measured at 308 K (orange triangles). D) CO_2 adsorption isotherm of MOF-303#EDA measured at 318 K (yellow circles). Filled and empty symbols represent sorption and desorption branches, respectively.

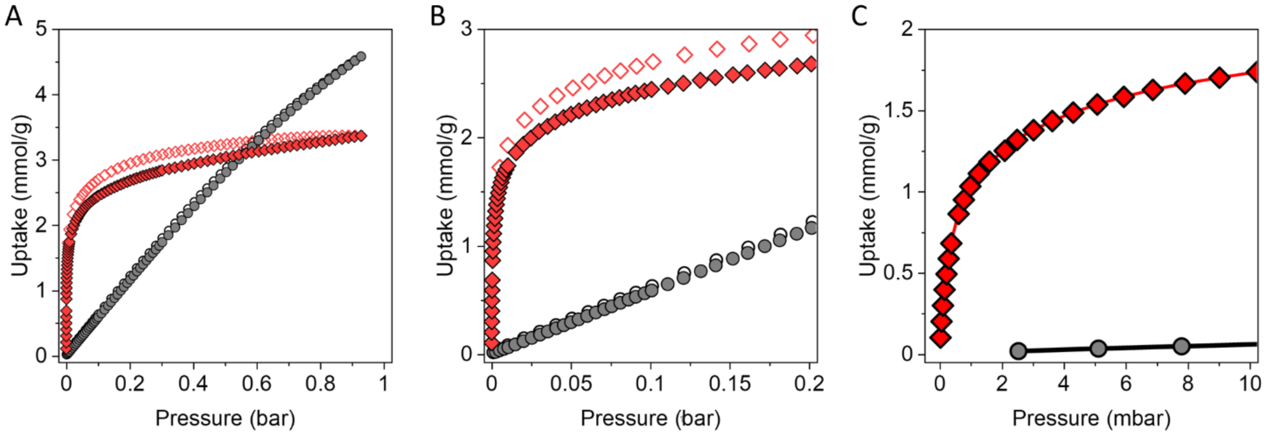

Figure S26. Comparison between the CO_2 adsorption isotherms of MOF-303#EDA (red diamonds) and MOF-303 (grey circles) measured at 298 K and up to (A) 1 bar, (B) 0.2 bar, and (C) 10 mbar (0.01 bar). In panels A and B, the adsorption and desorption branches are represented with full and empty symbols, respectively. In panel C, only the adsorption branches are shown for clarity.

Table S5. Performance metrics of MOF-303#EDA in powder and pellet form.

Sample	CO_2 uptake at 450 ppm[a] (mmol/g)	CO_2 uptake at 1000 ppm[a] (mmol/g)	CO_2 uptake at 50 mbar[a] (mmol/g)	CO_2 uptake at 100 mbar[a] (mmol/g)	CO_2 uptake at 150 mbar[a] (mmol/g)	Qst[b] (kJ/mol)	Cyclability[c]	Sorbent regeneration conditions[d]
MOF-303#EDA	0.71	1.03	2.21	2.44	2.58	55	> 10 cycles	T = 60°C<br>t = 2h<br>He flow

[a]Measured at 298 K from the single-component CO_2 adsorption isotherm. [b]Measured at a 1 mmol/g CO_2 loading. [c]Cyclability was assessed by measuring multiple breakthrough cycles with a 5:95 CO_2/N_2 gas mixture at 298 K and a total flow rate of 5 sccm on MOF-303#EDA in pelletized form. [d]The regeneration process was obtained with a He flow rate of 10 sccm on MOF-303#EDA in pelletized form.

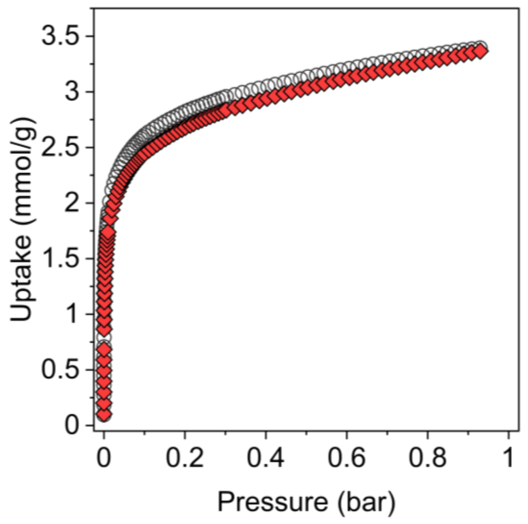

Figure S27. Cyclability of the CO_2 adsorption isotherm of MOF-303#EDA. CO_2 adsorption isotherm of freshly prepared MOF-303#EDA collected at 298 K (black circles) and CO_2 adsorption isotherm of MOF-303#EDA collected at 298 K after degassing the sample on the 3Flex instrument under high vacuum at 60°C for 24 hours.

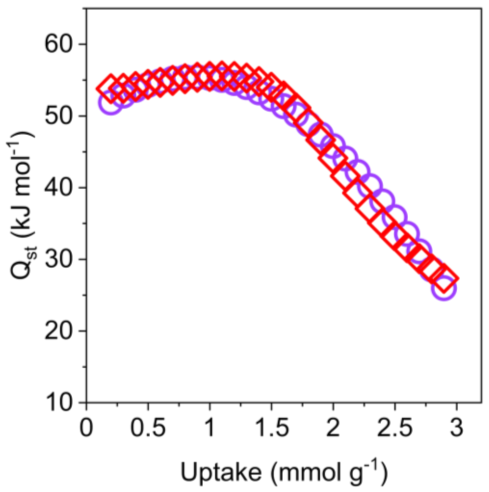

Figure S28 CO_2 isosteric heat of adsorption for MOF-303#EDA calculated from the adsorption isotherms measured at 288 K, 298 K, 308 K, and 318 K according to the Van't Hoff equation, using the dual-site Langmuir-Freundlich isotherm for the fittings of the experimental data (red diamonds) and the virial analysis (violet circles).

Table S6. Performance of representative benchmark materials from literature, used as reference values in this study.

**Material**	**CO_2 uptake (mmol g^{-1})**		**Qst (kJ mol^{-1})**	**Ref.**
	CO_2 (15%)	CO_2 (400 ppm)		
**MOF**				
**MOF-303#EDA (This work)**	**2.5**	**0.71 (450 ppm)**	**55**	**This Work**
en-Mg-MOF-74	-	1.51	-	[23]
TEPA-Mg-MOF-74	6.06	-	-	[24]
mmen-Mg_2(dobpdc)	3.5	2	71	[25]
en-Mg_2(dobpdc)	3.6	2.83 (390 ppm)	50	[23]
men-Mg_2(dobpdc)	3.6	0.41	65-77	[26]
ipen-Mg_2(dobpdc)	3.47	0.06	76-85	[27]
pn-Mg_2(dobpdc)	4.01	1.76	-	[28]
nmen-Mg_2(dobpdc)	2.92	0.76	60-72	[27]
epn-Mg_2(dobpdc)	-	1.76	-	[28]
Nmpn-Mg_2(dobpdc)	-	1.76	-	[28]
MIL-101(Cr)-TEPA	1.25	-	43	[29]
MIL-101(Cr)-TREN	-	0.35	-	[30]
MIL-101(Cr)-SO_3H-TAEA	2.28	1.12	87	[31]
MIL-101(Cr)-PEI 300	4.1	-	-	[32]
MIL-101(Cr)-en	2.1	-	80	[33,34]
MIL-101(Cr)-mmen	1.4	-	80	[28,33]
CuBTTri-pip	0.75	-	96	[35]
CuBTTri-mmen	2.38	-	96	[36]
MOF-808-EDA	0.932	0.27	-	[37]
MOF-808-Lys	1.634	0.61	-	[37]
MOF-808-TAPA	1.055	0.50	-	[37]
TAEA-Ac-$Zn_4O(NH_2\text{-BDC})_{1.2}(\text{BrAcBH-BDC})_{1.8}$	0.16	-	62.5	[38]
CALF-20	2.71	-	39	[39]
COF-999	-	0.96	53	[40]

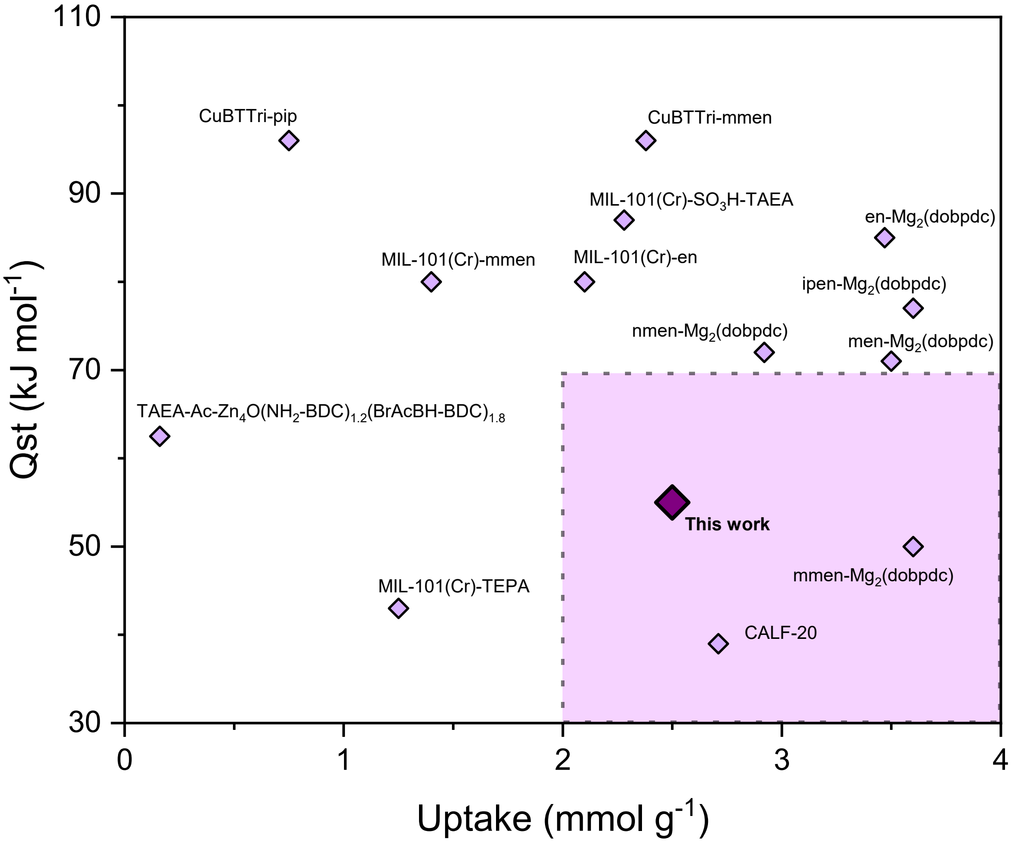

Figure S29 Relationship between Q_{st} and CO_2 uptake at 150 mbar for benchmark materials previously reported in the literature, shown here for performance comparison with the present study.

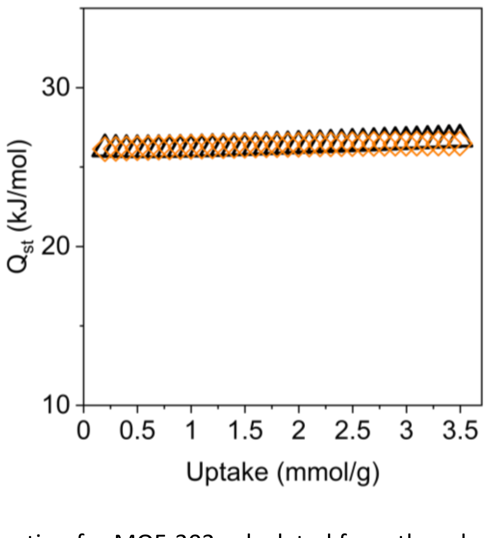

Figure S30 CO_2 isosteric heat of adsorption for MOF-303 calculated from the adsorption isotherms measured at 288 K, 298 K, and 308 K according to the Van't Hoff equation, using the dual-site Langmuir-Freundlich isotherm for the fittings

of the experimental data (black triangles) and the virial analysis (orange diamonds). The isosteric heat of adsorption for MOF-303 is 26 kJ/mol up to a CO_2 loading of 3.5 mmol/g.

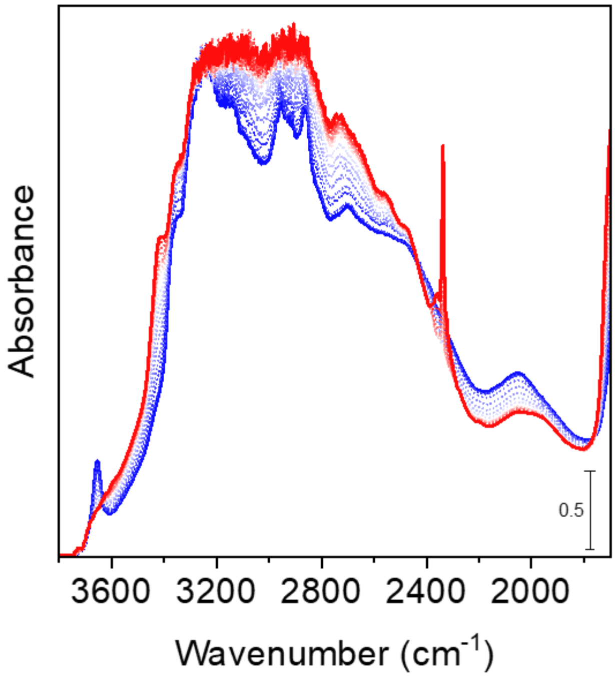

Figure S31 IR spectra in the 3800-1700 cm^{-1} range during CO_2 loading into MOF303#EDA sample.

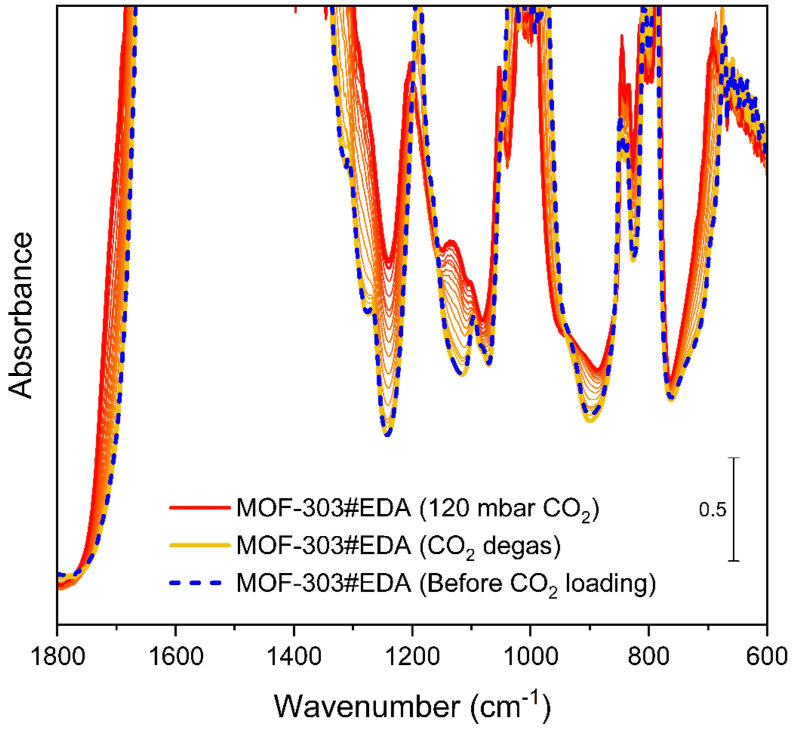

Figure S32 IR spectra in the 1800-600 cm^{-1} range upon CO_2 outgassing from MOF303#EDA sample.

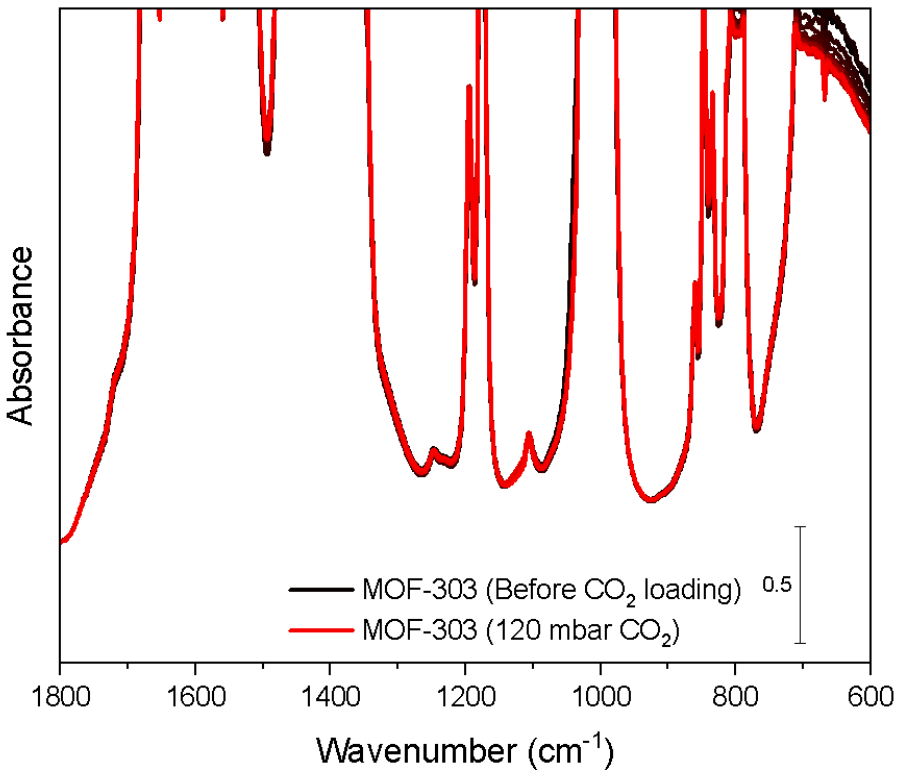

Figure S33 IR spectra of MOF-303 in the 1800-600 cm^{-1} range during CO_2 loading.

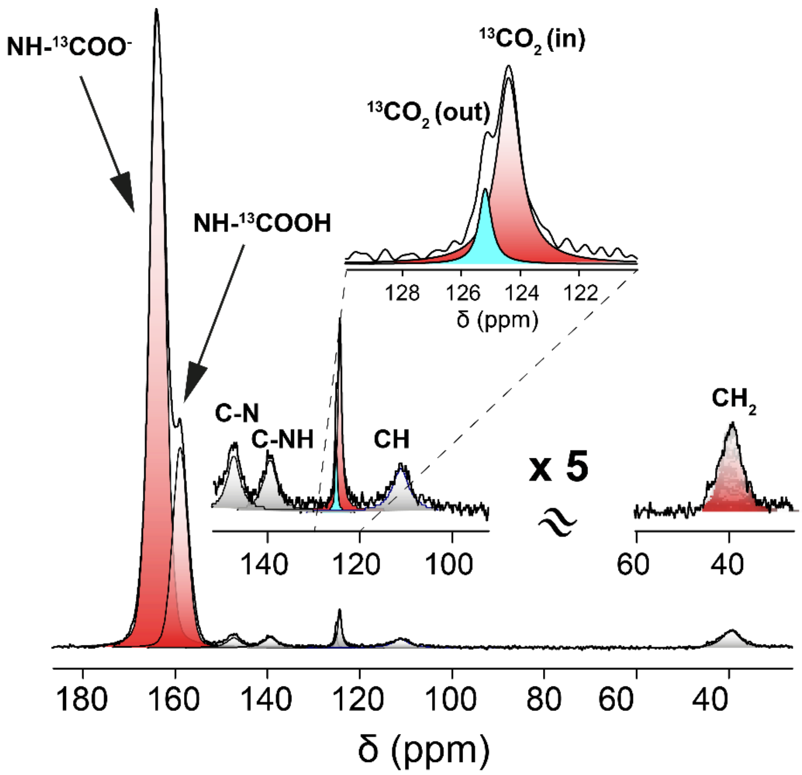

Figure S34 Quantitative ^{13}C SPE MAS NMR spectrum of MOF-303#EDA·CO_2 with enriched-^{13}C CO_2 (99%) collected at 7.04 T and 290 K with a spinning speed of 11.0 kHz and a recycle delay of 60 s. The signals of free $^{13}CO_2$ (125.2 ppm) and physisorbed $^{13}CO_2$ (124.4 ppm) are evident in the enlarged region.

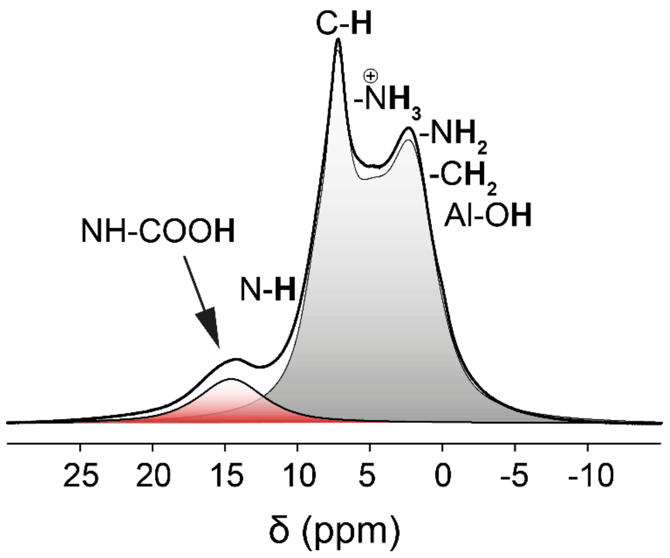

Figure S35 Quantitative ^{1}H SPE MAS NMR spectrum of MOF-303#EDA-CO_2 collected at 14.1 T and 298 K with a spinning speed of 30.0 kHz and a recycle delay of 20 s.

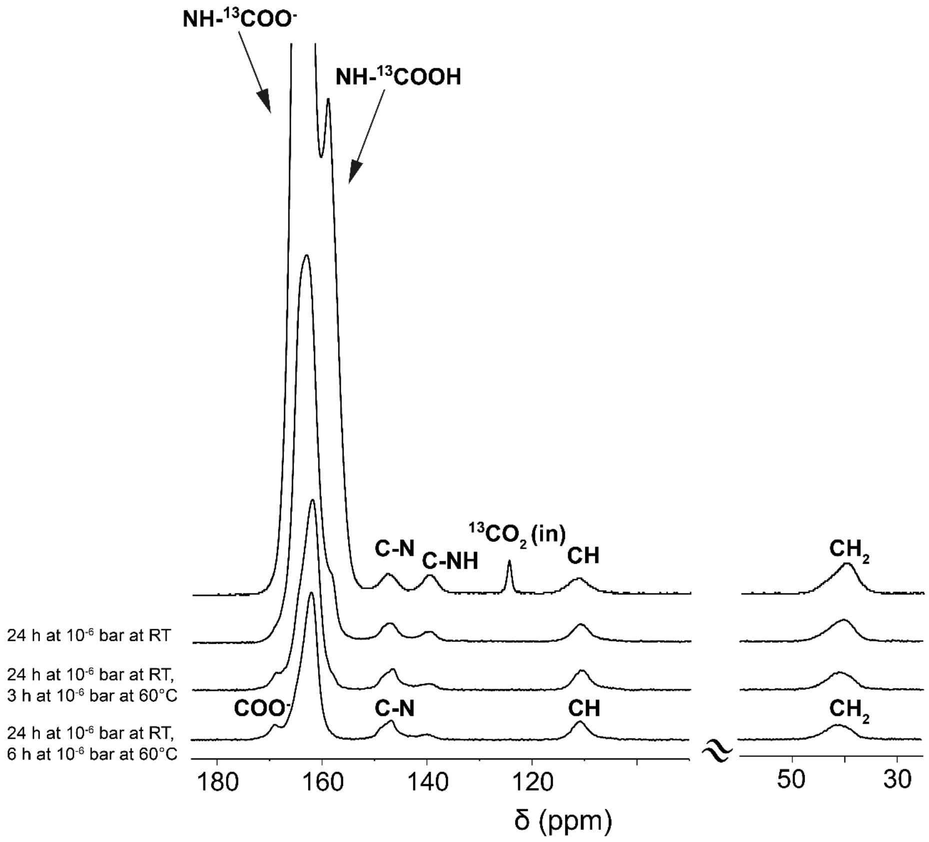

Figure S36 ^{1}H-^{13}C CP MAS NMR spectra of MOF-303#EDA-CO_2 with enriched-^{13}C CO_2 (99%) collected at 7.04 T and 290 K with a spinning speed of 11.0 kHz and a contact time of 2 ms after different (vacuum/ thermal) treatment. The quantification of carbamic acid and carbamate after each vacuum/temperature treatment was performed by following the intensities of their respective resonances referred to the intensity of pyrazole CH signal set equal to 1.

Table S7 ^{13}C chemical shifts of MOF-303#EDA-CO_2 with enriched-^{13}C CO_2 (99%) from the simulation of ^{13}C SPE MAS NMR spectrum collected at 7.04 T and 290 K with a spinning speed of 11 kHz and a recycle delay of 60 s. ^{1}H chemical shifts from the simulation of quantitative ^{1}H SPE MAS NMR spectrum collected at 14.1 T and 298 K with a spinning speed of 30 kHz with a recycle delay of 20 s.

**MOF-303#EDA·CO_2**	**Assignment**	**^{13}C SPE MAS δ (ppm)**
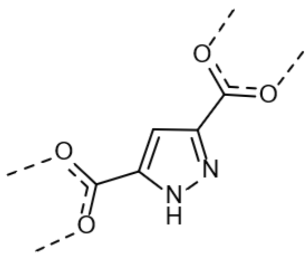	**C**OO$^-$	---
	**C**N	147.3
	**C**NH	139.5
	**C**H	111.2
	NH-^{13}**C**OO$^-$	163.9
	NH-^{13}**C**OOH	159.0
	^{13}**C**O_2out	125.2
	^{13}**C**O_2in	124.4
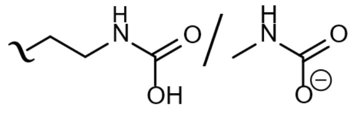	**C**H_2	39.5
	**Assignment**	**^{1}H SPE MAS δ (ppm)**
	NHCOO**H**	14.7

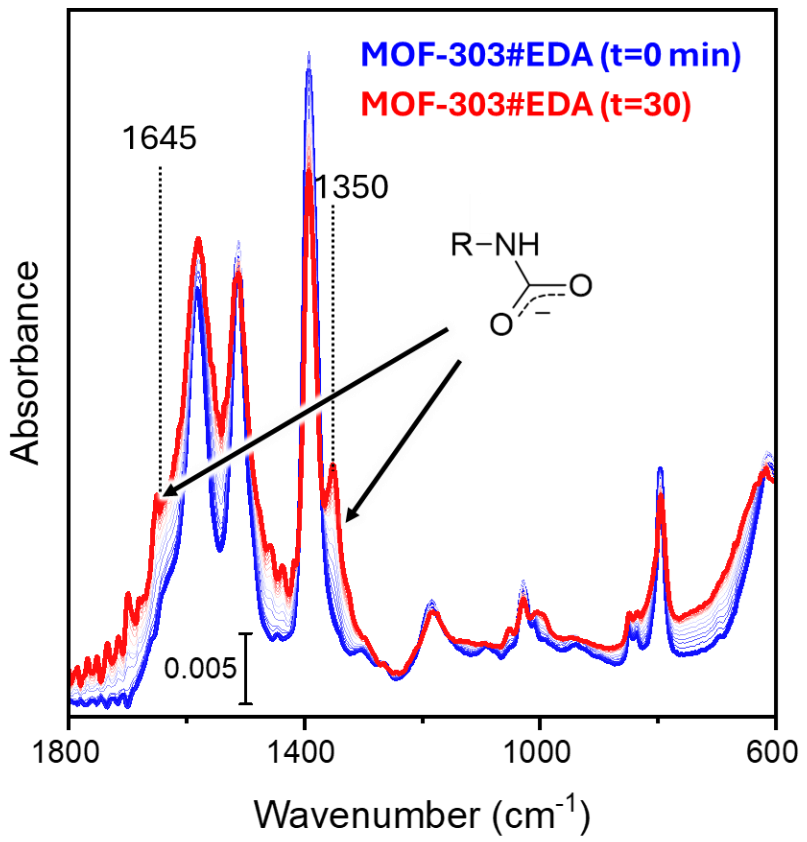

Figure S37 IR spectrum of MOF-303#EDA exposed to ambient conditions. The blue curve represents the spectra right after the exposure whereas the red curve is the spectra after 30 min of exposure.

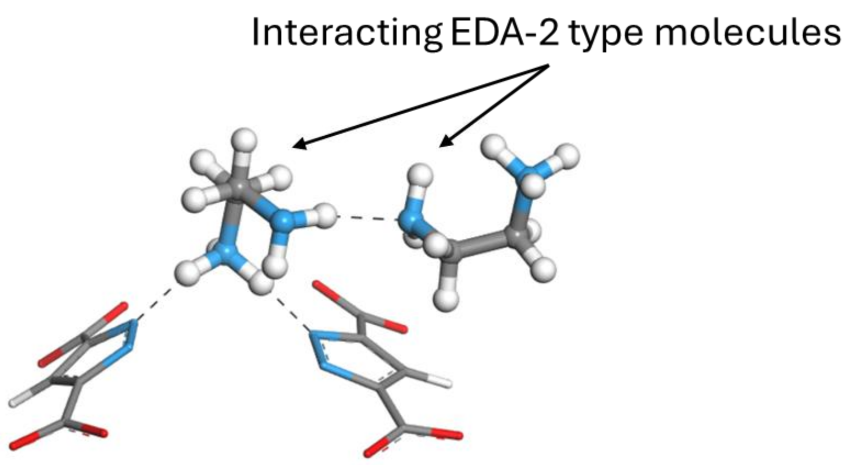

Figure S38 Fragment extracted from optimized structure **S_1** that shows how two EDA-2 type molecules can interact by H-bonding inside MOF-303 channel. For the sake of clarity, EDA-1 molecules were omitted.

Table S8 Energies and geometric parameters for the CO_2 adsorption into MOF-303#EDA and subsequent carbamate formation.*

Structure	Energy Ha	ΔE^a kJ mol^{-1}	$\Delta E(CPC)^b$ kJ mol^{-1}	C–N Distance^c Å	O–C–O Angle^d Degree
CO_2	-188.451571	–	–	–	180
S_1	-8883.483659	–	–	–	–
S_1 + CO_2	-9260.386802	0	0	–	–
S_2	-9260.425796	-51.19	-36.93	$> 3^e$	178.16
S_3	-9260.425002	-50.15	-29.30	1.66	138.85
S_4	-9260.460694	-97.00	–	1.37	125.84

[a]The relative energy is given per single CO_2 molecule inserted in the unit cell.
[b]Energies corrected for the basis set superposition error (BSSE) by applying the Boys–Bernardi counterpoise correction (CPC).
[c]C-N distance between Carbon of CO_2 and free Nitrogen of EDA molecule.
[d]O-C-O angle of CO_2 in the gas phase and of carboxylate moieties of carbamate.
[e]Structure S_2 as described in the text contain not interacting CO_2. The precise distance is not specified.

* In the present calculations, the pyrazole unit in the **S_4** structure appears deprotonated in contrast with ^{13}C MAS NMR spectra. Currently, in the modelling, no subsequent structural rearrangement yielding a protonated pyrazole upon CO_2 uptake was considered.

S4. Pellet preparation and breakthrough measurements

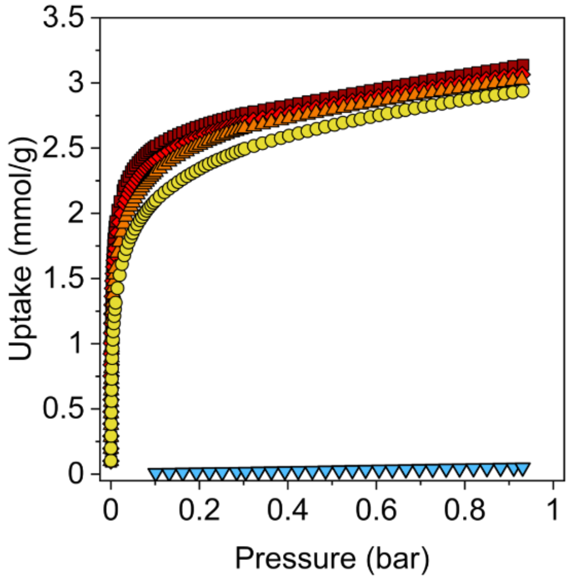

Figure S39 CO_2 adsorption isotherms of MOF-303#EDA-pellet at 288 K (dark red squares), 298 K (red diamonds), 308 K (orange triangles), and 318 K (yellow circles). N_2 adsorption isotherm collected at 298 K (down-pointing light blue triangles). Only the adsorption branches are shown for clarity.

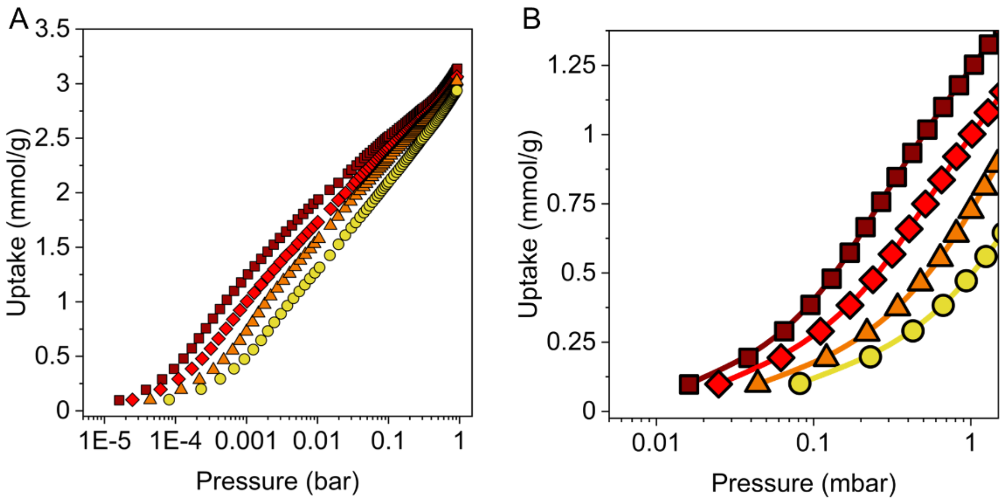

Figure S40 A) CO_2 adsorption isotherms of MOF-303#EDA-pellet measured at 288 K (dark red squares), 298 K (red diamonds), 308 K (orange triangles), and 318 K (yellow circles) in semilogarithmic scale. B) Magnification of the 0.005 - 1.5 mbar region. Only the adsorption branches are shown for clarity.

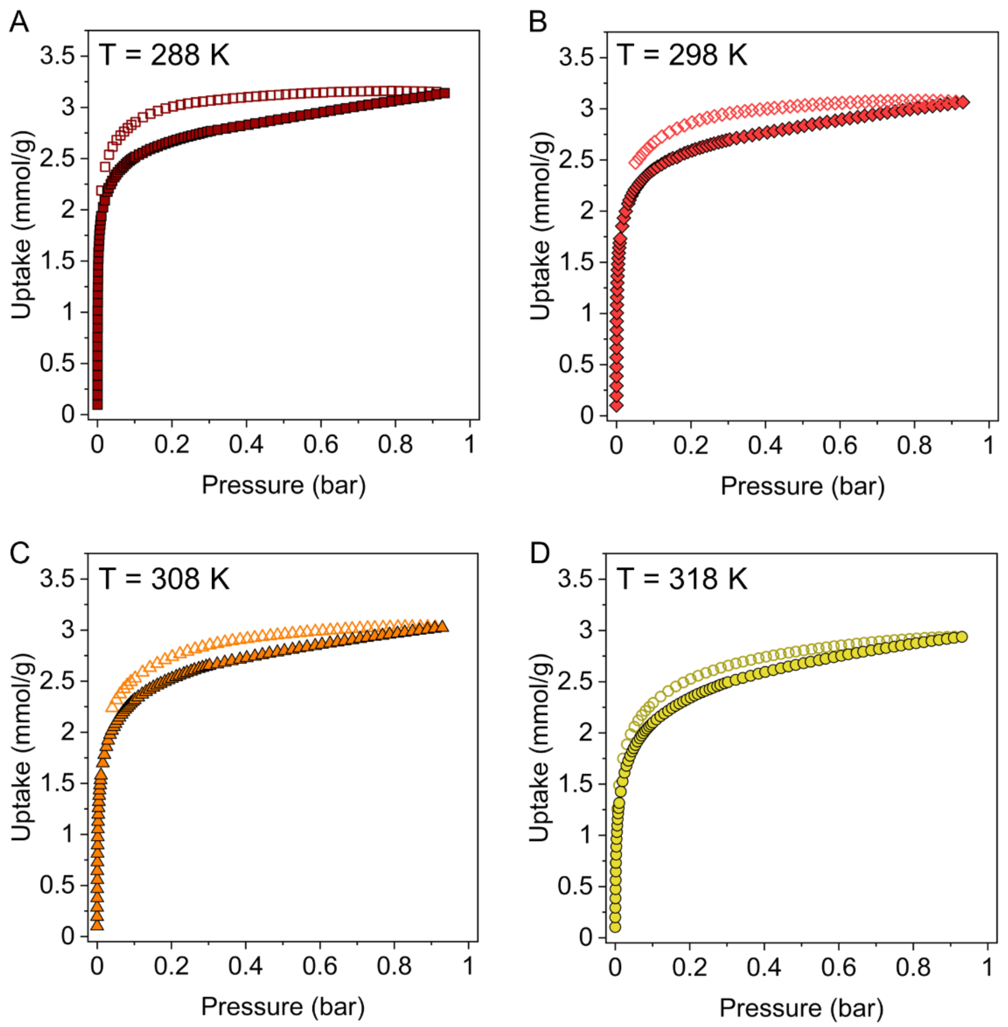

Figure S41 A) CO_2 adsorption isotherm of MOF-303#EDA-pellet measured at 288 K (dark red squares). B) CO_2 adsorption isotherm of MOF-303#EDA-pellet measured at 298 K (red diamonds). C) CO_2 adsorption isotherm of MOF-303#EDA-pellet measured at 308 K (orange triangles). D) CO_2 adsorption isotherm of MOF-303#EDA-pellet measured at 318 K (yellow circles). Filled and empty symbols represent sorption and desorption branches, respectively.

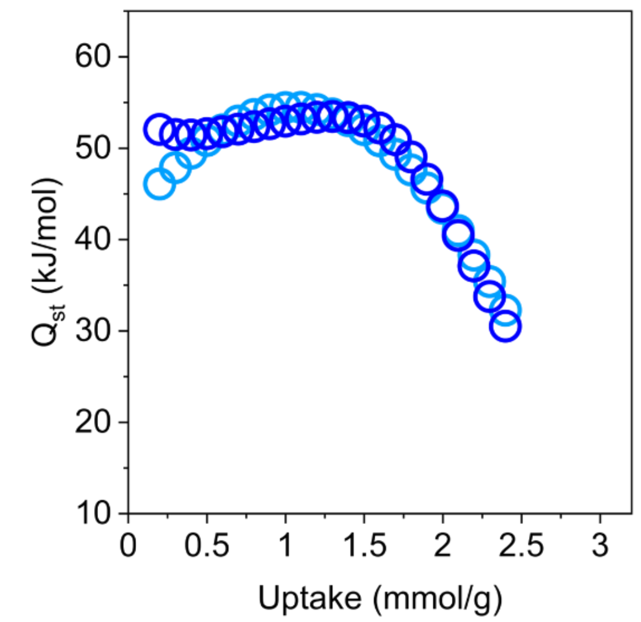

Figure S42 CO_2 isosteric heat of adsorption for MOF-303#EDA-pellet calculated from the adsorption isotherms measured at 288 K, 298 K, 308 K, and 318 K according to the Van't Hoff equation, using the dual-site Langmuir Freundlich isotherms for the fittings of the experimental data (blue circles) and the virial analysis (light blue circles). The isosteric heat of adsorption at low coverage (CO_2 uptake = 0.2 mmol/g) is 52 kJ/mol (Van't Hoff equation).

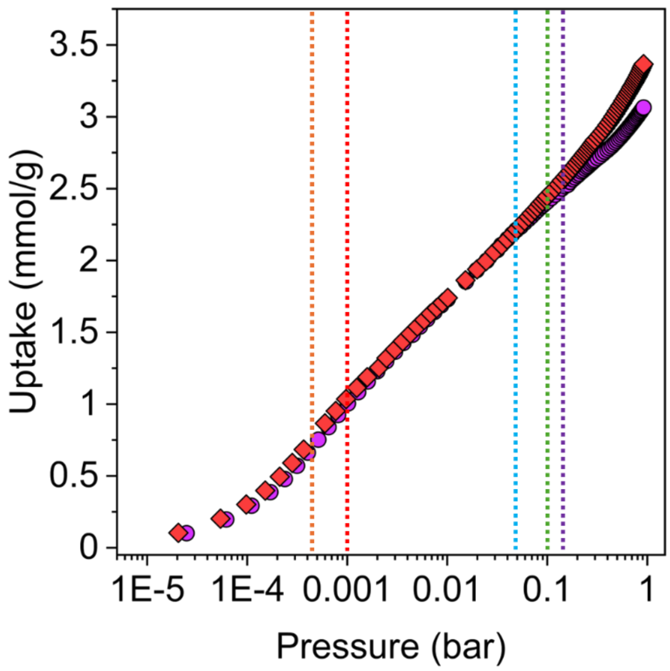

Figure S43 CO_2 adsorption isotherm measured at 298 K for MOF-303#EDA powder (red diamonds) and pellets (purple circles) in semilogarithmic scale. The vertical dotted lines highlight pressure values relevant for CO_2 capture from air (450 ppm, orange, and 1000 ppm, red) and flue gases (50 mbar, light blue; 100 mbar, green, and 150 mbar, violet). The CO_2 uptakes at these relevant pressures for MOF-303#EDA in powder and pellet form are summarized in Table S8.

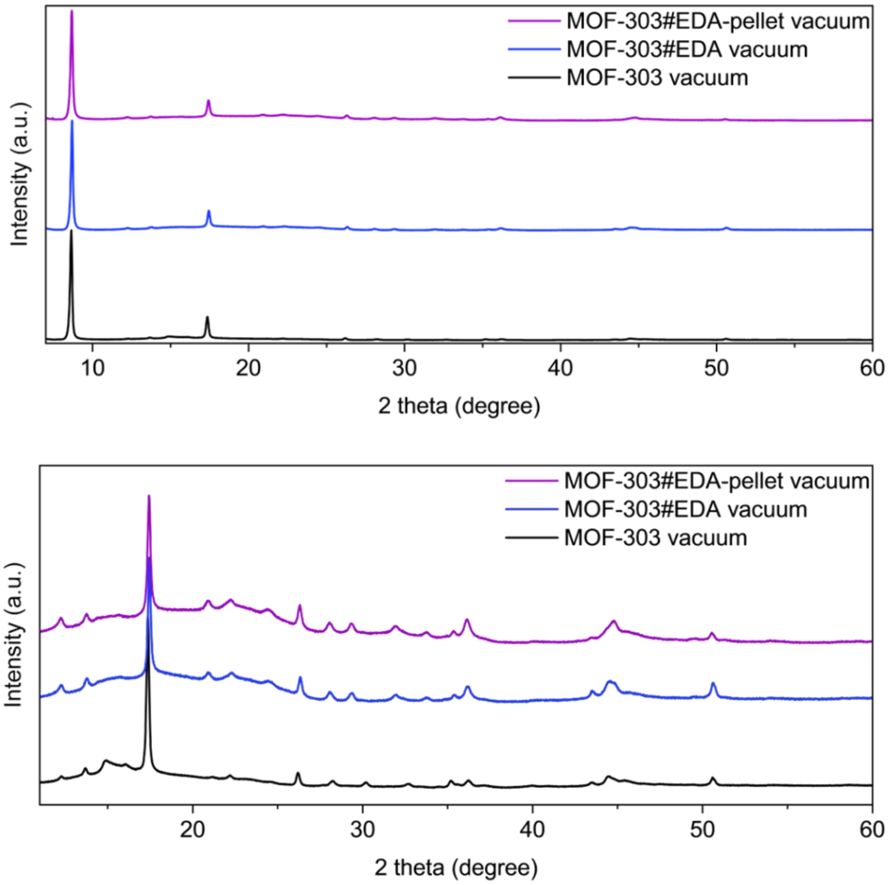

Figure S44 Top: powder X-ray diffraction patterns of MOF-303 (black), MOF-303#EDA (blue) and MOF-303#EDA-pellet (purple) measured using a Rigaku Smartlab diffractometer equipped with an Anton Paar TTK 600 environmental chamber under dynamic vacuum conditions. Bottom: enlargement between 11° and 60° 2 theta.

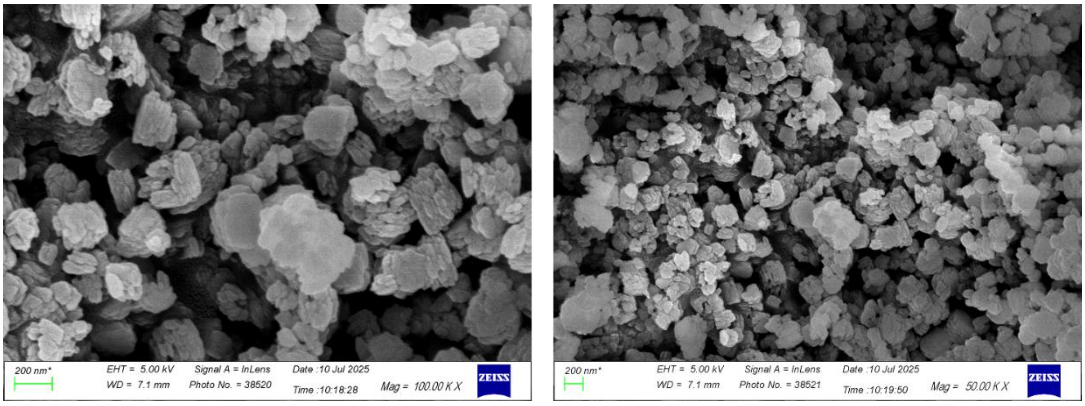

Figure S45 SEM images of sample MOF-303#EDA shaped into self-supporting pellets.

Table S9 CO_2 uptake of sample MOF-303#EDA in powder and pellet forms at 298 K.

Sample	CO_2 uptake at 450 ppm (mmol/g)	CO_2 uptake at 1000 ppm (mmol/g)	CO_2 uptake at 50 mbar (mmol/g)	CO_2 uptake at 100 mbar (mmol/g)	CO_2 uptake at 150 mbar (mmol/g)
MOF-303#EDA - powder	0.71	1.03	2.21	2.44	2.58
MOF-303#EDA - pellet	0.65	0.99	2.21	2.41	2.51

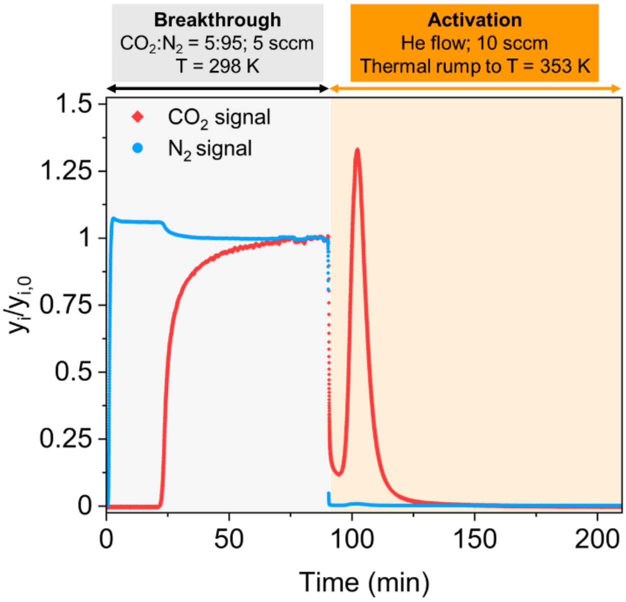

Figure S46 Mass spectrometer signals for CO_2 (red) and N_2 (light blue) during a breakthrough experiment, showing the composition of the outlet gas mixture as a function of time (CO_2:N_2 = 0.05:0.95, T =298 K). The initial time corresponds to the feeding of the CO_2:N_2 mixture (5 sccm in total) in the column inlet. After 90 minutes, the inlet was switched to a He flow (10 sccm) and the sample was heated to 70°C with a thermal ramp of ~ 5°C/min and kept at 70°C for 2 hours to release the adsorbed CO_2.

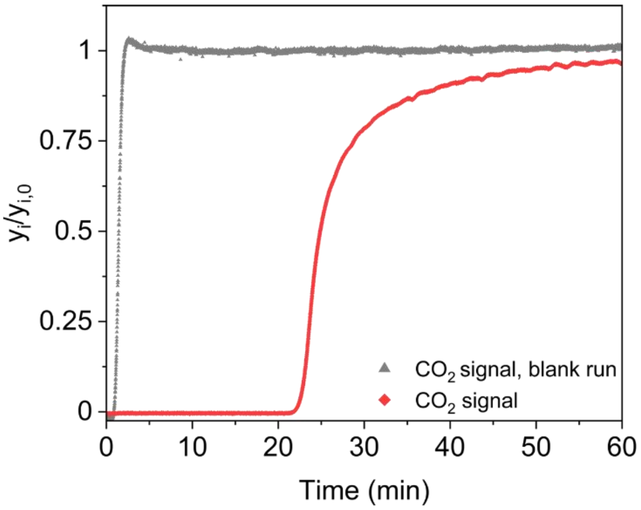

Figure S47 Mass spectrometer signals for CO_2 during a breakthrough experiment (CO_2:N_2 = 0.05:0.95, T =298 K, total flow = 5 sccm) with a non-adsorptive material (blank measurement, grey line), and with MOF-303#EDA-pellet (red line).

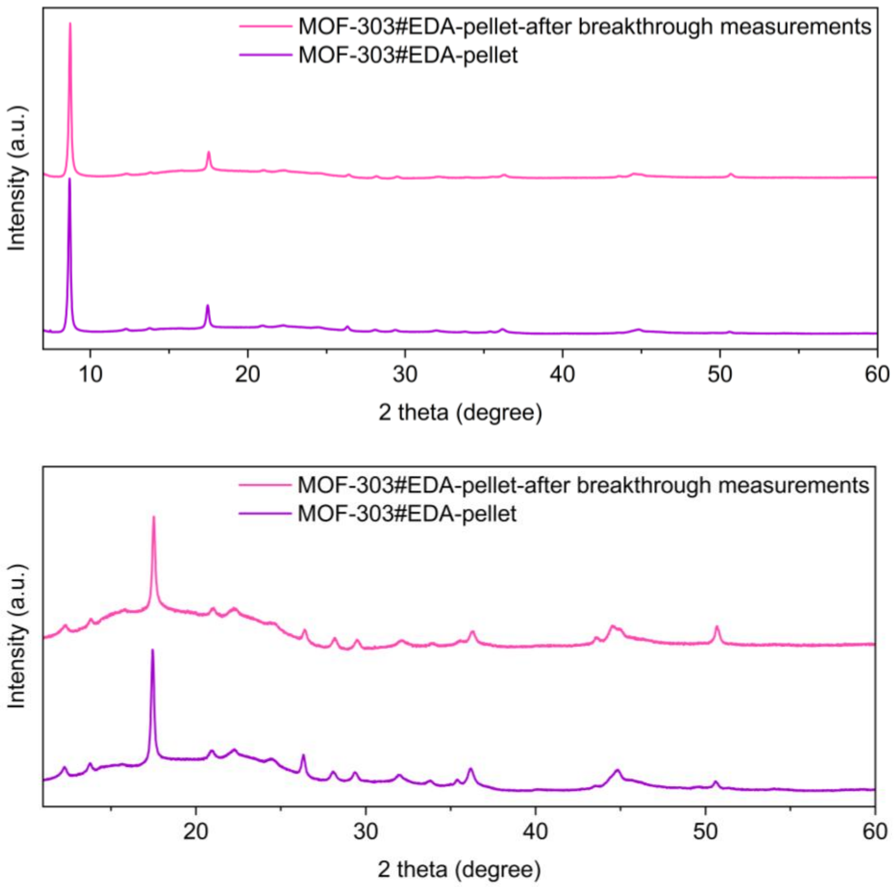

Figure S48. Top: powder X-ray diffraction patterns of MOF-303#EDA-pellet before (purple) and after (pink) breakthrough measurements collected using a Rigaku Smartlab diffractometer equipped with an Anton Paar TTK 600 environmental chamber under dynamic vacuum conditions. Bottom: enlargement of the PXRD patterns between 11° and 60° 2 theta. The intensity was scaled up five times.

Appendix

Evaluation of specific surface area, pore size distribution, and pore volume

MOF-303 N_2 77 K

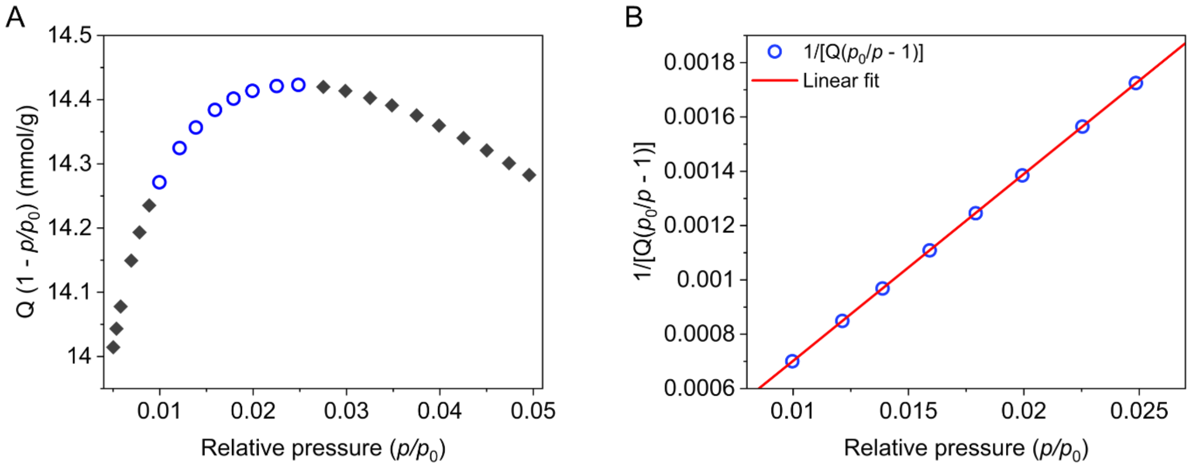

Figure A1 A) Rouquerol plot in the relative pressure range between 0.005 and 0.05 p/p_0. The blue circles indicate the experimental data used to evaluate the BET surface area. B) Fitting of the BET equation in the selected pressure range for N_2 on MOF-303 at 77 K (from 0.01 to 0.025 p/p_0).

Table A1 Fitting parameters of the BET equation.

Equation	y = a + b*x
Plot	1/[Q(p_0/p - 1)]
Weight	No Weighting
BET surface area	1417.2979 ± 1.0400 m^2/g
Intercept	1.17127E-5 ± 9.00129E-7 g/mmol
Slope	0.06882 ± 5.05134E-5 g/mmol
C	5875.492022
Q_m	14.52758 mmol/g
Molecular cross-section	0.1620 nm^2
Residual Sum of Squares	2.84743E-12
Pearson's r	0.9999984
R-Square (COD)	0.9999968
Adj. R-Square	0.9999962

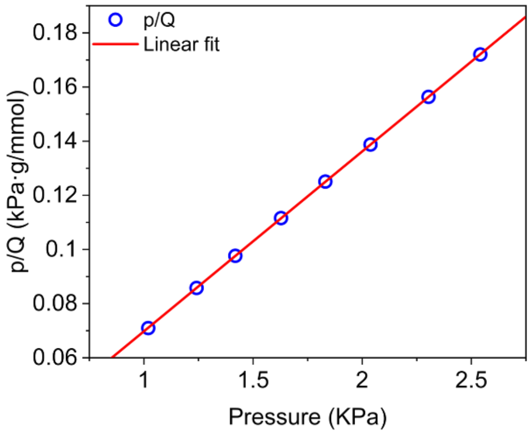

Figure A2 Fitting of the Langmuir equation in the selected pressure range for N_2 on MOF-303 at 77 K (from 1 to 2.5 KPa).

Table A2 Fitting parameters of the Langmuir equation.

Equation	y = a + b*x
Plot	p/Q
Weight	No Weighting
Langmuir surface area	1469.0846 ± 1.6182 m^2/g
Intercept	1.17127E-5 ± 9.00129E-7 kPa·g/mmol
Slope	0.06882 ± 5.05134E-5 g/mmol
b	20.88953 1/kPa
Q_m	15.05840 mmol/g
Molecular cross-section	0.1620 nm^2
Residual Sum of Squares	6.22857E-8
Pearson's r	0.9999964
R-Square (COD)	0.9999927
Adj. R-Square	0.9999915

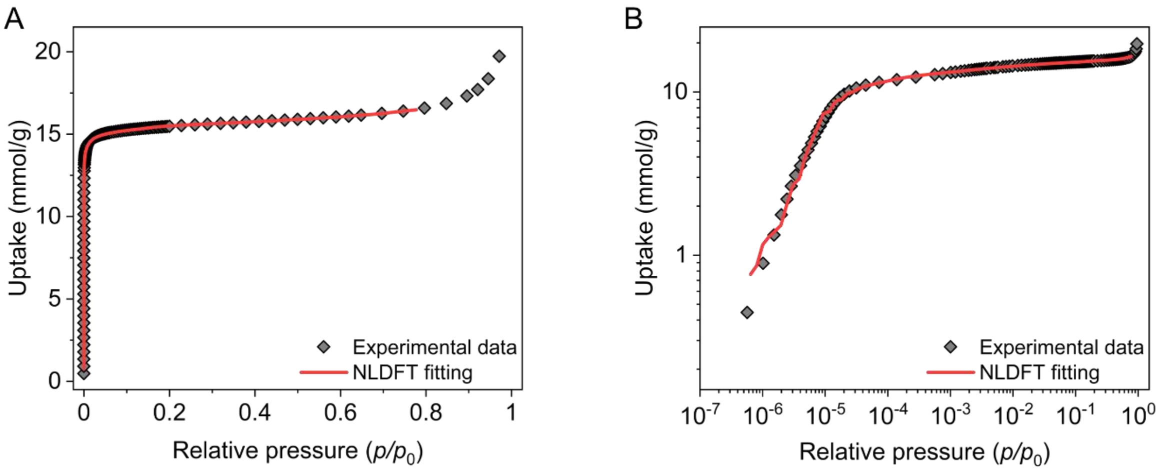

Figure A3 A) Experimental data for the N_2 adsorption at 77 K of MOF-303 (grey diamonds) and NLDFT fitting (red line) of the adsorption isotherm in natural scale. B) Experimental data (grey diamonds) and NLDFT fitting (red line) of the adsorption isotherm in double logarithmic scale (Standard Deviation of Fit: 0.139896 mmol/g).

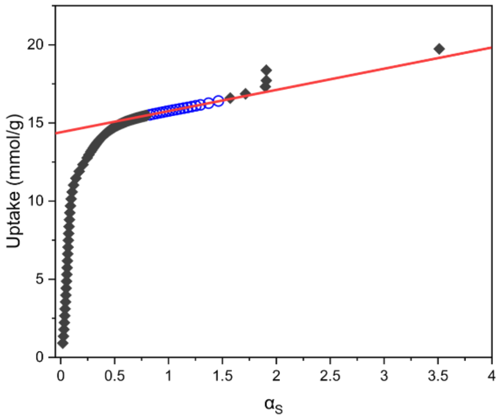

Figure A4 α_S plot of the N_2 adsorption isotherm for MOF-303 measured at 77 K. The blue circles indicate the experimental data used to evaluate the micropore volume according to the α_S method. The linear fit is reported (red line).

Table A3 Fitting parameters of the α_S plot for MOF-303.

Equation	y = a + b*x
Pore volume	0.500280 cm^3/g
Intercept	14.38996 ± 0.00873 g/mmol
Slope	1.35552 ± 0.00788 g/mmol
Correlation coefficient	0.999747

MOF-303#EDA N_2 77 K

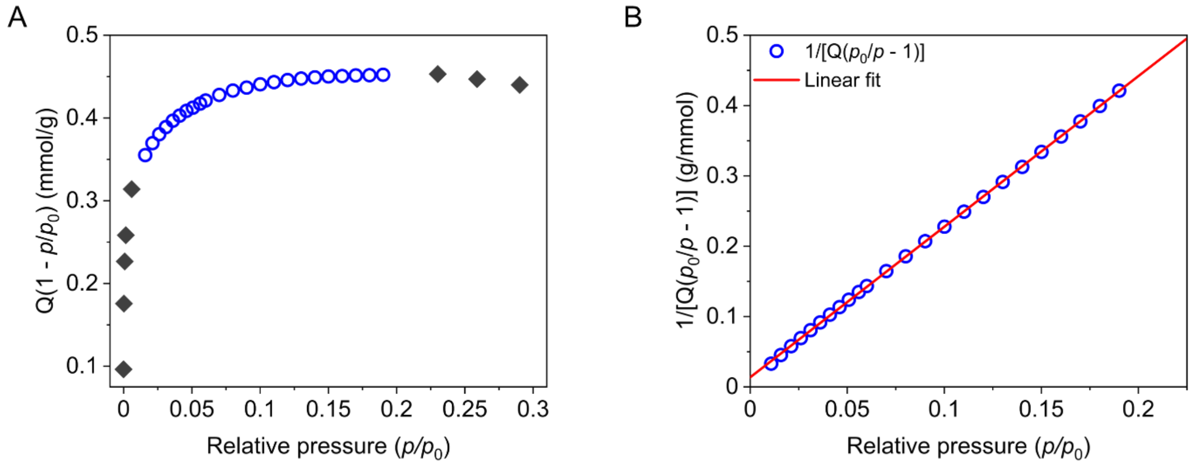

Figure A5 A) Rouquerol plot in the relative pressure range between 0 and 0.3 p/p_0. The blue circles indicate the experimental data used to evaluate the BET surface area. B) Fitting of the BET equation in the selected pressure range for N_2 on MOF-303#EDA at 77 K (from 0.01 to 0.2 p/p_0).

Table A4 Fitting parameters of the BET equation.

Equation	y = a + b*x
Plot	1/[Q(p_0/p - 1)]
Weight	No Weighting
BET surface area	45.2755 ± 0.0967 m^2/g
Intercept	0.01284 ± 4.72475E-4 g/mmol
Slope	2.14195 ± 0.00458 g/mmol
C	167.858731
Q_m	0.46408 mmol/g
Molecular cross-section	0.1620 nm^2
Residual Sum of Squares	3.40641E-5
Pearson's r	0.9999498
R-Square (COD)	0.9998996
Adj. R-Square	0.9998950

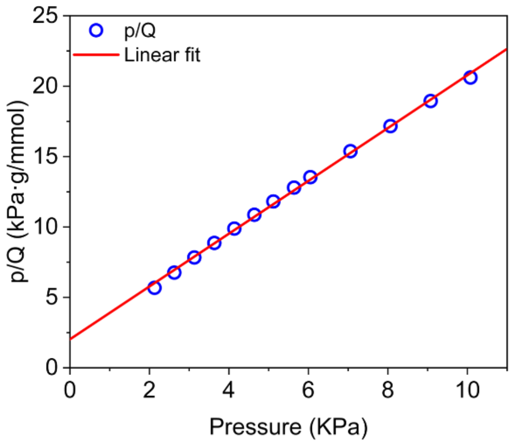

Figure A6 Fitting of the Langmuir equation in the selected pressure range for N_2 on MOF-303#EDA at 77 K (from 2 to 10 KPa).

Table A5 Fitting parameters of the Langmuir equation.

Equation	y = a + b*x
Plot	p/Q
Weight	No Weighting
Langmuir surface area	52.0093 ± 0.6187 m^2/g
Intercept	1.98808 ± 0.13386 kPa·g/mmol
Slope	1.87579 ± 0.02232 g/mmol
b	0.94354 1/kPa
Q_m	0.53311 mmol/g
Molecular cross-section	0.1620 nm^2
Residual Sum of Squares	0.40923
Pearson's r	0.99922
R-Square (COD)	0.99845
Adj. R-Square	0.9983

Evaluation of the isosteric heat of adsorption according to the Van't Hoff equation and the virial method

MOF-303

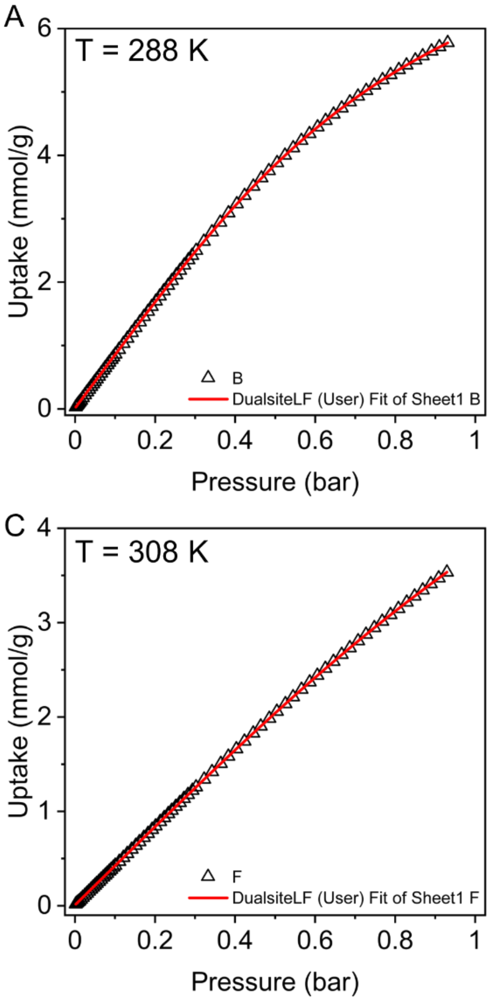

Figure A7 CO_2 adsorption isotherms of MOF-303 (black triangles) and fitting curves according to the dual-site Langmuir-Freundlich model (red lines) measured at (A) 288 K, (B) 298 K, and (C) 308 K.

Table A6 Fitting parameters of the dual-site Langmuir-Freundlich equations for MOF-303.

Model	DualsiteLF (User)		
Equation	((q1*k1*x^n1)/(1+k1*x^n1))+((q2*k2*x^n2)/(1+k2*x^n2))		
Plot	288 K	298 K	308 K
q1	4.87361 ± 0.06504	4.87361 ± 0.06504	4.87361 ± 0.06504
q2	4.1893 ± 0.07747	4.1893 ± 0.07747	4.1893 ± 0.07747
k1	1.9973 ± 0.01148	1.16479 ± 0.00768	0.55629 ± 0.00302
k2	1.86628 ± 0.03014	1.0907 ± 0.01076	0.91372 ± 0.01531
n1	1.75628 ± 0.00657	0.99887 ± 0.00267	1.70377 ± 0.00559
n2	0.98563 ± 0.00209	1.823 ± 0.01279	0.98362 ± 0.00177
Reduced Chi-Sqr	5.54386E-7		
R-Square (COD)	0.9999998	0.9999999	0.9999997
R-Square (COD)	0.9999998		
Adj. R-Square	0.9999998		

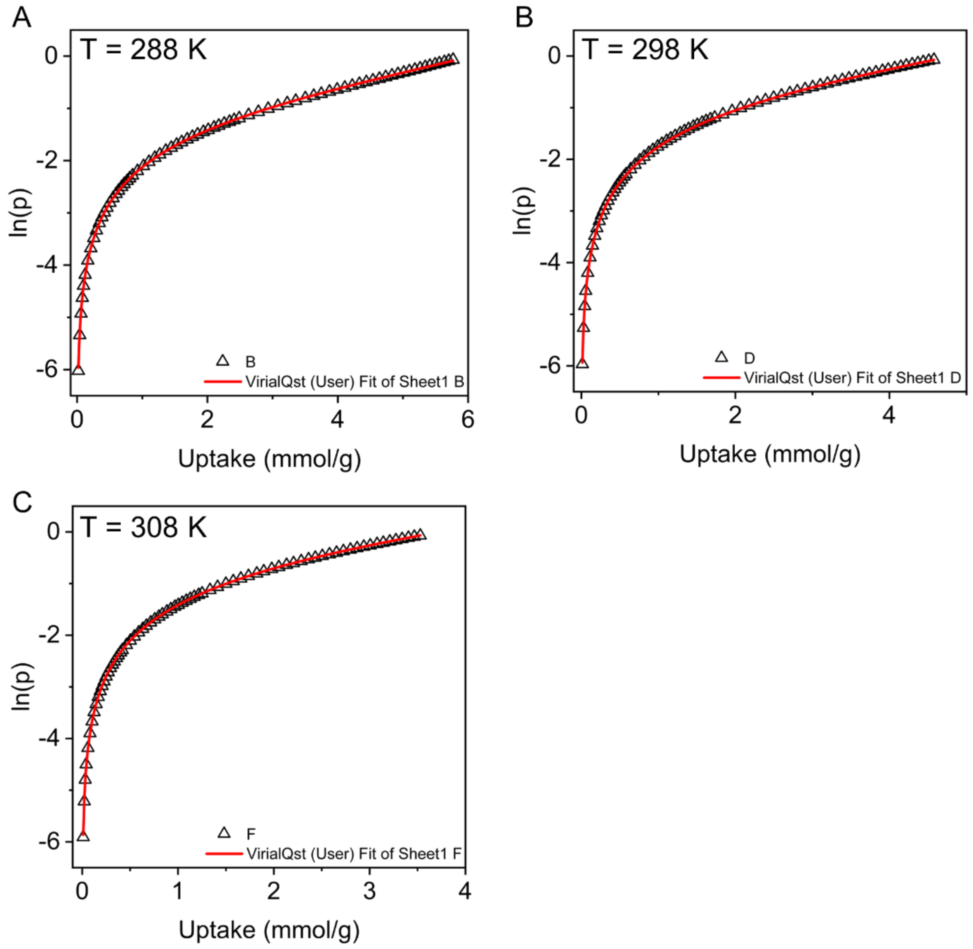

Figure A8 Virial fitting (red line) of the CO_2 adsorption isotherms of MOF-303 measured at (A) 288 K, (B) 298 K, and (C) 308 K.

Table A7 Fitting parameters of the virial equation for MOF-303.

Model	VirialQst (User)		
Equation	Qst=-R*(a0+a1*N+a2*N^2+a3*N^3+a4*N^4+a5*N^5+a6*N^6+a7*N^7+a8*N^8); lnp = ln(N) + (1/T)*(-Qst/R) + (b0+b1*N+b2*N^2+b3*N^3+b4*N^4+b5*N^5);		
Plot	288 K	298 K	308 K
T	288 ± 0	298 ± 0	308 ± 0
a0	-3135.99977 ± 14.13012	-3135.99977 ± 14.13012	-3135.99977 ± 14.13012
a1	-26.27809 ± 7.34596	-26.27809 ± 7.34596	-26.27809 ± 7.34596
a2	3.55245 ± 0.11851	3.55245 ± 0.11851	3.55245 ± 0.11851
a3	0 ± 0	0 ± 0	0 ± 0
a4	0 ± 0	0 ± 0	0 ± 0
a5	0 ± 0	0 ± 0	0 ± 0
a6	0 ± 0	0 ± 0	0 ± 0
a7	0 ± 0	0 ± 0	0 ± 0
a8	0 ± 0	0 ± 0	0 ± 0
b0	8.76703 ± 0.04708	8.76703 ± 0.04708	8.76703 ± 0.04708
b1	0.0682 ± 0.02382	0.0682 ± 0.02382	0.0682 ± 0.02382
b2	0 ± 0	0 ± 0	0 ± 0
b3	0 ± 0	0 ± 0	0 ± 0
b4	0 ± 0	0 ± 0	0 ± 0
b5	0 ± 0	0 ± 0	0 ± 0
Reduced Chi-Sqr	1.3067E-4		
R-Square (COD)	0.99993	0.99993	0.99994
R-Square (COD)	0.99993		
Adj. R-Square	0.99993		

MOF-303#EDA

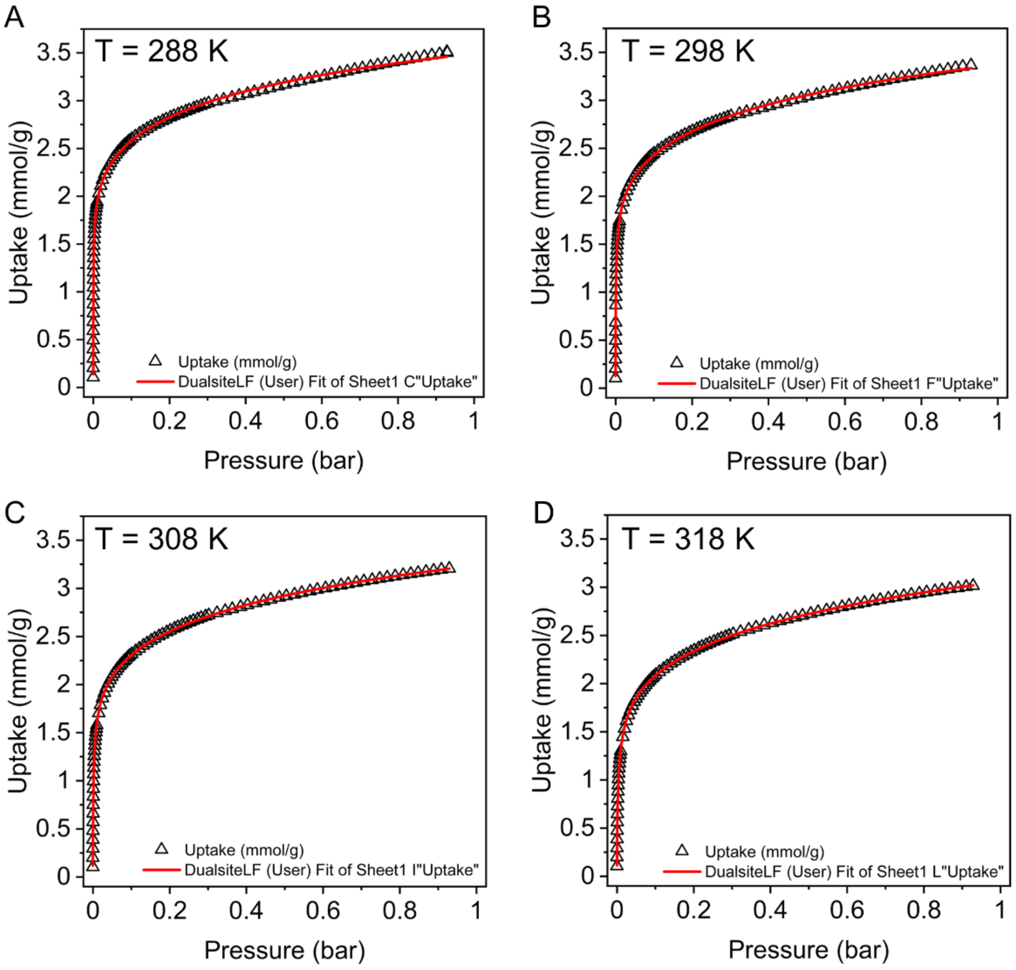

Figure A9 CO_2 adsorption isotherms of MOF-303#EDA (black triangles) and fitting curves according to the dual-site Langmuir-Freundlich model (red lines) measured at (A) 288 K, (B) 298 K, (C) 308 K, and (D) 318 K.

Table A8 Fitting parameters of the dual-site Langmuir-Freundlich equations for MOF-303#EDA.

Model	DualsiteLF (User)			
Equation	((q1*k1*x^n1)/(1+k1*x^n1))+((q2*k2*x^n2)/(1+k2*x^n2))			
Plot	288 K	298 K	308 K	318 K
q1	1.44104 ± 0.10899	1.44104 ± 0.10899	1.44104 ± 0.10899	1.44104 ± 0.10899
q2	5.08878 ± 0.7002	5.08878 ± 0.7002	5.08878 ± 0.7002	5.08878 ± 0.7002
k1	856.36145 ± 336.08429	318.69962 ± 95.79828	204.93316 ± 54.03373	88.04071 ± 18.79299
k2	0.67579 ± 0.10039	0.61163 ± 0.08504	0.54892 ± 0.07082	0.47186 ± 0.05513
n1	0.85054 ± 0.04723	0.79917 ± 0.03837	0.82371 ± 0.03821	0.7758 ± 0.03138
n2	0.36508 ± 0.03537	0.39214 ± 0.03679	0.40356 ± 0.03691	0.44957 ± 0.04025
Reduced Chi-Sqr	2.11409E-4			
R-Square (COD)	0.99953	0.99972	0.99981	0.99988
R-Square (COD)	0.99972			
Adj. R-Square	0.99971			

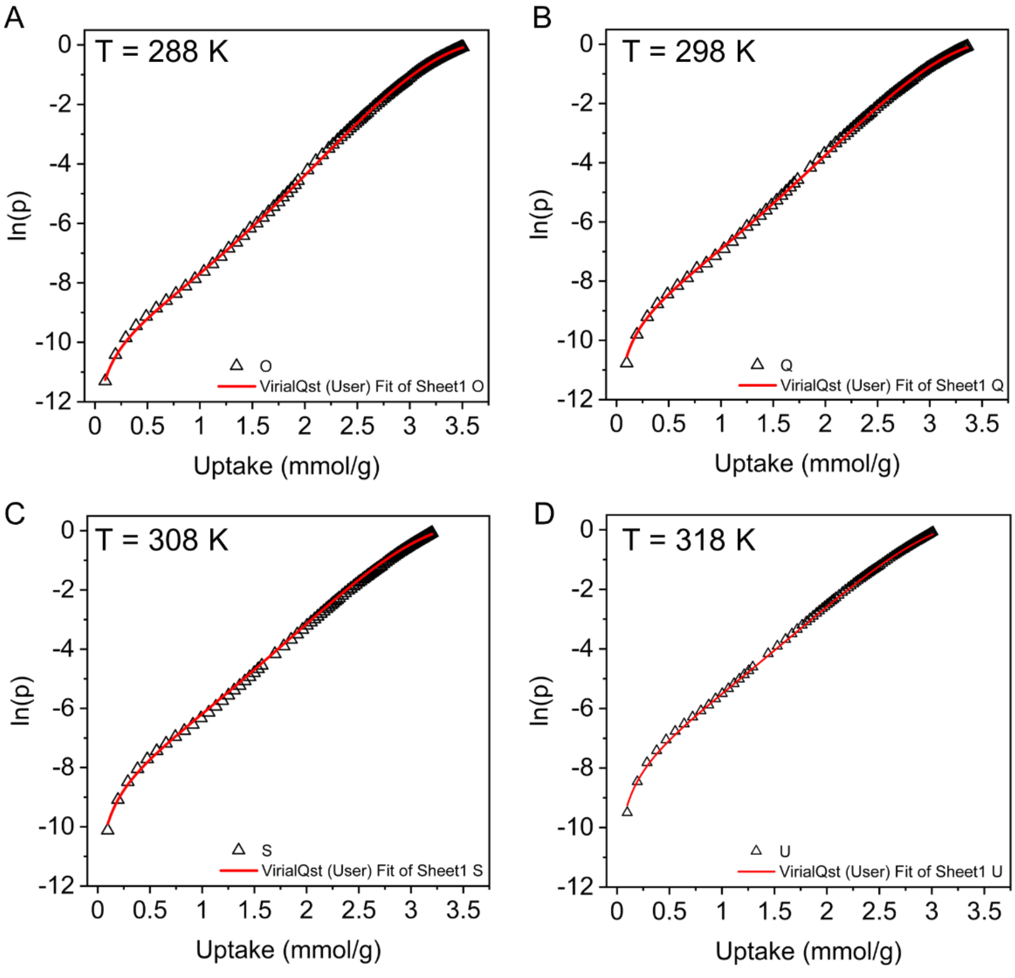

Figure A10 Virial fitting (red line) of the CO_2 adsorption isotherms of MOF-303#EDA measured at (A) 288 K, (B) 298 K, (C) 308 K, and (D) 318 K.

Table A9 Fitting parameters of the virial equation for MOF-303#EDA.

Model	VirialQst (User)			
Equation	Qst=-R*(a0+a1*N+a2*N^2+a3*N^3+a4*N^4+a5*N^5+a6*N^6+a7*N^7+a8*N^8); lnp = ln(N) + (1/T)*(-Qst/R) + (b0+b1*N+b2*N^2+b3*N^3+b4*N^4+b5*N^5);			
Plot	288 K	298 K	308 K	318 K
T	288 ± 0	298 ± 0	308 ± 0	318 ± 0
a0	-5952.82068 ± 120.24652	-5952.82068 ± 120.24652	-5952.82068 ± 120.24652	-5952.82068 ± 120.24652
a1	-1596.28601 ± 140.13622	-1596.28601 ± 140.13622	-1596.28601 ± 140.13622	-1596.28601 ± 140.13622
a2	850.25782 ± 42.70527	850.25782 ± 42.70527	850.25782 ± 42.70527	850.25782 ± 42.70527
a3	67.9833 ± 12.35872	67.9833 ± 12.35872	67.9833 ± 12.35872	67.9833 ± 12.35872
a4	-19.02086 ± 1.68445	-19.02086 ± 1.68445	-19.02086 ± 1.68445	-19.02086 ± 1.68445
a5	0 ± 0	0 ± 0	0 ± 0	0 ± 0
a6	0 ± 0	0 ± 0	0 ± 0	0 ± 0
a7	0 ± 0	0 ± 0	0 ± 0	0 ± 0
a8	0 ± 0	0 ± 0	0 ± 0	0 ± 0
b0	11.62545 ± 0.39886	11.62545 ± 0.39886	11.62545 ± 0.39886	11.62545 ± 0.39886
b1	6.32807 ± 0.47034	6.32807 ± 0.47034	6.32807 ± 0.47034	6.32807 ± 0.47034
b2	-2.56611 ± 0.12614	-2.56611 ± 0.12614	-2.56611 ± 0.12614	-2.56611 ± 0.12614
b3	0 ± 0	0 ± 0	0 ± 0	0 ± 0
b4	0 ± 0	0 ± 0	0 ± 0	0 ± 0
b5	0 ± 0	0 ± 0	0 ± 0	0 ± 0
Reduced Chi-Sqr	0.00347			
R-Square (COD)	0.99979	0.99975	0.99886	0.99934
R-Square (COD)	0.99949			
Adj. R-Square	0.99948			

MOF-303#EDA–pellet

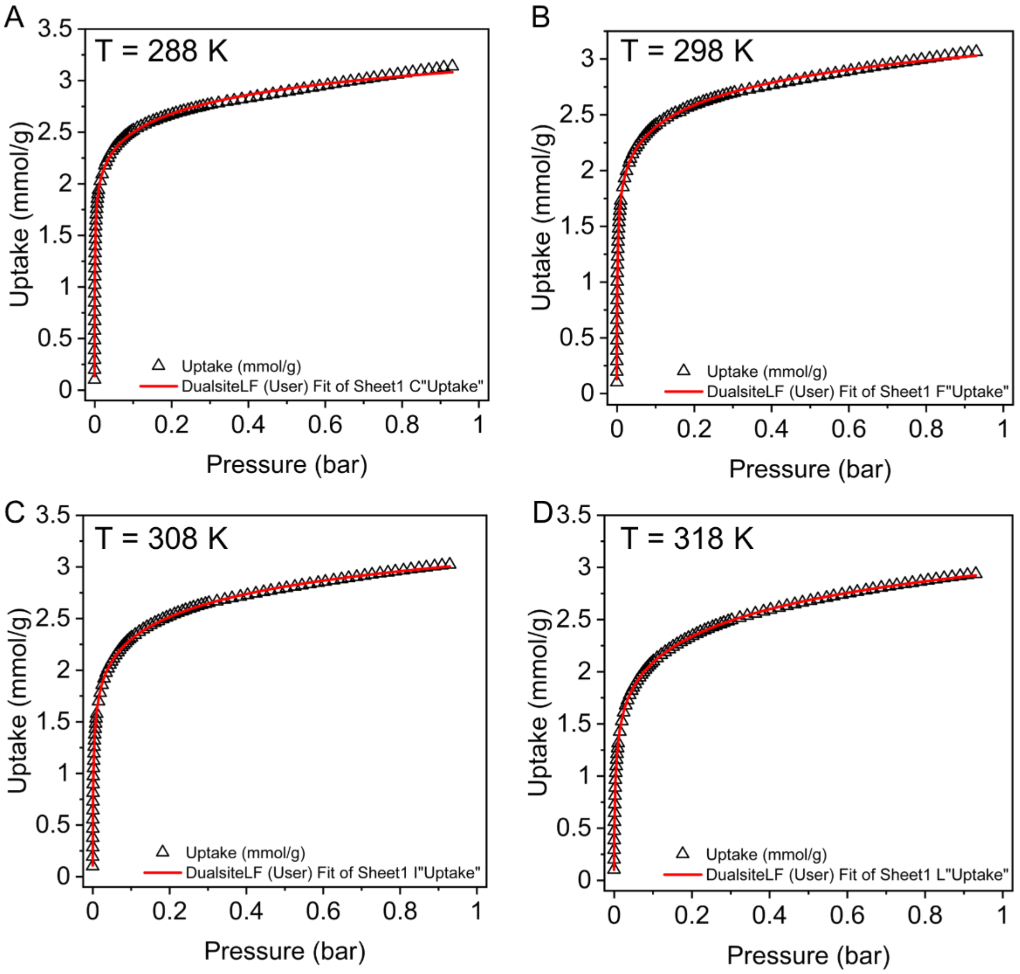

Figure A11 CO_2 adsorption isotherms of MOF-303#EDA-pellet (black triangles) and fitting curves according to the dual-site Langmuir-Freundlich model (red lines) measured at (A) 288 K, (B) 298 K, (C) 308 K, and (D) 318 K.

Table A10 Fitting parameters of the dual-site Langmuir-Freundlich equations for MOF-303#EDA-pellet.

Model	DualsiteLF (User)			
Equation	((q1*k1*x^n1)/(1+k1*x^n1))+((q2*k2*x^n2)/(1+k2*x^n2))			
Plot	288 K	298 K	308 K	318 K
q1	1.48447 ± 0.14643	1.48447 ± 0.14643	1.48447 ± 0.14643	1.48447 ± 0.14643
q2	2.69531 ± 0.29896	2.69531 ± 0.29896	2.69531 ± 0.29896	2.69531 ± 0.29896
k1	825.1216 ± 389.45848	258.70875 ± 87.14691	190.20319 ± 58.68013	95.85132 ± 26.86081
k2	1.49597 ± 0.11827	1.39456 ± 0.10291	1.3521 ± 0.09677	1.21847 ± 0.07941
n1	0.87304 ± 0.05876	0.79427 ± 0.04381	0.8282 ± 0.04327	0.79237 ± 0.03647
n2	0.3876 ± 0.04108	0.41786 ± 0.04208	0.45829 ± 0.04544	0.548 ± 0.05416
Reduced Chi-Sqr	2.72166E-4			
R-Square (COD)	0.99925	0.99951	0.99972	0.99989
R-Square (COD)	0.99958			
Adj. R-Square	0.99956			

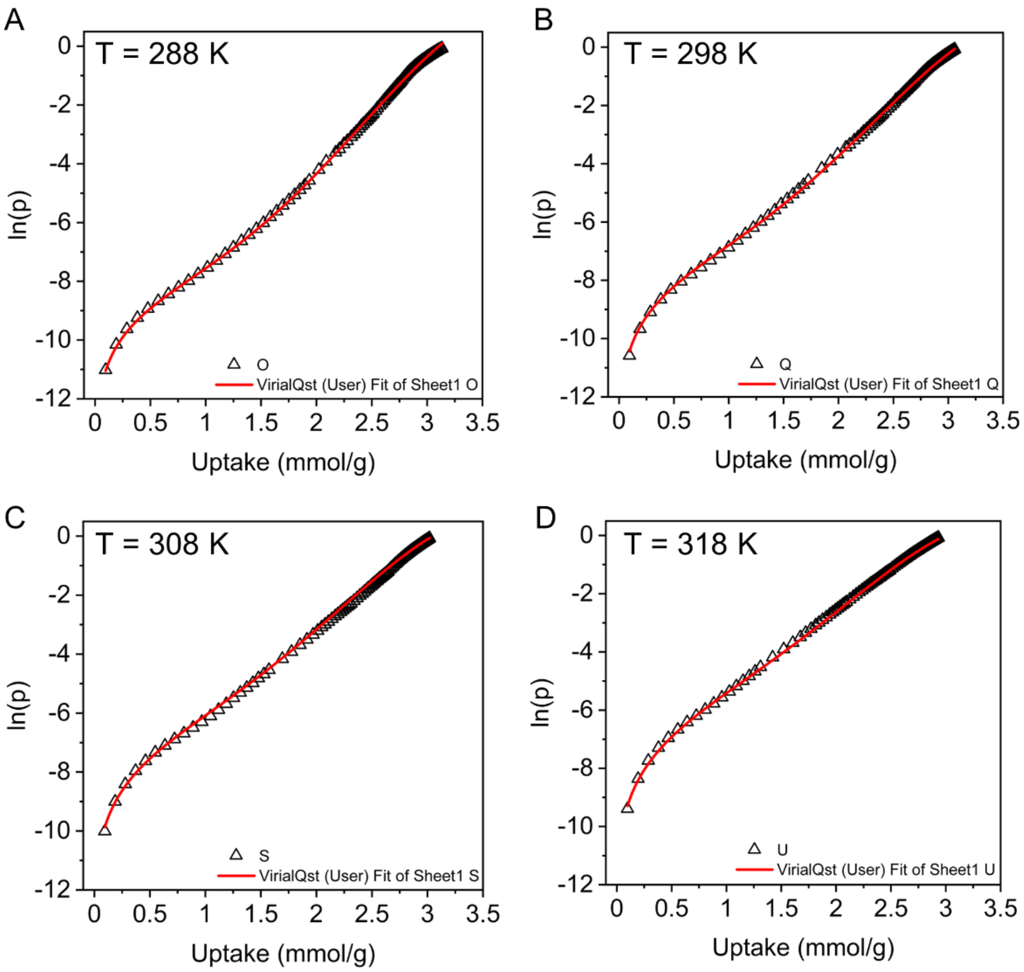

Figure 12 Virial fitting (red line) of the CO_2 adsorption isotherms of MOF-303#EDA-pellet measured at (A) 288 K, (B) 298 K, (C) 308 K, and (D) 318 K.

Table A11 Fitting parameters of the virial equation for MOF-303#EDA-pellet.

Model	VirialQst (User)			
Equation	Qst=-R*(a0+a1*N+a2*N^2+a3*N^3+a4*N^4+a5*N^5+a6*N^6+a7*N^7+a8*N^8); lnp = ln(N) + (1/T)*(-Qst/R) + (b0+b1*N+b2*N^2+b3*N^3+b4*N^4+b5*N^5);			
Plot	288 K	298 K	308 K	318 K
T	288 ± 0	298 ± 0	308 ± 0	318 ± 0
a0	-5044.16068 ± 137.49838	-5044.16068 ± 137.49838	-5044.16068 ± 137.49838	-5044.16068 ± 137.49838
a1	-2680.1772 ± 171.60294	-2680.1772 ± 171.60294	-2680.1772 ± 171.60294	-2680.1772 ± 171.60294
a2	976.83809 ± 60.20038	976.83809 ± 60.20038	976.83809 ± 60.20038	976.83809 ± 60.20038
a3	243.55286 ± 19.59461	243.55286 ± 19.59461	243.55286 ± 19.59461	243.55286 ± 19.59461
a4	-41.99455 ± 2.9071	-41.99455 ± 2.9071	-41.99455 ± 2.9071	-41.99455 ± 2.9071
a5	0 ± 0	0 ± 0	0 ± 0	0 ± 0
a6	0 ± 0	0 ± 0	0 ± 0	0 ± 0
a7	0 ± 0	0 ± 0	0 ± 0	0 ± 0
a8	0 ± 0	0 ± 0	0 ± 0	0 ± 0
b0	8.64518 ± 0.45547	8.64518 ± 0.45547	8.64518 ± 0.45547	8.64518 ± 0.45547
b1	10.80096 ± 0.5682	10.80096 ± 0.5682	10.80096 ± 0.5682	10.80096 ± 0.5682
b2	-4.29247 ± 0.15901	-4.29247 ± 0.15901	-4.29247 ± 0.15901	-4.29247 ± 0.15901
b3	0 ± 0	0 ± 0	0 ± 0	0 ± 0
b4	0 ± 0	0 ± 0	0 ± 0	0 ± 0
b5	0 ± 0	0 ± 0	0 ± 0	0 ± 0
Reduced Chi-Sqr	0.00427			
R-Square (COD)	0.99964	0.99964	0.99863	0.99933
R-Square (COD)	0.99936			
Adj. R-Square	0.99935			

Table A12 Skeletal density measured using He-picnometry and apparent density calculated from He-picnometry and N_2 adsorption isotherms collected at 77 K.

Sample	Skeletal density (g/cm^3)	Apparent density (g/cm^3)
MOF-303	1.91	0.96
MOF-303#EDA	1.65	1.59
MOF-303#EDA - pellet	1.61	1.55

References

[1] Z. Zheng, H. L. Nguyen, N. Hanikel, K. K. Y. Li, Z. Zhou, T. Ma, O. M. Yaghi, “High-yield, green and scalable methods for producing MOF-303 for water harvesting from desert air” Nat. Protoc. **2023**, 18, 136–156.

[2] M. Thommes, K. Kaneko, A. V. Neimark, J. P. Olivier, F. Rodriguez-Reinoso, J. Rouquerol, K. S. W. Sing, “Physisorption of gases, with special reference to the evaluation of surface area and pore size distribution (IUPAC Technical Report)” Pure Appl. Chem. **2015**, 87, 1051–1069.

[3] J. Rouquerol, P. Llewellyn, F. Rouquerol in Stud Surf Sci Catal (Eds.: P.L. Llewellyn, F. Rodriquez-Reinoso, J. Rouqerol, N. Seaton), Elsevier, **2007**, pp. 49–56.

[4] Adsorption by Powders and Porous Solids, Elsevier, **1999**.

[5] A. Nuhnen, C. Janiak, “A practical guide to calculate the isosteric heat/enthalpy of adsorption via adsorption isotherms in metal–organic frameworks, MOFs” Dalton Trans. **2020**, 49, 10295–10307.

[6] A. A. Coelho, “Indexing of powder diffraction patterns by iterative use of singular value decomposition” J. Appl. Crystallogr. **2003**, 36, 86–95.

[7] G. Metz, X. L. Wu, S. O. Smith, “Ramped-Amplitude Cross Polarization in Magic-Angle-Spinning NMR” J. Magn. Reson. A **1994**, 110, 219–227.

[8] B. M. Fung, A. K. Khitrin, K. Ermolaev, “An Improved Broadband Decoupling Sequence for Liquid Crystals and Solids” J. Magn. Reson. **2000**, 142, 97–101.

[9] D. Massiot, F. Fayon, M. Capron, I. King, S. Le Calvé, B. Alonso, J.-O. Durand, B. Bujoli, Z. Gan, G. Hoatson, “Modelling one- and two-dimensional solid-state NMR spectra” Magn. Reson. Chem. **2002**, 40, 70–76.

[10] A. Erba, J. K. Desmarais, S. Casassa, B. Civalleri, L. Donà, I. J. Bush, B. Searle, L. Maschio, L. Edith-Daga, A. Cossard, C. Ribaldone, E. Ascrizzi, N. L. Marana, J.-P. Flament, B. Kirtman, “CRYSTAL23: A Program for Computational Solid State Physics and Chemistry” J. Chem. Theory Comput. **2023**, 19, 6891–6932.

[11] J. P. Perdew, K. Burke, M. Ernzerhof, “Generalized Gradient Approximation Made Simple” Phys. Rev. Lett. **1996**, 77, 3865–3868.

[12] D. Vilela Oliveira, J. Laun, M. F. Peintinger, T. Bredow, “BSSE-correction scheme for consistent gaussian basis sets of double- and triple-zeta valence with polarization quality for solid-state calculations” J. Comput. Chem. **2019**, 40, 2364–2376.

[13] S. Grimme, J. Antony, S. Ehrlich, H. Krieg, “A consistent and accurate ab initio parametrization of density functional dispersion correction (DFT-D) for the 94 elements H-Pu” J. Chem. Phys. **2010**, 132, 154104.

[14] S. F. Boys, F. Bernardi, “The calculation of small molecular interactions by the differences of separate total energies. Some procedures with reduced errors” Mol. Phys. **1970**, 19, 553–566.

[15] J. R. Yates, C. J. Pickard, F. Mauri, “Calculation of NMR chemical shifts for extended systems using ultrasoft pseudopotentials” Phys. Rev. B **2007**, 76, 024401.

[16] A. Sadoc, M. Body, C. Legein, M. Biswal, F. Fayon, X. Rocquefelte, F. Boucher, “NMR parameters in alkali, alkaline earth and rare earth fluorides from first principle calculations” Phys. Chem. Chem. Phys. **2011**, 13, 18539.

[17] G. Hafner J. and Kresse in Properties of Complex Inorganic Solids (Ed.: A. and T.P.E.A. Gonis Antonios and Meike), Springer US, Boston, MA, **1997**, pp. 69–82.

[18] G. P. Francis, M. C. Payne, “Finite basis set corrections to total energy pseudopotential calculations” J. Phys. Condens.: Matter **1990**, 2, 4395–4404.

[19] P. G. Dacosta, O. H. Nielsen, K. Kunc, “Stress theorem in the determination of static equilibrium by the density functional method” J. Phys. C: Solid State Phys. **1986**, 19, 3163.

[20] T. T. T. Nguyen, J.-B. Lin, G. K. H. Shimizu, A. Rajendran, “Separation of CO2 and N2 on a hydrophobic metal organic framework CALF-20” Chem. Eng. J. **2022**, 442, 136263.

[21] Z. Hu, Y. Wang, S. Farooq, D. Zhao, “A highly stable metal-organic framework with optimum aperture size for CO2 capture” AICHE J. **2017**, 63, 4103–4114.

[22] N. S. Wilkins, A. Rajendran, S. Farooq, “Dynamic column breakthrough experiments for measurement of adsorption equilibrium and kinetics” Adsorption **2021**, 27, 397–422.

[23] S. Choi, T. Watanabe, T.-H. Bae, D. S. Sholl, C. W. Jones, “Modification of the Mg/DOBDC MOF with Amines to Enhance CO2 Adsorption from Ultradilute Gases” J. Phys. Chem. Lett. **2012**, 3, 1136–1141.

[24] Y. Cao, F. Song, Y. Zhao, Q. Zhong, “Capture of carbon dioxide from flue gas on TEPA-grafted metal-organic framework Mg2(dobdc)” J. Environ. Sci. **2013**, 25, 2081–2087.

[25] R. L. Siegelman, T. M. McDonald, M. I. Gonzalez, J. D. Martell, P. J. Milner, J. A. Mason, A. H. Berger, A. S. Bhown, J. R. Long, “Controlling Cooperative CO2 Adsorption in Diamine-Appended Mg2(dobpdc) Metal–Organic Frameworks” J. Am. Chem. Soc. **2017**, 139, 10526–10538.

[26] H. Jo, W. R. Lee, N. W. Kim, H. Jung, K. S. Lim, J. E. Kim, D. W. Kang, H. Lee, V. Hiremath, J. G. Seo, H. Jin, D. Moon, S. S. Han, C. S. Hong, “Fine-Tuning of the Carbon Dioxide Capture Capability of Diamine-Grafted Metal–Organic Framework Adsorbents Through Amine Functionalization” ChemSusChem **2017**, 10, 541–550.

[27] W. R. Lee, J. E. Kim, S. J. Lee, M. Kang, D. W. Kang, H. Y. Lee, V. Hiremath, J. G. Seo, H. Jin, D. Moon, M. Cho, Y. Jung, C. S. Hong, “Diamine-Functionalization of a Metal–Organic Framework Adsorbent for Superb Carbon Dioxide Adsorption and Desorption Properties” ChemSusChem **2018**, 11, 1694–1707.

[28] J. Liu, Y. Wei, Y. Zhao, “Trace Carbon Dioxide Capture by Metal–Organic Frameworks” ACS Sustain. Chem. Eng. **2019**, 7, 82–93.

[29] X. Wang, H. Li, X.-J. Hou, “Amine-Functionalized Metal Organic Framework as a Highly Selective Adsorbent for CO2 over CO” J. Phys. Chem. C **2012**, 116, 19814–19821.

[30] L. A. Darunte, A. D. Oetomo, K. S. Walton, D. S. Sholl, C. W. Jones, “Direct Air Capture of CO2 Using Amine Functionalized MIL-101(Cr)” ACS Sustain. Chem. Eng. **2016**, 4, 5761–5768.

[31] H. Li, K. Wang, D. Feng, Y. Chen, W. Verdegaal, H. Zhou, “Incorporation of Alkylamine into Metal–Organic Frameworks through a Brønsted Acid–Base Reaction for CO2 Capture” ChemSusChem **2016**, 9, 2832–2840.

[32] Y. Lin, H. Lin, H. Wang, Y. Suo, B. Li, C. Kong, L. Chen, “Enhanced selective CO2 adsorption on polyamine/MIL-101(Cr) composites” J. Mater. Chem. A **2014**, 2, 14658–14665.

[33] L. A. Darunte, A. D. Oetomo, K. S. Walton, D. S. Sholl, C. W. Jones, “Direct Air Capture of CO2 Using Amine Functionalized MIL-101(Cr)” ACS Sustain. Chem. Eng. **2016**, 4, 5761–5768.

[34] Y. K. Hwang, D. Hong, J. Chang, S. H. Jhung, Y. Seo, J. Kim, A. Vimont, M. Daturi, C. Serre, G. Férey, “Amine Grafting on Coordinatively Unsaturated Metal Centers of MOFs: Consequences for Catalysis and Metal Encapsulation” Angew. Chem., Int. Ed. **2008**, 47, 4144–4148.

[35] A. Das, M. Choucair, P. D. Southon, J. A. Mason, M. Zhao, C. J. Kepert, A. T. Harris, D. M. D’Alessandro, “Application of the piperazine-grafted CuBTTri metal-organic framework in postcombustion carbon dioxide capture” Microporous Mesoporous Mater. **2013**, 174, 74–80.

[36] T. M. McDonald, D. M. D’Alessandro, R. Krishna, J. R. Long, “Enhanced carbon dioxide capture upon incorporation of N,N’-dimethylethylenediamine in the metal–organic framework CuBTTri” Chem. Sci. **2011**, 2, 2022.

[37] O. I.-F. Chen, C.-H. Liu, K. Wang, E. Borrego-Marin, H. Li, A. H. Alawadhi, J. A. R. Navarro, O. M. Yaghi, “Water-Enhanced Direct Air Capture of Carbon Dioxide in Metal–Organic Frameworks” J. Am. Chem. Soc. **2024**, 146, 2835–2844.

[38] A. Justin, J. Espín, I. Kochetygov, M. Asgari, O. Trukhina, W. L. Queen, “A Two Step Postsynthetic Modification Strategy: Appending Short Chain Polyamines to Zn-NH2-BDC MOF for Enhanced CO2 Adsorption” Inorg. Chem. **2021**, 60, 11720–11729.

[39] J.-B. Lin, T. T. T. Nguyen, R. Vaidhyanathan, J. Burner, J. M. Taylor, H. Durekova, F. Akhtar, R. K. Mah, O. Ghaffari Nik, S. Marx, N. Fylstra, S. S. Iremonger, K. W. Dawson, P. Sarkar, P. Hovington, A. Rajendran, T. K. Woo, G. K. H. Shimizu, “A scalable metal organic framework as a durable physisorbent for carbon dioxide capture” Science **2021**, 374(6574), 1464–1469.

[40] Z. Zhou, T. Ma, H. Zhang, S. Chheda, H. Li, K. Wang, S. Ehrling, R. Giovine, C. Li, A. H. Alawadhi, M. M. Abduljawad, M. O. Alawad, L. Gagliardi, J. Sauer, O. M. Yaghi, "Carbon dioxide capture from open air using covalent organic frameworks" Nature **2024**, 635, 96–101
